# Patterns of Health Care Use 5 Years After an Intervention Linking Patients in Addiction Treatment With a Primary Care Practitioner

**DOI:** 10.1001/jamanetworkopen.2022.41338

**Published:** 2022-11-10

**Authors:** Esti Iturralde, Constance M. Weisner, Sara R. Adams, Felicia W. Chi, Thekla B. Ross, Sarah F. Cunningham, Murtuza Ghadiali, Asma H. Asyyed, Derek D. Satre, Cynthia I. Campbell, Stacy A. Sterling

**Affiliations:** 1 Division of Research, Kaiser Permanente Northern California, Oakland; 2 Department of Psychiatry and Behavioral Sciences, Weill Institute for Behavioral Sciences, University of California, San Francisco; 3 The Permanente Medical Group, Oakland, California

## Abstract

**Importance:**

Substance use disorders are associated with high rates of emergency department (ED) use and challenges engaging with primary care services.

**Objective:**

To examine 5-year health care engagement and utilization outcomes for participants in the LINKAGE trial, given previously reported associations of LINKAGE with improved care engagement in the short term.

**Design, Setting, and Participants:**

In this post hoc analysis of a nonrandomized controlled trial, participants were assigned to the LINKAGE or usual care (UC) groups using a nonrandomized 3-month alternating off and on strategy over 30 months. Baseline through 5-year follow-up data were collected from April 2011 to October 2018. The trial was conducted at an urban outpatient addiction treatment clinic within a large health system among patients newly enrolled in addiction treatment. Data analysis was conducted from April 2021 to February 2022.

**Intervention:**

The LINKAGE intervention included 6 group-based sessions emphasizing patient agency, skill, and motivation in navigating health care services as well as a facilitated telephone or email connection with a primary care practitioner. The UC group received medical education.

**Main Outcomes and Measures:**

Substance use problem discussions with primary care practitioners (by patient self-report at 1-, 2-, and 5-year follow-up interview) and annual use of the electronic patient portal, primary care, and ED based on electronic health records.

**Results:**

A total of 503 participants, with a mean (SD) age of 42 (12) years, 346 (69%) male participants and 37 (7%) African American, 34 (7%) Asian, and 101 (20%) Hispanic participants, were assigned to LINKAGE (252 participants) or UC (251 participants). Compared with UC participants, LINKAGE participants were significantly more likely to discuss substance use problems with a primary care practitioner at 1-year follow-up (risk ratio [RR], 1.30; 95% CI, 1.03-1.65; *P* = .03) and use the electronic patient portal at 1- and 2-year follow-up (eg, messaging clinicians at 2 years: RR, 1.24; 95% CI, 1.04-1.47; *P* = .02). The LINKAGE group had small, statistically significant 5-year annual increases in primary care use (RR, 1.03; 95% CI, 1.003-1.07; *P* = .03) and significant annual decreases in substance-related ED use (RR, 0.79; 95% CI, 0.64-0.97; *P* = .03), relative to UC. The LINKAGE group did not significantly differ from the UC group on other types of ED utilization.

**Conclusions and Relevance:**

In this study, a patient activation intervention embedded in outpatient addiction treatment was associated with sustained improvements in health care engagement beyond previously reported 6-month outcomes and with long-term improvements in health care use patterns.

**Trial Registration:**

ClinicalTrials.gov Identifier: NCT01621711

## Key Points

**Question:**

Is an intervention to connect patients undergoing addiction treatment to primary care practitioners associated with long-term improved engagement with primary care?

**Findings:**

This post hoc analysis of 5-year follow-up data from a nonrandomized controlled trial with 503 participants found that LINKAGE intervention participants, relative to usual care, were more likely to discuss substance use problems with primary care practitioners, use the electronic patient portal, and have relative annual increases in primary care use and relative annual decreases in substance use–related emergency department utilization.

**Meaning:**

In this study, a patient activation intervention provided with addiction treatment was associated with improved long-term health care engagement patterns.

## Teaser

This nonrandomized controlled trial examines 5-year health care engagement and utilization outcomes for participants in a patient activation intervention vs usual care.

## Introduction

More than 20 million individuals in the United States have a substance use (SU) disorder.^1^ SU problems are associated with elevated risk for acute and chronic medical conditions^2,3^ and with costly medical use, including high rates of potentially preventable emergency department (ED) visits.^4,5,6,7^ Patients who receive addiction services visit the ED at high rates even after starting treatment.^8^ Regular visits to primary care after addiction treatment are associated with reduced medical costs and improved SU outcomes.^9,10,11,12^ By addressing health concerns and intervening before health problems become more serious, primary care services may augment the benefits of addiction treatment while reducing potentially invasive, avoidable, or stigmatizing^13^ care in the ED.

Unfortunately, psychosocial and systemic barriers inhibit primary care engagement after addiction treatment. Patients may have difficulty establishing rapport with primary care practitioners due to self-stigma^14^ or fears of discrimination due to stigma surrounding SU problems.^15,16^ Addiction treatment staff may have difficulty coordinating follow-up with primary care practitioners because of privacy laws and policies restricting health information sharing.^17^ There is a need for interventions to improve patients’ engagement with the health care system during and after addiction treatment, both by enhancing integration across addiction and primary care services and by fostering a stronger, recovery-focused partnership between patients and primary care practitioners. In a previous clinical trial,^18^ a 6-session, group-based, patient skill-building intervention embedded in outpatient addiction treatment, which included a facilitated connection by phone or email with a primary care provider (LINKAGE), was associated with increased health care engagement behaviors. At 6 months after the intervention, LINKAGE participants were more likely than those in usual care (UC) to use electronic patient portal tools, such as refilling prescriptions and sending electronic messages to a clinician.

Proactive SU discussions and other health care engagement behaviors during the critical 6 months after starting addiction treatment may help individuals develop and maintain long-term engagement with primary care services and reduce the need for ED visits. To assess these potential sequelae, we conducted a post hoc analysis using 5 years of follow-up data from participant interviews and electronic health records (EHRs) to examine whether the short-term benefits of the trial would extend to long-term changes in health care system engagement and utilization. We hypothesized that the LINKAGE intervention would be associated with sustained improvements in participants’ discussion of SU problems with a primary care practitioner and in use of electronic patient portal tools, increased primary care use, and decreased overall and preventable ED utilization, relative to UC.

## Methods

### Study Design, Eligibility, and Procedures

As reported previously,^18^ the original clinical trial enrolled 503 participants from the San Francisco outpatient addiction treatment clinic of Kaiser Permanente Northern California (KPNC), a health care system caring for more than 4.4 million members regionwide. Member sociodemographic characteristics are diverse and representative of Northern California overall.^19^ Trial participants were recruited between April 2011 and October 2013.

The trial was based in a single, large outpatient clinic. To minimize risk for contamination across study groups within the site (as participants attended other parts of the addiction treatment program together), we used a nonrandomized allocation strategy based on when the participant entered addiction treatment. A participant was assigned to the LINKAGE group (addiction treatment with LINKAGE) if they entered treatment during a LINKAGE period and assigned to the UC group (addiction treatment with medical education) if they entered treatment during a UC period. Specifically, assignment to LINKAGE or UC switched every 3 months for a duration of 30 months (5 alternating 3-month periods per condition). Once assigned, participants stayed in the same condition. In the current study, we report participants’ follow-up data collected during the 5 years after study enrollment, with final data collected in October 2018.

Eligible participants were adults age 18 years or older attending the clinic’s outpatient addiction treatment program and were cleared by their physician for participation. Overall, 49 individuals with severe cognitive disability or psychiatric impairment (eg, manic episode), who were evenly distributed across conditions, were excluded.

Participants provided written informed consent prior to study enrollment and completed a baseline computer-based assessment. Follow-up telephone interviews were conducted at 1, 2, and 5 years after the study enrollment date by assessors blinded to study condition. EHR data were obtained at baseline (during the 1 year prior to study enrollment) and annually during a 5-year follow-up period. Participants received monetary incentives for completing interviews and were not blinded to study condition. The institutional review boards of KPNC and the University of California, San Francisco, approved the study, which received a National Institutes of Health Certificate of Confidentiality. We followed reporting guidelines of the Transparent Reporting of Evaluations with Nonrandomized Designs (TREND) group.^36^ The trial protocol appears in Supplement 1.

### UC and LINKAGE Intervention

Participants in both LINKAGE and UC received outpatient addiction treatment 4 days per week, starting with a 10-day stabilization program followed by a 6-week program of psychotherapy groups, individual counseling, 12-step meetings, and alcohol and drug screening. During this 6-week period, the intervention group received LINKAGE and the UC group received medical education. Medical education informed participants about how to prevent or manage health conditions that are commonly associated with SU problems. Both LINKAGE and medical education groups were delivered by a clinical psychologist or licensed therapist in 6 weekly 45-minute, manual-guided group sessions. Physician appointments and medications were available as needed. All participants had access to the KPNC electronic patient portal, an online system allowing members to view their health information, refill prescriptions, and exchange messages with clinicians.

The LINKAGE intervention design^18^ drew from patient activation and engagement principles across research literatures on chronic health condition management.^20,21,22,23,24,25,26^ LINKAGE sessions included in-session practice of patient-clinician communication strategies, electronic patient portal navigation skills, and development of recovery- and health-related goals. LINKAGE interventionists facilitated a phone call or email in which the participant discussed recovery and health goals with their primary care practitioner.

### Measures

During the baseline interview, we collected sociodemographic data and assessed SU disorder type based on the Diagnostic Interview Schedule for Psychoactive Substance Dependence in the *Diagnostic and Statistical Manual of Mental Disorders* (Fourth Edition).^27,28^ Self-reported race and ethnicity data were collected for descriptive purposes using the following categories: African American, American Indian or Alaska Native, Asian, Hispanic, White, and other. We calculated the Charlson Comorbidity Index^29,30^ from baseline EHR data to characterize the sample’s disease burden. The primary outcome was self-reported discussion of an SU problem with a primary care practitioner, assessed in 1-, 2-, and 5-year follow-up interviews using the item, “During the past 6 months have you talked to your primary care provider about alcohol or drug problems?” (yes or no). As secondary health care engagement outcomes, we assessed participants’ use of the electronic patient portal per follow-up year from EHR records. Specifically, we examined logins, online medication refills, and electronic messages sent to health care practitioners (any or none).

We examined health care use as additional secondary outcomes. EHR data provided an annual measure of primary care visits (any or none). We used EHR and administrative claims data to assess any visit to a KPNC or non-KPNC ED; visits were additionally coded by primary diagnosis as emergent, nonemergent, or SU-related according to the validated New York University Classification Algorithm.^31,32^ Emergent visits would require immediate care. Nonemergent visits would not be expected to require ED care. SU-related diagnoses involved alcohol or drugs. eFigure 1 in Supplement 2 includes examples.

### Statistical Analysis

Analyses followed an intention-to-treat framework. Power calculations were 2-sided, with a significance level of .05. We present a conservative power estimation for analysis of a single time point using logistic regression. Assuming an expected 40% of UC participants with recent discussion of SU problems with a primary care practitioner, we had more than 0.80 power to test a 14–percentage point difference between conditions with a sample size of 225 per group. With this sample size, we had more than 0.80 power to detect a 12–,10–, and 12–percentage point difference between conditions on electronic patient portal logins, primary care use, and ED utilization, assuming UC proportions of 70%, 80%, and 30%. Expected outcome levels were based on baseline data, 6-month follow-up findings,^18^ and theoretical considerations (eg, regression to the mean).

We evaluated through risk ratios (RRs) the association between group assignment and participants’ health care engagement and utilization using a modified Poisson generalized estimating equations (GEEs) framework, with an unstructured correlation matrix to account for repeated observations^33^ and adjusting for baseline covariates of age, Charlson Comorbidity Index, and emergent-type ED use.

For each health care engagement outcome (SU problem discussions, electronic patient portal outcomes), we estimated group differences from individual modified Poisson regression models at each follow-up time point to assess fluctuations in group level over time, adjusting for our baseline covariates. For each outcome, we also estimated a GEE model evaluating group differences during the 5-year follow-up period, adjusting for (linear) time and our baseline covariates. Marginal percentage estimates by group were plotted from a GEE model per outcome, including time as a categorical variable, a time × group interaction term, and our baseline covariates.

For health care utilization, we expected gradual changes over time. In a GEE model per outcome, we estimated and plotted trends by group over the 5-year follow-up period by including (linear) time, a time × group interaction term, and our baseline covariates. We then evaluated group differences over the 5-year follow-up period with GEE models per outcome including (linear) time, no interaction term, and our baseline covariates.

We could not observe EHR-ascertained outcomes during time periods when participants lacked health plan coverage. Therefore, we treated as missing all observations for follow-up years with less than 9 months health plan coverage. This approach enabled us to include in GEE models all available observations per participant (ie, during years with ≥9 months of health plan coverage). To examine potential bias related to missing follow-up data, we conducted several sensitivity analyses: (1) we reran models using multiple imputation with chained equations^34^ (25 data sets employing predictive mean matching^35^), with prediction from all available baseline and follow-up observations, and (2) we reran models with complete cases, ie, including participants who were alive and had at least 9 months health plan coverage in all follow-up years. We conducted study analyses in Stata version 16.0 (StataCorp). Statistical significance was set at *P* < .05, and all tests were 2-tailed. 

## Results

### Participant Characteristics and Study Flow

The 503 participants had a mean (SD) age of 42 (12) years (range, 18-73 years); 346 (69%) were male participants; there were 37 (7%) African American, 10 (2%) American Indian or Alaska Native, 34 (7%) Asian, 101 (20%) Hispanic, and 306 (61%) White participants; 225 (45%) earned less than $50 000 annually income; and 203 (40%) had a high school education or less (Table 1). Most participants (329 [65%]) had an alcohol use disorder; 232 (46%) had a drug use disorder. In the prior year, more than 80% (367 of 431 [85%]) visited primary care and 50% (214) visited the ED. Baseline characteristics were similar across study groups.

**Table 1.** LINKAGE Trial Participant Characteristics by Study Group

Characteristic	Participants, No. (%)		*P* value
	LINKAGE (n = 252)	Usual care (n = 251)	
**At study enrollment**			
Sex			
Male	175 (69.4)	171 (68.1)	.77
Female	77 (30.6)	80 (31.9)	
Age, mean (SD), y	41.4 (11.6)	43.5 (12.0)	.07
Race and ethnicity			
African American	17 (6.7)	20 (8.0)	.96
Asian	16 (6.3)	18 (7.2)	
Hispanic	49 (19.4)	52 (20.7)	
American Indian or Alaska Native	6 (2.4)	4 (1.6)	
White	156 (61.9)	150 (59.8)	
Other^a^	8 (3.2)	7 (2.8)	
Education			
≤High school graduate or equivalent	96 (38.1)	107 (42.6)	.54
Associate in arts, associate in science, or technical school	49 (19.4)	52 (20.7)	
College or higher	107 (42.5)	92 (36.7)	
Annual household income <$50 000	112 (44.4)	113 (45.0)	.93
Substance dependence			
Alcohol	170 (67.5)	159 (63.3)	.35
Drug	124 (49.2)	108 (43.0)	.18
Opioid	44 (17.5)	37 (14.7)	.47
Cocaine	46 (18.3)	33 (13.1)	.14
Marijuana	31 (12.3)	30 (12.0)	>.99
Amphetamine	27 (10.7)	32 (12.7)	.49
Sedative	18 (7.1)	13 (5.2)	.46
Charlson Comorbidity Index, mean (SD)	0.46 (1.3)	0.59 (1.3)	.07
**During 1 y prior to study enrollment^b^**			
Electronic patient portal use, any			
Logins	139 (66.8)	143 (64.1)	.61
Online medication refills	84 (40.4)	82 (36.8)	.49
Secure messages sent to health providers	102 (49.0)	110 (49.3)	>.99
Health care service visits, any			
Primary care	171 (82.2)	196 (87.9)	.11
Emergency department			
Any	101 (48.6)	113 (50.7)	.70
Emergent type	10 (4.8)	28 (12.6)	.01
Nonemergent type	32 (15.4)	42 (18.8)	.37
Substance-related type	33 (15.9)	42 (18.8)	.45

^a^ Other race and ethnicity included non-Hispanic participants who indicated having “more than one racial/ethnic background” in the baseline interview.

^b^ Data for 208 participants in LINKAGE and 223 participants in usual care; data were missing due to insufficient health system enrollment during the 1 year prior to study start, resulting in no health care utilization data.

**Figure 1.**
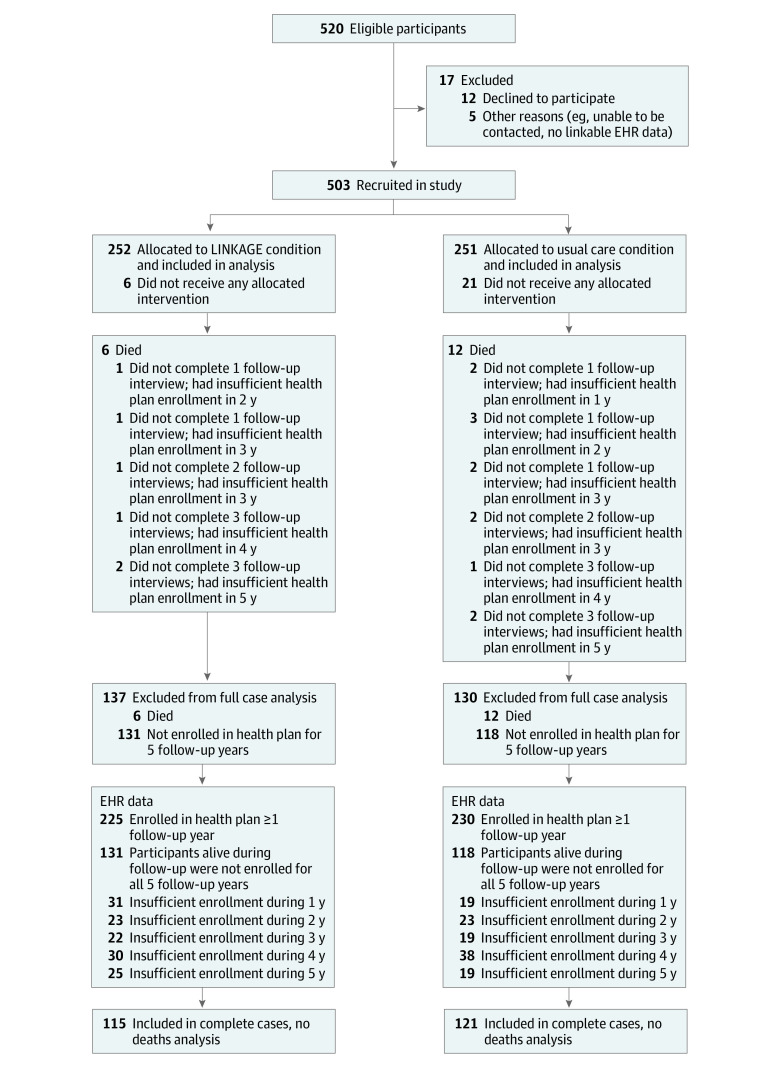
Study Flow Diagram for the LINKAGE Study EHR indicates electronic health record.

Eighteen participants (4%) died during the follow-up period (Figure 1). No interview data were collected for 77 participants (15%), of whom 6 died, and no EHR data were available (due to insufficient health plan coverage) for 48 participants (10%), of whom 4 died. Complete 5-year follow-up data were available for 236 participants (47%). There were no significant differences in attrition type between study groups. Baseline age and electronic patient portal logins were inversely associated with missing follow-up. These and all other study variables were included as factors for multiple imputation models used in sensitivity analyses.

### Health Care Engagement

Across the 1-, 2-, and 5-year follow-up time points (Table 2), LINKAGE participants were more likely to report SU problem discussions with primary care practitioners, but the difference was only statistically significant at 1 year (LINKAGE, 89 of 186 [47.8%]; UC, 72 of 194 [37.1%]; risk ratio [RR], 1.30; 95% CI, 1.03-1.65; *P* = .03). Across the 5 follow-up years, LINKAGE participants were more likely to log into the electronic patient portal (RR, 1.18; 95% CI, 1.08-1.29; *P* < .001), use it to refill medications (RR, 1.38; 95% CI, 1.22-1.56; *P* < .001), and send secure messages to clinicians (RR, 1.18; 95% CI, 1.05-1.33; *P* = .01). In time point–specific analyses, the LINKAGE group maintained higher rates of electronic patient portal use across years, with statistical significance at follow-up years 1 and 2 (eg, year-2 logins: 139 of 175 [79.4%] vs 123 of 182 [67.6%]; RR, 1.17; 95% CI, 1.03-1.33; *P* = .01). Models using multiply imputed data or including participants with complete data produced similar results on health care engagement outcomes (eTables 1 and 2 in Supplement 2).

**Table 2.** Health System Engagement by Study Group

Time	Participants with any, %		Group difference^a^	
	LINKAGE group	Usual care group	RR (95% CI)	*P* value
**Substance use discussion with primary care practitioner^b^**				
Year 1	89/186 (47.8)	72/194 (37.1)	1.30 (1.03-1.65)	.03
Year 2	62/164 (37.8)	56/166 (33.7)	1.14 (0.85-1.53)	.39
Year 3	NA	NA	NA	NA
Year 4	NA	NA	NA	NA
Year 5	24/122 (19.7)	17/116 (14.7)	1.33 (0.76-2.33)	.33
All years^c^	124/209 (59.3)	109/217 (50.2)	1.20 (0.99-1.45)	.07
**Annual electronic patient portal login**				
Year 1	181/206 (87.9)	144/221 (65.2)	1.34 (1.20-1.50)	<.001
Year 2	139/175 (79.4)	123/182 (67.6)	1.17 (1.03-1.33)	.01
Year 3	127/164 (77.4)	119/164 (72.6)	1.06 (0.93-1.20)	.38
Year 4	117/153 (76.5)	105/144 (72.9)	1.05 (0.92-1.19)	.51
Year 5	120/152 (78.9)	105/141 (74.5)	1.07 (0.94-1.21)	.32
All years^c^	181/206 (87.9)	144/221 (65.2)	1.18 (1.08-1.29)	<.001
**Online medication refills**				
Year 1	164/206 (79.6)	100/221 (45.2)	1.75 (1.49-2.06)	<.001
Year 2	119/175 (68.0)	97/182 (53.3)	1.27 (1.07-1.51)	.01
Year 3	105/164 (64.0)	93/164 (56.7)	1.13 (0.94-1.34)	.19
Year 4	98/153 (64.1)	83/144 (57.6)	1.12 (0.93-1.34)	.24
Year 5	98/152 (64.5)	75/141 (53.2)	1.22 (1.01-1.49)	.04
All years^c^	192/225 (85.3)	158/230 (68.7)	1.38 (1.22-1.56)	<.001
**Secure electronic messages sent to health care professional**				
Year 1	144/206 (69.9)	115/221 (52.0)	1.34 (1.15-1.57)	<.001
Year 2	116/175 (66.3)	98/182 (53.8)	1.24 (1.04-1.47)	.02
Year 3	106/164 (64.6)	92/164 (56.1)	1.16 (0.97-1.38)	.10
Year 4	98/153 (64.1)	88/144 (61.1)	1.05 (0.88-1.25)	.59
Year 5	100/152 (65.8)	85/141 (60.3)	1.09 (0.91-1.30)	.35
All years^c^	186/225 (82.7)	165/230 (71.7)	1.18 (1.05-1.33)	.01

Abbreviations: ED, emergency department; NA, not applicable; RR, risk ratio.

^a^ Group differences were estimated from a modified Poisson regression model per follow-up time point, adjusting for baseline age, Charlson Comorbidity Index, and emergent-type ED use.

^b^ Telephone interviews were conducted at 1, 2, and 5 years after study enrollment.

^c^ Group differences were estimated from a modified Poisson generalized estimating equations model, adjusting for (linear) time and baseline age, Charlson Comorbidity Index, and emergent-type ED use.

### Primary Care and Emergency Department Utilization

**Figure 2.**
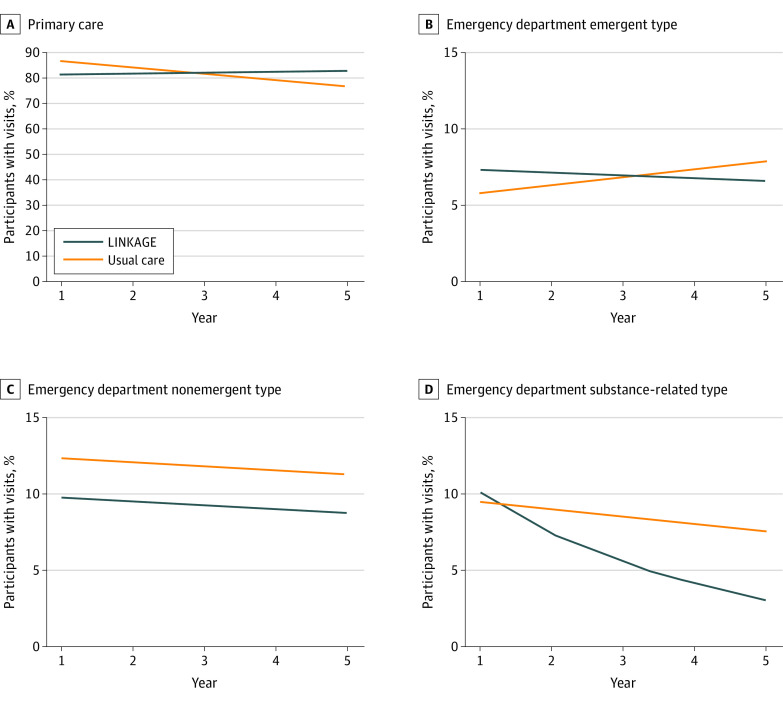
Estimated Percentage of Participants With Primary Care and Emergent, Nonemergent, and Substance-Related Emergency Department Visits Per Year of Study Follow-up, by Group Marginal percentage estimates by group were plotted from a modified Poisson generalized estimating equations model per outcome adjusting for (linear) time, a time × group interaction term, age, Charlson Comorbidity Index, and baseline emergent-type emergency department use.

Compared with UC, LINKAGE participants had significant relative annual increases in primary care use (RR, 1.03; 95% CI, 1.003-1.067; *P* = .03) (Figure 2 and Table 3). The overall rate of primary care use across the 5 years did not significantly differ between the 2 groups (LINKAGE, 217 of 225 [96.4%]; UC, 222 of 230 [96.5%]; RR, 0.99; 95% CI, 0.94-1.05; *P* = .81).

**Table 3.** Five-Year Health Care Use Associated With Study Group

Time	Health care service use, %		Group differences in annual change over the 5 y since study enrollment^a^		Time-adjusted group differences across 5 y since study enrollment^b^	
	LINKAGE	Usual care	Risk ratio (95% CI)	*P* value	Risk ratio (95% CI)	*P* value
**Primary care**						
Year 1	173/206 (84.0)	192/221 (86.9)	1.03 (1.003-1.067)	.03	0.99 (0.94-1.05)	.81
Year 2	142/175 (81.1)	153/182 (84.1)				
Year 3	121/164 (73.8)	136/164 (82.9)				
Year 4	130/153 (85.0)	118/145 (81.4)				
Year 5	125/152 (82.2)	107/142 (75.4)				
Cumulative	217/225 (96.4)	222/230 (96.5)				
**Any ED**						
Year 1	64/206 (31.1)	78/221 (35.3)	0.97 (0.89-1.07)	.57	0.92 (0.75-1.12)	.39
Year 2	49/175 (28.0)	57/182 (31.3)				
Year 3	44/164 (26.8)	42/164 (25.6)				
Year 4	38/153 (24.8)	46/145 (31.7)				
Year 5	40/152 (26.3)	47/142 (33.1)				
Cumulative	128/225 (56.9)	139/230 (60.4)				
**Any emergent type ED**						
Year 1	17/206 (8.3)	16/221 (7.2)	0.90 (0.73-1.12)	.34	1.02 (0.68-1.53)	.93
Year 2	14/175 (8.0)	17/182 (9.3)				
Year 3	8/164 (4.9)	6/164 (3.7)				
Year 4	13/153 (8.5)	12/144 (8.3)				
Year 5	11/152 (7.2)	15/141 (10.6)				
Cumulative	47/225 (20.9)	47/230 (20.4)				
**Any nonemergent type ED**						
Year 1	24/206 (11.7)	31/221 (14.0)	1.00 (0.84-1.19)	.98	0.79 (0.58-1.08)	.14
Year 2	11/175 (6.3)	22/182 (12.1)				
Year 3	19/164 (11.6)	23/164 (14.0)				
Year 4	11/153 (7.2)	23/144 (16.0)				
Year 5	16/152 (10.5)	16/141 (11.3)				
Cumulative	59/225 (26.2)	77/230 (33.5)				
**Any substance-related type ED**						
Year 1	19/206 (9.2)	26/221 (11.8)	0.79 (0.64-0.97)	.03	0.82 (0.51-1.32)	.41
Year 2	16/175 (9.1)	12/182 (6.6)				
Year 3	10/164 (6.1)	16/164 (9.8)				
Year 4	2/153 (1.3)	14/144 (9.7)				
Year 5	6/152 (3.9)	9/141 (6.4)				
Cumulative	35/225 (15.6)	43/230 (18.7)				

Abbreviation: ED, emergency department.

^a^ Group × linear time effect estimate; modified Poisson generalized estimating equations models included (linear) time, a time × group interaction term, and baseline age, Charlson Comorbidity Index, and emergent-type ED use.

^b^ Time-adjusted group effect estimate; modified Poisson generalized estimating equations models included (linear) time, no interaction term, and baseline age, Charlson Comorbidity Index, and emergent-type ED use.

LINKAGE participants had significant annual declines in substance-related ED use over 5 years compared with UC participants (RR, 0.79; 95% CI, 0.64-0.97; *P* = .03). The overall 5-year rates in this outcome did not significantly differ between groups (LINKAGE, 35 [15.6%]; UC, 43 [18.7%]; RR, 0.82; 95% CI, 0.51-1.32; *P* = .41). The LINKAGE and UC groups did not significantly differ in overall or emergent-type ED use, when considering time trend or 5-year rates. The LINKAGE group had lower nonemergent ED use over 5 years compared with UC (59 [26.2%] vs 77 [33.5%]), but this difference was not statistically significant (RR, 0.79; 95% CI, 0.58-1.08; *P* = .14). Five-year change in nonemergent ED use did not significantly differ between groups.

Models using multiply imputed data or including participants with complete data produced similar results on primary care and ED use outcomes (eTables 3 and 4 in Supplement 2). In complete cases analyses, LINKAGE participants had significantly lower nonemergent type ED use than UC participants when examined across the 5-year follow-up (RR, 0.60; 95% CI, 0.39-0.93; *P* = .02).

## Discussion

LINKAGE, a group intervention focusing on patient activation and health care engagement as part of addiction treatment, was associated with positive health care engagement behaviors up to 5 years after treatment. Participants were more likely to discuss SU problems with their primary care practitioner and to use the electronic patient portal to address health needs, such as refilling medications and communicating with health care professionals. LINKAGE participants also saw gradual, desirable changes in modifiable health care use patterns (ie, stable primary care use and declining substance-related ED use over 5 years), whereas UC participants had declining primary care use and stable substance-related ED use over the same time frame. Among participants who maintained health system enrollment during follow-up, LINKAGE patients were less likely than those in UC to visit the ED for diagnoses not typically requiring emergency care.

This study extends the results of the original LINKAGE trial,^18^ which found higher 6-month intervention group rates of SU problem discussions with primary care practitioners and participants’ electronic patient portal use. By teaching participants skills in communicating with health care professionals and navigating personal health information, the LINKAGE intervention sought to increase individuals’ capacity to engage proactively with the health care system. An expectation of the original study was that this ongoing health care engagement (via use of the patient portal for communication and engaging with health information options, as well as primary care visits) would result in positive longer-term health behavior.

A key intervention component was planning for and facilitating open dialogue between participants and primary care practitioners regarding recovery-related health goals, mitigating SU-associated stigma, and circumventing health system barriers to sharing information between addiction treatment and primary care services.^17^ Patients who have more frequent and effective interactions with primary care practitioners may be less likely to use the ED for nonurgent needs and may have fewer alcohol- or drug-related concerns warranting an ED visit. The ED utilization changes seen in the current study align with past observational data finding that consistent primary care use after addiction treatment is associated with improvements in both health care costs and addiction treatment outcomes.^9,10,11,12^

Past research suggests an association between increased electronic patient portal use and improved self-management of diabetes and cardiovascular disease risk factors, although causal mechanisms remain poorly understood.^37,38,39^ Future research should examine the potential health benefits of increasing health care engagement among individuals with SU problems given the high rates of physical health morbidity and mortality in this population.^2,3,40^ Cost-effectiveness analysis would clarify whether increased health care engagement is accompanied by cost savings from prevention of high-resource utilization. LINKAGE participation was not associated with reductions in overall or emergent type ED use, suggesting that additional interventions are needed to prevent and manage health conditions among people with SU problems. There also remains a need to understand how to improve SU outcomes as part of efforts to increase patients’ health care engagement. The prior LINKAGE trial found no group differences on drug or alcohol use outcomes,^18^ nor did 2 other intervention studies^41,42^ seeking to increase addiction patients’ receipt of primary care services. The current study did find declines in substance-related ED use over 5 years, which may be considered a proxy for less substance use-related harm.

### Limitations

This study has limitations. The original clinical trial used an alternating 3-month allocation strategy between groups rather than randomization, introducing potential bias. This nonrandomized approach was necessary to control for clinic effects and minimize risk for contamination across conditions within the single study site where patients would be expected to interact often due to group-based treatment. Although the sample was socioeconomically diverse, more than 60% of participants were White. The study sample size did not allow us to evaluate chronic disease screening or outcomes given participant heterogeneity.

## Conclusions

This study addressed a need for intervention models to increase patients’ engagement and destigmatize SU-focused conversations with primary care practitioners during and after addiction treatment. We have found that a group-based patient activation intervention embedded in outpatient addiction treatment was associated with positive outcomes at 6 months and had benefits continuing over 5 years, including more SU-related communication with primary care practitioners, an increased trend in primary care utilization, and reduction in potentially preventable ED visits. Electronic health records tethered to patient portals have become widespread across health systems,^43,44,45^ providing an opportunity to implement the LINKAGE intervention in many settings. The intervention is manual-based and can be delivered as part of standard, group-based addiction treatment by existing clinic staff. Addiction treatment offers a critical opportunity to engage patients in recovery-focused health goals, with potential to improve health care quality and outcomes.

## Notes

Supplement 1.
Trial Protocol

Supplement 2. **eFigure 1.** Most Common Participant Emergency Department Diagnoses Coded as Emergent, Nonemergent, and Substance Use-Related **eFigure 2. **Estimated Percentage of Participants by Group With Health Care System Engagement per Year of Study Follow-up **eTable 1. **Health System Engagement Associated With Study Group Using Multiply Imputed Data **eTable 2. **Health System Engagement Associated With Study Group, Complete Cases, No Deaths **eTable 3. **Five-Year Health Care Use by Study Groups, Multiply Imputed Data **eTable 4.** Five-Year Health Care Use by Study Group, Complete Cases, No Deaths

Supplement 3.
Data Sharing Statement

## References

[1] U.S. Department of Health and Human Services (HHS) Office of the Surgeon General. Facing Addiction in America: the Surgeon General’s Report on Alcohol, Drugs, and Health. Department of Health and Human Services; 2016. 28252892

[2] Mertens JR, Lu YW, Parthasarathy S, Moore C, Weisner CM. Medical and psychiatric conditions of alcohol and drug treatment patients in an HMO: comparison with matched controls. Arch Intern Med. 2003;163(20):2511-2517. doi:10.1001/archinte.163.20.2511 14609789

[3] Young JQ, Kline-Simon AH, Mordecai DJ, Weisner C. Prevalence of behavioral health disorders and associated chronic disease burden in a commercially insured health system: findings of a case-control study. Gen Hosp Psychiatry. 2015;37(2):101-108. doi:10.1016/j.genhosppsych.2014.12.005 25578791

[4] McLellan AT, Starrels JL, Tai B, . Can substance use disorders be managed using the chronic care model? review and recommendations from a NIDA Consensus Group. Public Health Rev. 2014;35(2):8. doi:10.1007/BF03391707 26568649 PMC4643942

[5] Cederbaum JA, Guerrero EG, Mitchell KR, Kim T. Utilization of emergency and hospital services among individuals in substance abuse treatment. Subst Abuse Treat Prev Policy. 2014;9(1):16. doi:10.1186/1747-597X-9-16 24708866 PMC3992150

[6] Cherpitel CJ, Ye Y. Drug use and problem drinking associated with primary care and emergency room utilization in the US general population: data from the 2005 national alcohol survey. Drug Alcohol Depend. 2008;97(3):226-230. doi:10.1016/j.drugalcdep.2008.03.033 18499355 PMC3007592

[7] Giannouchos TV, Washburn DJ, Kum H-C, Sage WM, Ohsfeldt RL. Predictors of multiple emergency department utilization among frequent emergency department users in 3 states. Med Care. 2020;58(2):137-145. doi:10.1097/MLR.0000000000001228 31651740

[8] Parthasarathy S, Weisner C, Hu T-W, Moore C. Association of outpatient alcohol and drug treatment with health care utilization and cost: revisiting the offset hypothesis. J Stud Alcohol. 2001;62(1):89-97. doi:10.15288/jsa.2001.62.89 11271969

[9] Chi FW, Parthasarathy S, Mertens JR, Weisner CM. Continuing care and long-term substance use outcomes in managed care: early evidence for a primary care-based model. Psychiatr Serv. 2011;62(10):1194-1200. doi:10.1176/ps.62.10.pss6210_1194 21969646 PMC3242696

[10] Parthasarathy S, Chi FW, Mertens JR, Weisner C. The role of continuing care in 9-year cost trajectories of patients with intakes into an outpatient alcohol and drug treatment program. Med Care. 2012;50(6):540-546. doi:10.1097/MLR.0b013e318245a66b 22584889 PMC3354333

[11] Mertens JR, Flisher AJ, Satre DD, Weisner CM. The role of medical conditions and primary care services in 5-year substance use outcomes among chemical dependency treatment patients. Drug Alcohol Depend. 2008;98(1-2):45-53. doi:10.1016/j.drugalcdep.2008.04.007 18571875 PMC2741640

[12] Saitz R, Horton NJ, Larson MJ, Winter M, Samet JH. Primary medical care and reductions in addiction severity: a prospective cohort study. Addiction. 2005;100(1):70-78. doi:10.1111/j.1360-0443.2005.00916.x 15598194

[13] American College of Emergency Physicians Public Health and Injury Prevention Committee. Stigma in the emergency department: an information paper. 2020. Accessed May 16, 2022. https://www.acep.org/globalassets/new-pdfs/information-and-resource-papers/stigma-in-the-emergency-department.pdf

[14] Matthews S, Dwyer R, Snoek A. Stigma and self-stigma in addiction. J Bioeth Inq. 2017;14(2):275-286. doi:10.1007/s11673-017-9784-y 28470503 PMC5527047

[15] Ray MK, Beach MC, Nicolaidis C, Choi D, Saha S, Korthuis PT. Patient and provider comfort discussing substance use. Fam Med. 2013;45(2):109-117. 23378078 PMC3608897

[16] Press KR, Zornberg GZ, Geller G, Carrese J, Fingerhood MI. What patients with addiction disorders need from their primary care physicians: a qualitative study. Subst Abus. 2016;37(2):349-355. doi:10.1080/08897077.2015.1080785 26360503

[17] McCarty D, Rieckmann T, Baker RL, McConnell KJ. The perceived impact of 42 CFR Part 2 on coordination and integration of care: a qualitative analysis. Psychiatr Serv. 2017;68(3):245-249. doi:10.1176/appi.ps.201600138 27799017 PMC5441679

[18] Weisner CM, Chi FW, Lu Y, . Examination of the effects of an intervention aiming to link patients receiving addiction treatment with health care: the LINKAGE clinical trial. JAMA Psychiatry. 2016;73(8):804-814. doi:10.1001/jamapsychiatry.2016.0970 27332703 PMC4972645

[19] Gordon NP. Similarity of adult Kaiser Permanente members to the adult population in Kaiser Permanente’s Northern California service area: comparisons based on the 2017/2018 cycle of the California Health Interview Survey. Kaiser Permanente Division of Research. November 8, 2020. Accessed March 22, 2022. https://divisionofresearch.kaiserpermanente.org/projects/memberhealthsurvey/SiteCollectionDocuments/compare_kp_ncal_chis2017-18.pdf

[20] Hibbard JH, Stockard J, Mahoney ER, Tusler M. Development of the Patient Activation Measure (PAM): conceptualizing and measuring activation in patients and consumers. Health Serv Res. 2004;39(4 Pt 1):1005-1026. doi:10.1111/j.1475-6773.2004.00269.x 15230939 PMC1361049

[21] Greene J, Hibbard JH, Sacks R, Overton V. When seeing the same physician, highly activated patients have better care experiences than less activated patients. Health Aff (Millwood). 2013;32(7):1299-1305. doi:10.1377/hlthaff.2012.1409 23836747

[22] Maeng DD, Martsolf GR, Scanlon DP, Christianson JB. Care coordination for the chronically ill: understanding the patient’s perspective. Health Serv Res. 2012;47(5):1960-1979. doi:10.1111/j.1475-6773.2012.01405.x 22985032 PMC3513613

[23] Alexander JA, Hearld LR, Mittler JN, Harvey J. Patient-physician role relationships and patient activation among individuals with chronic illness. Health Serv Res. 2012;47(3 Pt 1):1201-1223. doi:10.1111/j.1475-6773.2011.01354.x 22098418 PMC3423181

[24] Parchman ML, Zeber JE, Palmer RF. Participatory decision making, patient activation, medication adherence, and intermediate clinical outcomes in type 2 diabetes: a STARNet study. Ann Fam Med. 2010;8(5):410-417. doi:10.1370/afm.1161 20843882 PMC2939416

[25] Hochhalter AK, Song J, Rush J, Sklar L, Stevens A. Making the Most of Your Healthcare intervention for older adults with multiple chronic illnesses. Patient Educ Couns. 2010;81(2):207-213. doi:10.1016/j.pec.2010.01.018 20223617

[26] Deen D, Lu W-H, Rothstein D, Santana L, Gold MR. Asking questions: the effect of a brief intervention in community health centers on patient activation. Patient Educ Couns. 2011;84(2):257-260. doi:10.1016/j.pec.2010.07.026 20800414

[27] American Psychiatric Association. Diagnostic and Statistical Manual of Mental Disorders. 4th ed, text revision. American Psychiatric Association; 2000.

[28] Caetano R, Raspberry K. Drinking and *DSM-IV* alcohol and drug dependence among White and Mexican-American DUI offenders. J Stud Alcohol. 2000;61(3):420-426. doi:10.15288/jsa.2000.61.420 10807213

[29] Charlson ME, Charlson RE, Peterson JC, Marinopoulos SS, Briggs WM, Hollenberg JP. The Charlson Comorbidity Index is adapted to predict costs of chronic disease in primary care patients. J Clin Epidemiol. 2008;61(12):1234-1240. doi:10.1016/j.jclinepi.2008.01.006 18619805

[30] Charlson M, Wells MT, Ullman R, King F, Shmukler C. The Charlson Comorbidity Index can be used prospectively to identify patients who will incur high future costs. PLoS One. 2014;9(12):e112479. doi:10.1371/journal.pone.0112479 25469987 PMC4254512

[31] Johnston KJ, Allen L, Melanson TA, Pitts SR. A “patch” to the NYU emergency department visit algorithm. Health Serv Res. 2017;52(4):1264-1276. doi:10.1111/1475-6773.12638 28726238 PMC5517669

[32] Ballard DW, Price M, Fung V, . Validation of an algorithm for categorizing the severity of hospital emergency department visits. Med Care. 2010;48(1):58-63. doi:10.1097/MLR.0b013e3181bd49ad 19952803 PMC3881233

[33] Zou GY, Donner A. Extension of the modified Poisson regression model to prospective studies with correlated binary data. Stat Methods Med Res. 2013;22(6):661-670. doi:10.1177/0962280211427759 22072596

[34] White IR, Royston P, Wood AM. Multiple imputation using chained equations: Issues and guidance for practice. Stat Med. 2011;30(4):377-399. doi:10.1002/sim.4067 21225900

[35] Little RJ. Missing-data adjustments in large surveys. J Bus Econ Stat. 1988;6(3):287-296. doi:10.2307/1391878

[36] Des Jarlais DC, Lyles C, Crepaz N; TREND Group. Improving the reporting quality of nonrandomized evaluations of behavioral and public health interventions: the TREND statement. Am J Public Health. 2004;94(3):361-366. doi:10.2105/AJPH.94.3.361 14998794 PMC1448256

[37] Graetz I, Huang J, Muelly ER, Fireman B, Hsu J, Reed ME. Association of mobile patient portal access with diabetes medication adherence and glycemic levels among adults with diabetes. JAMA Netw Open. 2020;3(2):e1921429-e1921429. doi:10.1001/jamanetworkopen.2019.21429 32074289 PMC7646995

[38] Sarkar U, Lyles CR, Parker MM, . Use of the refill function through an online patient portal is associated with improved adherence to statins in an integrated health system. Med Care. 2014;52(3):194-201. doi:10.1097/MLR.0000000000000069 24374412 PMC4005993

[39] Harris LT, Koepsell TD, Haneuse SJ, Martin DP, Ralston JD. Glycemic control associated with secure patient-provider messaging within a shared electronic medical record: a longitudinal analysis. Diabetes Care. 2013;36(9):2726-2733. doi:10.2337/dc12-2003 23628618 PMC3747898

[40] Iturralde E, Slama N, Kline-Simon AH, Young-Wolff KC, Mordecai D, Sterling SA. Premature mortality associated with severe mental illness or substance use disorder in an integrated health care system. Gen Hosp Psychiatry. 2021;68:1-6. doi:10.1016/j.genhosppsych.2020.11.002 33227668

[41] Saitz R, Cheng DM, Winter M, . Chronic care management for dependence on alcohol and other drugs: the AHEAD randomized trial. JAMA. 2013;310(11):1156-1167. doi:10.1001/jama.2013.277609 24045740 PMC3902022

[42] Samet JH, Larson MJ, Horton NJ, Doyle K, Winter M, Saitz R. Linking alcohol- and drug-dependent adults to primary medical care: a randomized controlled trial of a multi-disciplinary health intervention in a detoxification unit. Addiction. 2003;98(4):509-516. doi:10.1046/j.1360-0443.2003.00328.x 12653820

[43] Blumenthal D, Tavenner M. The “meaningful use” regulation for electronic health records. N Engl J Med. 2010;363(6):501-504. doi:10.1056/NEJMp1006114 20647183

[44] Steinbrook R. Health care and the American Recovery and Reinvestment Act. N Engl J Med. 2009;360(11):1057-1060. doi:10.1056/NEJMp0900665 19224738

[45] Lyles CR, Nelson EC, Frampton S, Dykes PC, Cemballi AG, Sarkar U. Using electronic health record portals to improve patient engagement: research priorities and best practices. Ann Intern Med. 2020;172(11)(suppl):S123-S129. doi:10.7326/M19-0876 32479176 PMC7800164

