# Supplementary material for: Patterns of Health Care Use 5 Years After an Intervention Linking Patients in Addiction Treatment With a Primary Care Practitioner
Source: JAMA Netw Open. 2022 Nov 10;5(11):e2241338. doi: 10.1001/jamanetworkopen.2022.41338 (PMC9650610; doi:10.1001/jamanetworkopen.2022.41338)
Supplement: Supplement 1. — Trial Protocol [file jamanetwopen-e2241338-s001.pdf]

Continuing Review Submission Form - (Version 7.0)

1.0 Continuing Review Form Welcome to the new Continuing Review Form! Please make sure you 'Convert to the New Form Version' if prompted. NOTE: This form now features dynamic show/hide functionality: Questions will appear and disappear as you complete the form. The form now hides questions that are not relevant to your study. If the question numbers skip (e.g. 2.1, 2.4, 2.5, 2.8), it's because some questions are hidden. The form is functioning normally. For the best experience, please avoid using the Chrome browser. Other changes include: Fewer sections Shorter form for minimal risk research and studies not involving subject contact Smarter form includes only relevant attachment sections More intuitive and complete Subject Enrollment section Elimination of the AE Summary Log for non-reportable AEs and deaths

1.1 Principal Investigator:

Constance M Weisner DrPH

1.2 Study Title:

Continuing Care Following Drug Abuse Treatment: Linkage with Primary Care

1.3 Study Number:

10-01606

1.4 Lay Summary:

This component of a larger Center of Research Excellence Grant improves treatment for drug abuse by developing effective linkages between specialty drug treatment and primary health care. To implement the intervention we will use the setting of a large integrated managed care health plan and its drug treatment and primary care clinics in San Francisco Medical Center of Kaiser Permanente Northern California (KPNC). We will assign 700 drug treatment patients to the program's Usual Care or to Usual Care plus Continuing Care. We propose a quasi-experimental alternating off/on design to examine the outcomes and costs of adding to usual care three continuing care elements: a group session on patient activation targeted at overall health behaviors; a group session on selecting a primary care physician and communicating with him/her, and a linkage phone call with the patient, clinician, and primary care physician. Study participant follow-up includes interviews at 6, 12 and 24 months combined with analysis of medical records.

1.5 * NEW - Biospecimen Banks, Research Databases, and Recruitment Registries - Does this IRB approval ONLY cover activities such as biospecimen collection/banking, and/or collection of data in a research registry or recruitment database: (REQUIRED)

○ Yes ◉ No

1.6 * This is a: (REQUIRED)

○ Continuing Review Only—no changes from last approval
◉ Continuing Review and Minor Modification
○ Continuing Review and Major Modification

For studies with changes, please see our online guidance for the difference between 'Major' and 'Minor' modifications.

1.8 * Types of changes being made (check all that apply): (REQUIRED)

- ☐ Making changes to PI or personnel
- ☐ Adding a new funding source
- ☐ Adding new sites
- ☐ Increasing enrollment numbers
- ☐ Adding radiation exposure for the first time (imaging, radioactive contrast agent or ionizing radiation)
- ☑ Other changes including changes to recruitment, procedures, risks, etc.

If the other changes are related to information provided in the application and/or the consent form, please

make these changes and attach the revised form / documents.

1.9 * Describe each modification and provide a justification/rationale for each one: (REQUIRED)

NOTE: A scientific rationale MUST be provided for each proposed change. Submissions will be returned if they are not provided.

We would like to add a 5-year follow-up interview to the study. We are attaching a) a 5-year telephone follow-up questionnaire which is identical to the approved questionnaire used at 2-years; b) a verbal consent script, to be used when we re-contact participants. Please note, that at the end of the 2-year telephone interview we obtained permission to contact them in the future to see if they would be interested in participating in another interview, if we were able to obtain funding.

We want to examine longer term patient activation and engagement, health, mental health, and substance use outcomes in our study population.

1.10 * Are any of these changes being made as a result of an adverse event report (AER), protocol violation or incident report, or publication of a new Investigator's Brochure (IB) or other safety data: (REQUIRED)

○ Yes ◉ No

1.12 * The modifications require changes to: (REQUIRED)

☑ Study Application
☑ Consent Documents
☑ Other Study Documents
☐ None

1.13 Check here if this is modification includes adding the use of CTSI Clinical Research Services or Centers for the first time:

○ Adding CRS

1.14 * Are there any changes in any financial interests related to this study or in any conflicts of interest of the PI or any other investigator: (REQUIRED)

○ Yes ◉ No

1.15 Expiration Date: Hint: Click 'Refresh Constant Fields' to update the expiration date if this is a copied form.

06/06/2017

* **Has your study expired:** (REQUIRED)

○ Yes ◉ No

1.16 Outstanding Stipulations:

No Stipulation is outstanding.

2.0 Study Status for Research Involving Subject Contact and Repositories

2.1 * Enrollment Status: (REQUIRED)

○ No subjects have EVER been enrolled here (or at any other sites if UCSF is the Coordinating Center)
○ We are continuing to enroll subjects
○ Some subjects have been enrolled but we are not actively recruiting
◉ All subjects have been enrolled and study is now closed to accrual

Do not submit consent forms for studies closed to accrual. Submit a modification if you need to resume recruitment or revise a consent form.

2.2 * Study Activity Status: (REQUIRED)

○ Study activities have not yet commenced
○ Study in progress and subjects are currently participating in study procedures, interventions, and/or research activities (some subjects may be in follow up)
○ Study intervention is complete for all subjects but there is ongoing research-related follow-up contact with participants via questionnaires, phones calls, interviews, or mailings.
○ Study procedures are complete for all subjects but ongoing medical record review/biological specimen analysis continues (no subject contact)
◉ Data analysis only - study is complete and the only activities are data analysis and/or manuscript preparation

* Have there been any reportable problems (adverse events, protocol violations, incidents or complaints) since the last review: (REQUIRED)

○ Yes ◉ No

3.0 Revisions to the Application Form

3.1 * Click the bar below to make revisions to the application form: (REQUIRED) (Note: you are seeing this section because you either indicated that there are changes that affect the application or there are personnel changes that need to be made in the application.)

Version	Title
1.10	Study Application (Version 1.10) - Attached)

4.0 Attach NEW or REVISED Other Study Documents

4.1 Changes to approved documents should be attached as 'Revisions' of the previous version. Your approval may be delayed if they are uploaded as 'New' documents.

* Attach the documents following these instructions: (REQUIRED) Click the Select or Revise Existing button. Click Upload the Revised Document and select the revised document from your computer. *If you need to download the current version of the document from iRIS first,* click Download Document for Editing and then Upload the Revised Document after you've updated the document. Save your work. **New Documents:** Click on the Add Document button and upload your new document. **Approved Documents – No Changes: Do not submit any unchanged already-approved documents**.
NOTE: Please make sure that any tracked changes have been accepted for both consent forms and study documents. If tracked changes are submitted, they will show in the stamped PDF and you will have to submit a modification to get clean documents stamped.

Note: Non-reportable events should no longer be submitted at the time of Continuing Review on the AE Summary Log.

Version	Title	Category	Last Modified By	Date Last Modified
1.0	thank you letter.gc	Other	Constance M. Weisner	04/26/2017 01:45:47 PM
1.0	linkage 5 year quex	Questionnaire	Constance M. Weisner	04/26/2017 01:45:47 PM
1.0	linkage 5-year verbal consent script	Other	Constance M. Weisner	04/26/2017 01:45:47 PM

Study Application (Version 1.10)

1.0 General Information
* Enter the full title of your study:
Continuing Care Following Drug Abuse Treatment: Linkage with Primary Care
* Enter the study number or study alias
Continuing Care Linkage
* This field allows you to enter an abbreviated version of the Study Title to quickly identify this study.
2.0 Add Department(s)
2.1 List departments and/or research programs associated with this study

Primary Dept?	Department Name
◉	UCSF - 133144 - M_Psych-LPPI-Core-General

3.0 Assign key study personnel(KSP) access to the study
3.1 * Please add a Principal Investigator for the study:
Constance M Weisner DrPH<br><br>Select if applicable<br><br>☐Department Chair ☐Resident ☐Fellow<br><br>If the Principal Investigator is a Student, Resident, or Fellow, the name of the Faculty Advisor must be supplied below.
3.2 If applicable, please select the Research Staff personnel:
A) Additional Investigators<br><br>Paul Barnett PhD<br><br>Other Investigator<br><br>Kevin Delucchi PhD<br><br>Other Investigator<br><br>Joseph Guydish PhD<br><br>Other Investigator<br><br>B) Research Support Staff
3.3 Please add a Study Contact:
Constance M Weisner DrPH<br><br>Michael W Worden<br><br>The Study Contact(s) will receive all important system notifications along with the Principal Investigator. (e.g. The project contact(s) are typically either the Study Coordinator or the Principal Investigator themselves).
3.4 If applicable, please add a Faculty Advisor/Mentor:

3.5 If applicable, please select the Designated Department Approval(s):

Add the name of the individual authorized to approve and sign off on this protocol from your Department (e.g. the Department Chair or Dean).

4.0 Qualifications of Key Study Personnel

4.1 November, 2015 - NEW Definition of Key Study Personnel and CITI Training Requirements: UCSF Key Study Personnel include the Principal Investigator, other investigators and research personnel who are directly involved in conducting research with study participants or who are directly involved in using study participants' identifiable private information during the course of the research. Key Personnel also include faculty mentors/advisors who provide direct oversight to Postdoctoral Fellows, Residents and Clinical Fellows serving as PI on the IRB application. The IRB requires that all Key Study Personnel complete Human Subjects Protection Training through CITI prior to approval of a new study, or a modification in which KSP are being added. More information on the CITI training requirement can be found on our website. List the study responsibilities and qualifications of any individuals who qualify as Key Study Personnel (KSP) at UCSF and affiliated sites ONLY by clicking the "Add a new row" button. This information is required and your application will be considered incomplete without it.

KSP Name	Description of Study Responsibilities	Qualifications
Dr. Weisner, Constance M DrPH	Principal Investigator: Dr. Weisner will advise on study design, data analysis, and the dissemination of the study findings to the professional community through presentations, manuscripts, and working with state and national workgroups as discussed in the Dissemination section of the proposal.<br><br>Dr. Weisner will advise study staff on finalizing design issues and instrument development, and analysis and manuscript production throughout the study period.<br><br>Dr. Weisner will participate in dissemination efforts within the health plan and larger medical community, and in developing manuscripts for publication.	Constance Weisner, DrPH, MSW, Principal Investigator of the Subcontract, is a Professor in the Department of Psychiatry, University of California, San Francisco. She also has a joint position with the Kaiser Division of Research, through a Memorandum of Understanding between UCSF and The Permanente Medical Group, Inc., Division of Research (TPMG/DOR) on behalf of Kaiser Foundation Health Plan, Kaiser Foundation Hospitals and The Permanente Medical Group. The agreement between the two organizations allows Weisner to lead the mental health/chemical dependency clinical outcomes and health services research within the Kaiser Permanente health care system. She has a research office located at the Division of Research in close proximity to Dr. Mertens' office and those of the other members of the research group. Weisner directs a research program at Kaiser Permanente's Division of Research addressing access, outcomes, and cost effectiveness of alcohol and drug treatment. She is the Principal Investigator of NIAAA, NIDA, and RWJF research grants that study alcohol epidemiology and services in primary care, disease management, and specialty alcohol and drug treatment.
Dr. Guydish, Joseph PhD	Dr. Guydish will provide policy analysis.	

		Dr. Joseph Guydish, is a well-established, well-published investigator who was a Center Co-Director and is currently Professor-in Residence of Medicine and Health Policy at UCSF. He has a Ph.D. in clinical psychology and an M.P.H. in epidemiology. His publication record includes work on smoking and drug abuse, and he is currently PI of an R01 from NIDA on organizational change and nicotine dependence treatment, conducted through the NIDA CTN, and he is Co-Director of the California-Arizona node of the CTN.
Dr. Delucchi, Kevin PhD	Dr. DeLucchi will provide expertise in data analysis.	Dr. Kevin Delucchi received his 1986 Ph.D. from the University of California, Berkeley in quantitative methods. He has a strong track record of publication, and publishes on both quantitative methodology and the application of these methods to drug abuse. He is PI on a grant from the National Institute on Alcohol Abuse and Alcoholism.
Dr. Barnett, Paul PhD	Dr. Barnett will provide expertise in health economics.	Dr. Paul Barnett is a 1993 Ph.D. in economics from the University of California, Berkeley. He is currently Director of Health Economics at the Palo Alto VA Research Center.

5.0 Initial Screening Questions - Updated 9/13 (Note: You must answer every question on this page to proceed). If you are converting to the new form, check questions 5.4, 5.6, 5.7, 5.8 and 5.10 before saving and continuing to the next section.

5.1 * Application type:

- (•) Full Committee
- ( ) Expedited
- ( ) Exempt

5.2 * Risk level (Help Text updated 9/13):

- ( ) Minimal risk
- (•) Greater than minimal risk

5.3 * Subject contact:

- (•) Yes (including phone, email or web contact)
- ( ) No (limited to medical records review, biological specimen analysis, and/or data analysis)

5.4 * Funding (past or present):

- (•) Funded or will be funded (external sponsor, gift, program or specific internal or departmental funds)
- ( ) Unfunded (no specific funds earmarked for this project)
- ( ) Unfunded student project

5.5 * The Principal Investigator and/or one or more of the key study personnel has financial interests related to this study:

( ) Yes (•) No

If **Yes**, the Conflict of Interest Advisory Committee (COIAC) office may contact you for additional information.

5.6 * This is an investigator-initiated study:

◉ Yes ○ No

5.7 * This study ONLY involves retrospective records review and/or identifiable biospecimen analysis:

○ Yes ◉ No

5.8 * This is a clinical trial:

○ Yes ◉ No

Clinical Trial Registration

"NCT" number for this trial:

5.9 * This is a multicenter study:

◉ Yes ○ No

5.10 * This application involves the study of unapproved or approved drugs, devices, biologics or in vitro diagnostics:

○ Yes ◉ No

5.11 * This application involves a Humanitarian Use Device:

◉ No
○ Yes, and it includes a research component
○ Yes, and it involves clinical care ONLY

5.12 * This study involves human stem cells (including iPS cells and adult stem cells), gametes or embryos:

◉ No
○ Yes, and requires CHR and GESCR review
○ Yes, and requires GESCR review, but NOT CHR review

5.13 * This is a CIRB study (e.g. the NCI CIRB will be the IRB of record):

○ Yes ◉ No

5.14 * This application includes a request to rely on another IRB (other than NCI CIRB):

○ Yes ◉ No

Note: If this request is approved, the CHR will **NOT** review and approve this study. Another institution will be the IRB of record.

6.0 Funding

6.1 Identify all sponsors and provide the funding details. If funding comes from a Subcontract, please list only the Prime Sponsor: Note: we require only a P Number OR an A Number for funding coming through UCSF. Please avoid these common errors in funding documentation: DO NOT add the A Number if a P Number was already provided OR update the A Number field when a new funding cycle begins. The IRB does NOT use this information or want these changes made. DO NOT add a grant continuation as a new funding source.

External Sponsor:

	Sponsor Name	Sponsor Type	Funding Through	Contract Type:	UCSF RAS "P number" or ePropos	UCSF RAS System Award Number

					al number	("A" + 6 digits)
—	NIH Natl Institute on Drug Abuse	01	UCSF	Grant	P0030871	A115760

Sponsor Name:	NIH Natl Institute on Drug Abuse
Sponsor Type:	01
Sponsor Role	Funding
Grant/Contract Number	
Awardee Institution	UCSF
Is Institution the Primary Grant Holder:	Yes
Contract Type:	Grant
UCSF RAS "P number" or eProposal number	P0030871
UCSF RAS System Award Number ("A" + 6 digits)	A115760
Grant Number for Studies Not Funded thru the institution:	
Grant Title:	
Award Recipient:<br>If Award Recipient is not the same as identified on the study.	
Explain Any Significant Discrepancy:	

Gift, Program, or Internal Funding (check all that apply):

☐ Funded by gift (specify source below)
☐ Funded by UCSF or UC-wide program (specify source below)
☐ Specific departmental funding (specify source below, if applicable)

List the gift, program, or departmental funding source:

6.2 If you tried to add a sponsor in the question above and it was not in the list, follow these steps: If funding has already been awarded or the contract is being processed by the Office of Sponsored Research (OSR) or Industry Contracts Division (ICD), your sponsor is already in the system and the project has an eProposal Proposal or Award number. Check with your department's OSR Staff or ICD Officer to ask how the sponsor is listed in the UC sponsor list and what the Proposal or Award number is. Click here to find your OSR staff and here to find your ICD staff. If your sponsor is not yet in the list, enter it in the box below.

○ Sponsor not in list

Only if your sponsor is not yet in the list, type the sponsor's name:

If the funding is administered by the UCSF Office of Sponsored Research, your study will not receive CHR approval until the sponsor and funding details have been added to your application.

6.3 * This study is currently supported in whole or in part by Federal funding OR has received ANY Federal funding in the past (Help Text updated 9/13):

◉ Yes ○ No

If **yes**, indicate which portion of your grant you will be attaching:

☑ The Research Plan, including the Human Subjects Section of your NIH grant or subcontract
☐ For other federal proposals (contracts or grants), the section of the proposal describing human subjects work
☐ The section of your progress report if it provides the most current information about your human subjects work
☐ The grant is not attached. The study is funded by an award that does not describe specific plans for human subjects, such as career development awards (K awards), cooperative agreements, program projects, and training grants (T32 awards) OR UCSF (or the affiliate institution) is not the prime recipient of the award

7.0 Sites

7.1 Institutions (check all that apply):

- [x] UCSF
- [ ] China Basin
- [ ] Helen Diller Family Comprehensive Cancer Center
- [ ] Mission Bay
- [ ] Mount Zion
- [ ] San Francisco General Hospital (SFGH)
- [ ] SF VA Medical Center (SF VAMC)
- [ ] Blood Centers of the Pacific (BCP)
- [ ] Blood Systems Research Institute (BSRI)
- [ ] Fresno (Community Medical Center)
- [ ] Gallo
- [ ] Gladstone
- [ ] Institute on Aging (IOA)
- [ ] Jewish Home
- [ ] SF Dept of Public Health (DPH)

7.2 Check all the other types of sites not affiliated with UCSF with which you are cooperating or collaborating on this project (Help Text updated 9/13):

- [ ] Other UC Campus
- [x] Other institution
- [ ] Other community-based site
- [ ] Foreign Country

List the foreign country/ies:

7.3 Check any research programs this study is associated with:

- [ ] Cancer Center
- [ ] Center for AIDS Prevention Sciences (CAPS)
- [ ] Global Health Sciences
- [ ] Immune Tolerance Network (ITN)
- [ ] Neurosciences Clinical Research Unit (NCRU)
- [ ] Osher Center
- [ ] Positive Health Program

8.0 Studies Involving Other Sites

8.1 UCSF is the coordinating center:

(●) Yes ( ) No

If **Yes**, describe the plan for communicating safety updates, interim results, and other information that may impact risks to the subject or others among sites:

Through a Memorandum of Understanding between the University of California, San Francisco (UCSF), and The Permanente Medical Group, Inc., Division of Research (TPMG/DOR) on behalf of Kaiser Foundation Health Plan, Kaiser Foundation Hospitals and The Permanente Medical Group, Dr. Weisner has a joint position with UCSF, Department of Psychiatry and Kaiser DOR. In accordance with policies of the UCSF Regents, all research projects of faculty serving as Principal Investigator are submitted through the University. However, Dr. Weisner is an Adjunct Investigator at the Division of Research, her research office is at Kaiser's Division of Research and all the work of her studies is conducted at Kaiser. Thus, she will work

closely with the research staff at Kaiser's Division of Research. Dr. Weisner has a long history of working with this same research group in both sites and has well-established protocols for communicating pertinent information between sites. In her role working so closely with both the UCSF and Kaiser Division of Research study teams, she will be responsible for communicating safety updates, interim results, and other information that may impact risks to the subjects or others among sites.

If
Yes
, describe the plan for sharing modification(s) to the protocol or consent document(s) among sites:

Through a Memorandum of Understanding between the University of California, San Francisco (UCSF), and The Permanente Medical Group, Inc., Division of Research (TPMG/DOR) on behalf of Kaiser Foundation Health Plan, Kaiser Foundation Hospitals and The Permanente Medical Group, Dr. Weisner has a joint position with UCSF, Department of Psychiatry and Kaiser DOR. In accordance with policies of the UCSF Regents, all research projects of faculty serving as Principal Investigator are submitted through the University. However, Dr. Weisner is an Adjunct Investigator at the Division of Research, her research office is at Kaiser's Division of Research and all the work of her studies is conducted at Kaiser. Thus, she will work closely with the research staff at Kaiser's Division of Research. Dr. Weisner has a long history of working with this same research group in both sites and has well-established protocols for communicating pertinent information between sites. In her role working so closely with both the UCSF and Kaiser Division of Research study teams, she will be responsible for sharing modification(s) to the procotol or consent document(s) among sites.

8.2 Check any other UC campuses with which you are collaborating on this research study:

- ☐ UC Berkeley
- ☐ UC Davis
- ☐ Lawrence Berkeley National Laboratory (LBNL)
- ☐ UC Irvine
- ☐ UC Los Angeles
- ☐ UC Merced
- ☐ UC Riverside
- ☐ UC San Diego
- ☐ UC Santa Barbara
- ☐ UC Santa Cruz

8.3 Are the above UC campuses requesting to rely on UCSF's IRB (check all that apply):

- ☐ Yes (Submit a reliance request through the UC IRB Reliance Registry)
- ☐ No (Complete IRB Approval Certification section)

9.0 Outside Site Information

9.1 Outside Site Information

Click "Add a new row" to enter information for a site. Click it again to add a second site again to add a third site, a fourth site, etc.

1.0 Outside Site Information

1.1 Non-UCSF affiliated site information:

Site name:

Kaiser Permanente Division of Research, Oakland, CA

Contact name:

Stacy Sterling, MSW

Email:

stacy.a.sterling@kp.org

Phone:

510-891-3614

1.2 For Federally-funded studies only, corresponding FWA#:

FWA00002344

1.3 * The research at this site will be reviewed by:

- (●) The non-affiliated site's IRB or a private IRB
- ( ) The non-affiliated site is requesting UCSF to be the IRB of record for this study
- ( ) The non-affiliated site is not engaged in the human subjects research and has provided a letter of support

If the other site's IRB approval letter is available now, attach it to the application. If the IRB approval letter is not yet available, submit it once you receive it. Or, if the other site is not engaged in human subjects research, attach the letter of support to your application.

10.0 Study Design

10.1 * Study design (Help Text updated 9/13):

Study Design This is a longitudinal two-arm quasi-experimental 2-month alternating off/on design over a 20 month period in which, after a random start, the Continuing Care condition alternately is added to Usual Care in the clinic and then removed. We compare Usual Care to Usual Care plus Continuing Care. Follow-up interviews will be conducted at 6, 12, 24 and at 5 years. The Continuing Care intervention includes Usual Care plus sessions on activation regarding overall health behaviors and on selecting a PC physician and communicating with him/her, and 2) a linkage phone call with the patient, clinician, and Primary Care physician. The linkage conference call is set up as a phone consult appointment in the physician's and clinician's schedules in the EMR. The design also includes a 1-hour informational session for PC physicians during a lunch staff meeting to inform them of the linkage, information on where to find guidelines in the EMR, and also an EMR alert reminder to the PC Physician before the patient's visit. The intervention aims to place drug abuse problems in the context of overall health and health care and to activate patients to increase involvement in their own health care.

10.2 If this is a clinical trial, check the applicable phase(s) (Help Text updated 9/13):

- [ ] Phase I
- [ ] Phase II
- [ ] Phase III
- [ ] Phase IV

11.0 Scientific Considerations

11.1 Hypothesis (Help Text updated 9/13):

This study has a hypothesis:

(•) Yes ( ) No

If yes, state the hypothesis or hypotheses:

Primary Hypotheses
1. We hypothesize that patients in the Continuing Care condition will have higher rates of PC utilization, including preventive visits, at 6, 12 and 24 months, and at 5 years. Continuing Care patients will have higher patient activation scores. Patient activation will mediate the relationship between Continuing Care and PC visits.
2. We hypothesize that at 6, 12 and 24 months and 5 years, patients in the Continuing Care condition will have higher rates of drug and alcohol abstinence, including tobacco abstinence, than those in Usual Care. PC visits will mediate the relationship between Continuing Care and abstinence. Effects of Continuing Care on abstinence rates will be greater for individuals with substance abuse-related medical conditions (SAMCs).
3. We hypothesize that at 6, 12, and 24 months and 5 years patients in the Continuing Care condition will have better health and psychosocial outcomes (e.g., health behaviors, physical and mental health status).
4. We hypothesize that at 6, 12 and 24 months and 5 years, PC costs will be higher in the Continuing Care condition because of more PC visits (Aim A.2.1.), but emergency room (ER) and inpatient costs will be lower. By 5 years, total costs (outpatient, pharmacy, psychiatry, drug treatment, ER, inpatient, labs, and outside claims) will be lower for the Continuing Care condition. We will obtain the cost-effectiveness (CE) ratio and test the hypothesis that the intervention is cost-effective for various threshold values of the CE ratio.

For each hypothesis, we will examine whether gender, age, and race/ethnicity make a difference.

11.2 * List the specific aims:

1. To Compare Primary Care Utilization in the Continuing Care Condition with Usual Care.
2. To Compare Substance Use Outcomes Between the Continuing Care Condition and Usual Care.
3. To Compare Health and Other Psychosocial Outcomes Between the Continuing Care Condition and Usual Care.
4. To Compare the Cost of the Continuing Care Condition with Usual Care and Assess Cost Effectiveness.
5. To analyze a health risk assessment that incorporates assessments of stage of change, including examining:
    a) The prevalence of multiple risk behaviors; b) Readiness to change multiple risk; c) The association between multiple risk behaviors and health-related quality of life; d) Covariation in changes in risk behaviors over time -- specifically, to examine how changes in tobacco, alcohol, and other drug use are related to changes in additional risk behaviors, including injection drug use and sexual risk behaviors, and e) The medical costs associated with multiple risks and changes over 18-months time. We will also explore correlates of multiple risks and stage of change with demographics (ethnicity, age, gender, education, socioeconomic status), HIV/AIDS status, substance use severity, and psychiatric comorbidity. Additionally, the prevalence of multiple risk behaviors and stage of change among individuals in drug abuse treatment will be compared to normative and comparative data from three large samples.

Secondary Aims

Because of the physician-patient interaction, we expect that Continuing Care patients will be more likely to be monitored for substance use (measured by self-report and electronic medical record (EMR) in PC over the 24 months, and over 5 years, than those in Usual Care.

Because of PC visits and monitoring, we expect that Continuing Care patients will have more drug treatment visits (consults and/or readmissions) following index treatment than those in Usual Care.

11.3 Statistical analysis:

Data Analyses
Analyses will be conducted under the direction of the Statistics and Health Economics Core (PI, Dr. Delucchi) in close collaboration with the DOR research team led by Dr. Weisner. The aims are met by comparing the two treatment conditions on relevant outcome measures of PC utilization rates, drug and alcohol abstinence rates, patient activation scores, health and psychological status, PC and treatment visits and cost. As this is a longitudinal two-arm, quasi-randomized trial, comparisons between conditions are straightforward. The most statistically appropriate method is used to test each hypothesis. While in theory nothing more than a simple two-

group test is required, to control for person-to-person variation and the dependence created by measuring each person at over several assessments, a model which includes covariates is estimated for each outcome. Each model includes a term (e.g., treatment versus control) which directly tests the hypothesis while controlling for the other variables. We will use the mediators (patient activation and primary care visits) as time-varying measures. Cohort effects will also be tested. We will include demographic characteristics as main predictors in each Aim, and also interact them with the intervention arm. If significant, we will conduct further analysis stratified by the variables of interest.
Cost analyses specific to this component are as follows: Aim 4 hypothesizes that PC costs will be higher in the Continuing Care condition because of more PC visits but emergency room and inpatient costs will be lower. By 24 months, total costs will be lower for those in the Continuing Care condition. Health care cost is not a normally distributed variable; some observations have zero values and the distribution is often skewed. We will make logarithmic or other transformations of cost as appropriate based on preliminary analyses of cost data. In the analyses of cost, we will take the perspective of society. Treatment groups will be compared, controlling for baseline cost using either log and log-link generalized linear models, choosing between them using the method described by Manning et al. Trial participants will be enrolled over time and followed to the end of the study, resulting in different observation periods. This results in different observation periods and represents a right-censoring of cost data. Differences in cumulative cost will be tested while considering this right censoring employing alternate analytical procedures: the Kaplan-Meier product limit estimator and the weighted cost method.
We will obtain the cost-effectiveness (CE) ratio for the two interventions and test the hypothesis that the intervention is cost-effective for various threshold values of the CE ratio. The incremental cost-effectiveness ratio will be expressed in dollars per Quality Adjusted Life Year. Outcomes will be measured using the Health Utilities Index. We will use a bootstrap approach to estimate the confidence region surrounding cost-effectiveness. We will estimate statistical significance of the intervention over the range of critical cost-effectiveness ratios, a graph that has been named the acceptability curve. We will test the sensitivity of the findings to analytic assumptions using a probabilistic sensitivity analysis. This Monte Carlo method incorporates uncertainty about all parameters simultaneously. The distribution of each parameter will be represented by our estimate of the 95% confidence interval for its value. We will then find the variance associated with the model by repeated random samples of parameters from these distributions.

11.4 If this study has undergone scientific or scholarly review, please indicate which entity performed the review:

☐ Cancer Center Protocol Review Committee (PRC) (Full approval is required prior to final CHR approval for cancer-related protocols.)
☐ CTSI Clinical Research Center (CRC) advisory committee
☐ Departmental scientific review
☑ Other:

Specify
Other
:

NIH National Institute on Drug Abuse (NIDA) Scientific Review Committee

12.0 Background

12.1 Background:

BACKGROUND AND SIGNIFICANCE
Substance Use Disorders as Chronic Conditions. Like asthma, hypertension and diabetes,[1] the onset and course of substance use disorders are influenced by genetic heritability as well as by behavior and personal choice.[2-4] They are better managed with ongoing monitoring and extended services than by an acute treatment approach.[5] Improvement is characterized by patterns of abstinence, relapse and treatment readmission, often over many years.[6-8]
Chronic Care Model in Health Care. Continuing care models have been refined for chronic illnesses such as diabetes,[9, 10] and include collaborative PC and specialty care management that supports treatment and self-care. A ground-breaking Institute of Medicine report focusing on general health care,[11] and a second focusing on addiction and mental health[12] have emphasized this patient-centered approach. They call for patients to take initiative in their health care, with “the patient as the source of control.” The Institute of Medicine report argued that this approach reduces the stigma of substance use disorders within health care by maximizing the patients’ ability to manage their own care, improving clinician attitudes, and identifying and eliminating discriminatory policies.[12] Research, including in the health plan studied here, has examined patient activation as a key factor in a patient-centered approach for chronic conditions of asthma, pain,

coronary artery disease, diabetes, and heart failure, and found that higher patient activation is related to better outcomes.[13, 14] Patient activation involves helping patients increase their skills to manage medical conditions, by providing education about their conditions, learning how their health care is related to improvement and what is needed to manage their condition, and how to use health care.[10, 11, 13, 15, 16] This is increasingly used in chronic care and disease management. Wagner has been at the forefront in laying out the principles for chronic care, including those that guided the Institute of Medicine reports, arguing that principles of successful chronic illness care include ready access to expertise and supportive information, through mechanisms such as specialty consultation, provider partnerships and communication between PC and specialty care, a computer reminder system, and a patient education activity.[17, p.514)] The most current models of systems of care include dual management across specialty services and PC with the patient involved in the process, and the use of electronic medical records and other technology for education and communication. [11] Components also include decision support and consultation between specialty and PC, "practice redesign" (e.g., electronic appointments, sharing roles, triggers/reminders to clinicians and patients), "patient education" (e.g., self-management, patient participation, how to use the system, availability of information on health and illness), "expert system" (e.g., decision support or available consultation), and "information" (e.g., reminders).[10, 11, 18] These are incorporated in our intervention.

Chronic Care Model in the Drug Abuse Field. Continuing care has similarities to "aftercare," "step-down care," and "stepped care" in drug treatment, which are associated with better outcomes.[19-28] Modalities have included telephone support, case management and web-based interventions. For example, in a step-down model, McKay found that for cocaine- and alcohol-dependent patients who had completed intensive outpatient treatment, weekly telephone-based monitoring and brief counseling contacts combined with weekly supportive group sessions in the first 4 weeks was associated with higher abstinence rates at two years, compared with standard treatment.[23] Another excellent example comes from Recovery Management Check-ups (RMC) after drug treatment (receiving quarterly management checkups for two years by research staff, including assessment, motivational interviewing, and linkage to drug treatment readmission). RMCs also were related to better drug use outcome at two years.[29] One concern by many of these authors is that services are often only applied to individuals who have completed drug treatment. We build on this prior work, pointing out that our intervention developing a linkage with PC can be implemented early in treatment and be applied to those who do not complete treatment. For those who do complete drug treatment, our intervention can be overlaid on any of these extended care interventions, which could, in fact, reinforce the PC linkage. Aftercare is clearly related to positive outcomes, but its reach is limited -- *no matter how long aftercare continues, it ends at some point and patients become disconnected from the clinic site, specialty care providers, and other resources where they received services.* On the other hand, a patient throughout life can use PC as an anchoring 'medical home' for identifying problems and needs for ongoing care, including medical management, encouragement supporting recovery, or referral back to drug treatment. Several studies now have found that integrated PC and drug treatment are effective during treatment.[30-36] One study has examined a linkage intervention post-detox[37] (see below), and others have examined the role of PC observationally.[33, 38, 39] We have drawn from this literature to develop our continuing care intervention.

Primary Care-Linkage Model of Continuing Care for Drug Abuse. Of critical importance to the development of our intervention was Samet and colleagues' randomized linkage study.[37] An uninsured sample undergoing inpatient detoxification from alcohol, heroin and cocaine was linked to a PC physician; the likelihood that they would access medical services within the following 12 months increased.[37] The intervention included a medical and social work team working within a detox facility to facilitate a linkage between the patient and a PC provider demographically similar to the patient. It included making an appointment and sending a letter from the social worker to the PC provider. In addition to the short-term findings of more PC visits for those randomized to the linking intervention, an on-going observational study found better substance use outcomes for those who had two or more PC visits.[40] Dr. Samet, the PI of that study, has advised us on the development of our intervention and serves as a Consultant on this study, better enabling us to apply what that study learned about how to facilitate the linkage between patient and provider, and to activate the patient. We expand from a detox population used in that study to an outpatient drug treatment program as our study population. We extend the intervention by providing more emphasis on patient activation, adding a conference call (booked in the PC physician's schedule) facilitated by a drug treatment clinician to begin the conversation between the PC physician and the patient, as well as a brief orientation to health plan PC physicians.

Patient-Centered Care and Patient Activation. Underlying the quality of care approach in the Institute of Medicine reports is the "patient-centered" concept of patient activation and empowerment. Similarly, studies of chronic care have identified self-efficacy and self-management as critical concepts related to patient activation and self-determination.[10] They focus on core skills: "problem solving, decision making, resource

utilization, formation of an effective patient-provider relationship, and taking action."[11, 41] These skills enable patients to perform activation tasks such as "communicating effectively with health care providers and practicing health-related problem solving and decision-making." These are part of our intervention, and ingredients of a chronic care model.[11, 42] Building on the patient activation literature, our intervention includes Usual Care plus two patient activation groups that focus on activation regarding overall health, selecting a PC physician and communicating with him/her, and a linkage phone call between the patient, therapist, and PC physician. The goal is to address drug use problems in the context of overall health care and to activate patients to be fully involved. Our model also provides an informational lunch session for PC physicians on the linkage, where to find guidelines in the electronic medical record (EMR), and an email reminder before the patient's visit (see Methods)

<u>Role of Medical Comorbidities and PC Services In Outcomes.</u> Including PC services in our continuing care model could have broad clinical significance, since many drug abuse patients have medical problems[43-46] that can trigger relapse. For patients with a chronic disease, treatment of other health conditions is often overlooked,[47] and this may also occur for patients with drug abuse. Drug abuse disorders are often viewed as conditions entirely separate from general medical care; they can become patients' entire medical identity and the focus of their health care.[12] Understanding co-occurring drug abuse and medical problems, treatment options, and service integration is crucial to formulating successful health policy.[12, 48, 49] Our study will help to address these issues, particularly development of service models for patients with complex and multiple medical conditions.

This study is unique in its focus on the larger health context, its relationship to outcome, and the potential benefit of health policy that includes PC in a chronic care model. Many medical problems primarily treated in PC clinics are related to drug use: hypertension, diabetes, HIV, hepatitis, liver dysfunction, cirrhosis, anemia, cardiovascular diseases, central nervous system effects, neurological dysfunction, pulmonary complications,[43, 45, 46, 50-52] alcoholic pancreatitis, endocrine functioning, reproductive capacity, and effects on the immune sys-tem.[43, 46, 53, 54] Our studies in this health plan[44] are consistent with these findings on medical conditions,[55-61] and our delineation of substance abuse-related medical conditions (SAMC) have been a large contribution to understanding these relationships.[30, 39]

PC services are predictive of better drug use outcomes for those with health problems,[40] particularly those related to and exacerbated by drug use.[30, 39] Yet, medical and drug treatment services are rarely coordinated.[62-68] Moreover, studies have found beneficial effects of PC when integrated with treatment.[30, 33] PC providers are thus well-placed to provide ongoing services in a continuing care model. Thus, our study responds to Saitz's challenge: "How to make PC accessible and actually used by adults with alcohol and other drug dependence."[40] If our study finds that PC services delivered after drug treatment result in improved recovery rates, it becomes incumbent to improve PC service access and to develop improved systems integration.

<u>Tobacco Dependence, Drug Treatment, and Linkages with PC.</u> Smoking prevalence is high in populations with other drug problems, while quit rates are low.[69-72] On the whole, a traditional reluctance to treat tobacco addiction in drug treatment persists; a recent national study of adult outpatient treatment found that 41% offered smoking cessation services.[73] Several reasons may account for this reluctance: tobacco use is seen as less harmful than other drug use, and therefore a lower priority to treat;[74] staff may smoke themselves;[74, 75] staff concern that smoking cessation may jeopardize recovery;[74, 76] lack of resources,[73] and a lack of awareness that patients often want to stop smoking.[74, 77] However, research shows that smoking cessation efforts in drug treatment do not hinder recovery, and some studies have found an association with improved drug outcomes.[78-82] In contrast, continued tobacco use puts these patients at higher risk of mortality and tobacco-related comorbidities.[83, 84] The proposed study offers the opportunity for the PC setting to continue to monitor tobacco use, and is likely a substance that is more comfortable to discuss with a PC physician; it may, in fact, become an entry into discussions about other types of drug use.

<u>The Role of Drug Treatment and Readmissions in Predicting Outcomes.</u> Literature on treatment careers has influenced our hypotheses that drug treatment is related to long-term outcomes, particularly when an episode is reinforced with supporting interventions or further treatment is provided.[8, 85, 86] We would expect PC services to provide services for comorbidities (e.g., chronic pain or hypertension) and also note potential relapse. This is partly based on findings that drug treatment patients with medical and psychiatric problems have higher odds of readmission.[55, 87-91] Our study further develops this approach, expanding access to resources to support recovery after initial drug treatment.

<u>The Role of Patient Characteristics in Continuing Care.</u> The literature strongly argues that patient characteristics are important in evaluating an intervention. Patient activation can vary by gender, race/ethnicity, and socio-economic status (SES). Because research focused directly on "activation" and demographic factors is sparse, we also examine decision-making, patient empowerment, and communication and trust in the patient-doctor relationship. We incorporate these important concepts into our

model.
A review of 12 studies of the doctor-patient relationship revealed that higher SES patients communicated more actively and expressively, eliciting more information from their doctor. Doctors misperceived the desire and need of patients with lower SES for information and participation in the care process, resulting in giving less information and a less participatory decision-making style.[92] Patients with higher education participated more in treatment.[93-95] Chronic disease patients who were female, younger, more affluent and had more education were more likely to want to participate in decision-making.[95]
Regarding race/ethnicity, two studies found that African Americans patients rated their physicians as more participatory if the physicians were also African American.[96, 97] Other studies found that white patients more actively participated in medical consultation, and physicians tended to talk in a more supportive style with white patients compared to non-white patients,[93] and that physicians were more verbally dominant and less patient-centered in communication with African American than white patients.[98] The Medical Outcomes Study data found that minority patients had fewer visits that were described as “participatory”.[94]
Generally, patient and physician gender also influences interactions, although one review article concluded these differences were modest.[99] Female patients tend to more actively communicate with physicians.[94, 95] Women providers generally engage in a more patient-centered, partnership-building treatment style, and are more informative and explicitly reassuring than men.[93, 94, 97] Patient-physician gender concordance or patient preference for a male or female physician may be important. For this reason our group intervention suggests considering this in selecting a PC physician.
We need to better understand gender in outcomes,[65, 100-108] because women traditionally use more PC services than men[109] and may be more open to talking about problems, they may benefit more from a PC-based model of care. PC physicians may focus on drug use problems that are more common for particular groups, and thus more likely refer them to specialty care. The use patterns of each service component (PC and drug treatment) and the relationships to outcomes may vary across subgroups. For these reasons, we will explore potential outcome differences between men and women, and across age groups.
Cross-sectional and longitudinal studies have found differences by age in drug use disorders,[110-116] and in substance abuse-related medical and psychiatric problems.[44, 55-61] Older adults are especially vulnerable to drug consequences.[117, 118] Treatment prognosis is positive,[119-121] particularly among women,[122] but few studies have examined how better service models could improve outcomes.[123, 124] Older adults have higher rates of medical conditions and may particularly benefit from continuing care involving PC. The aging of the large relatively heavy drug-using “baby boom” cohort makes it essential for policy to anticipate the potential strain on drug treatment and rising costs.[125] Age may influence patient-physician interactions, patient activation, and utilization of PC services; with PC utilization increasing with age but drug treatment peaking in middle age and then declining.[126, 127] In geriatric samples, decline in speed of mental processing and increased salience of emotional material for learning and recall have implications for clinical communication.[128] However, few studies have directly examined patient-physician communication by age.[129] Stigma around drug and alcohol problems and specialty services may be greater among older adults than younger ones.[130] If older adults utilize more PC for medical problems and preventive services, and prefer to have their substance use monitored in PC, older adults may show a greater effect of the intervention.
We will also compare psychiatric comorbidity and severity between conditions. We anticipate that the intervention would motivate patients to discuss mental health issues with their physician and that referrals for psychiatric services may increase.
Self-Help Approaches and Patient Activation. As researchers working in a health plan, we are often reminded that the drug abuse field is a leader in promotion of self-management and support systems. Other chronic disease programs (i.e., diabetes, asthma, obesity) borrow heavily from principles of 12-step and other mutual help programs and the empowerment and support they provide, as well as the self-responsibility they espouse. This speaks to the feasibility of our model: the experience that drug treatment patients have in becoming involved with self-help groups and taking responsibility for their problems is an indication that they can take a larger role in guiding their health care.
Cost Impact. Medical utilization is influenced by drug use and medical problems.[109] A large research base demonstrates that drug treatment patients have high pre-treatment medical use (e.g., ER and inpatient)[131-133] and reduced post-treatment utilization compared to others.[131, 134-138] Studies have shown reduced costs18 months post-treatment among drug treatment patients.[139] Also, patient characteristics play key roles in explaining costs over 5 years.[109] Drug treatment patients with medical conditions particularly have higher hospitalization costs.[140-142] Cost studies have been observational, and have not examined mitigating influences of medical services, nor have they been analyzed in a continuing care model. But linkages between PC and methadone or residential treatment have been related to decreases in self-reported 1-year ER and hospital use in a public sample.[32]

Although no studies have examined cost differences from a trial of a PC-based continuing care model with access to actual cost records, intervention studies support the feasibility and potential economic benefits of PC as a locus of continuing care. For example, brief interventions during medical visits have been shown to have 1- and 4-year cost benefits,[143-145] and by three years annual PC care predicted lower ER and inpatient services.[146] This experimental study will add to the literature by examining cost of PC-linked continuing care. We expect that PC visits will result in decreased inpatient and ER costs, and that regular PC visits will mediate the relationship with outcomes, including costs.

The impact of costs of continuing care in relation to patient characteristics, such as gender and age, are important to understand in order to develop economically viable models. Effective coordination of multiple health services may be necessary to support-long term recovery.[12] While treatment outcomes are better for older than younger patients, medical costs increase with age[147-150] and may not decrease as much for this group. Understanding the relationship of age and gender to cost in a PC-linked drug treatment model will also be a significant contribution to developing appropriate health policy.[125]

Feasibility. PC has traditionally played a minimal role in treating drug abuse, and the referral process between drug treatment and PC has not worked well in either integrated or contracted systems, or in public or private sectors.[12, 36] Despite past difficulties in involving PC in drug abuse services, many factors now support the feasibility of the intervention we propose. In recent years, a critical mass of influences has raised health plans' awareness of drug abuse, with the PC environment becoming more favorable to increasing its role in addressing it as a chronic condition as it does with other disorders.[12, 151-153]

The adoption of electronic medical records (EMRs) is greatly improving the capability of departments to collaborate and communicate.[11, 12] Privacy issues associated with the Code of Federal Regulations (e.g., 42 CFR), long a barrier to integrating care, are beginning to be addressed through EMRs.

The recent passage of federal alcohol and drug treatment (along with mental health) parity legislation also impacts state legislation and helps mainstream services. As mentioned earlier, the current health reform proposals also include addiction services.

The inception of NCQA as HEDIS Performance Measurement for identification, initiation and engagement is putting pressure on health plans to address drug use. As well, CPT codes and other reimbursement mechanisms for screening and brief intervention are newly available incentives that encourage health plans to address alcohol and drug use in PC. The Joint Commission for Accreditation of Healthcare Organizations has developed measures which currently are in the public comment phase substance use SBIRT measures for all hospital admissions.

Many medical and addiction research and policy organizations (e.g., NIDA, NIAAA, ASAM, SAMHSA, ONDCP, U.S. Prevention Task Force) have initiatives to increase screening, interventions, and treatment, including: brief, evidence-based screening instruments, physician handbooks, other publications, special RFAs and other research initiatives, and greater focus on PC in national meetings. Each is highlighting substance use and health care as a major initiative. As an example, in September, 2008, ONDCP held a White House Conference at which NIDA, NIAAA, SAMHSA, AHRQ, HRSA, the VA, insurance companies (Aetna, Kaiser, Blue Cross), and addiction/PC researchers in the U.S., including researchers on this study, attended. They launched a coordinated set of actions to ensure that these beginning efforts would retain staying power under a new administration. The new Administration has promised continuity in this area through ONDCP.[154] Congress has appropriated funds for CSAT to train medical residents through recently funded grants to 12 medical schools. This is greatly improving the PC environment for addressing alcohol and drug problems as part of health care (much as happened in the smoking field). As this evolves, it should facilitate PC physicians' familiarity with substance use problems.

Pain management and misuse of opiate prescriptions are a major concern of health plans and physicians. Health plans are aware that many who abuse prescription drugs have other drug use problems.[155] The FDA is developing a new Risk Evaluation Mitigation Strategy (REMS) program to be distributed this year, which will involve physician training, and 14 states have developed prescription monitoring programs.[156]

By 2002, more than 20 states were implementing Medicaid disease management programs for their PCCM and fee-for service populations.[157] (These programs have generally reduced inpatient costs, but often increased other costs, especially pharmacy, which we note could be considered a positive development in the addictions field.)

Our model is also consistent with the patient-centered, activation approach in health care advocated by AHRQ and the recent Institute of Medicine studies.[12]

We also note RWJF's 2009 Substance Abuse Research and Policy Program statement to funders of alcohol and drug abuse research (made when the program ended as they worked with experts and public and private funders to identify key areas left undone): "If policymakers and researchers could focus on only one issue in the coming five years, the most critical is the need to more fully blend addiction treatment with primary care and other medical services."[158] The focal research question was stated as: "How do we adapt addiction services for chronic care management into primary care?"

Innovation. This research is innovative in its emphasis on patient activation and integrating with mainstream

health care, and its use of an electronic medical record and related technologies to facilitate service linkages, provide patient information, and measure outcomes. Key policy implications include improving health plan structures that facilitate links between drug treatment and PC, and potential cost savings.
Relation to Center Theme. This study is related to the Center and its other components substantively and methodologically. As a *health services* study, it provides an important platform for understanding how to extend to PC the other interventions studied. Based on what we learn, we will propose next step studies of other interventions in integrated drug treatment/PC models. The Kaiser Division of Research and the Center have a long and productive relationship, now strengthened and more formalized in this Center submission. The PI of this component, Dr. Weisner, also directs the Mental Health and Addiction research program for the health plan. We also will learn how such a platform can be used by the CTN California-Arizona node to propose interventions. Finally, the component led by Dr. Prochaska and the secondary analysis led by Dr. Tsoh will integrate this study with other Center efforts to examine cross-study hypotheses, and study data will be used by studies of the costs and cost effectiveness of treatment directed by Dr. Barnett. The component will receive administrative support from the Scientific and Administration core led by Dr. Guydish, and Statistics and Health Economics Core directed by Dr. Delucchi.
Summary. The premise of this study is that specialty drug treatment, including aftercare, always ends at some point, but could be extended by a continuing care model through linkage with PC. This could also function to identify individuals at risk for relapse and continue to help those who drop out of treatment early. Learning from chronic care treatment for other medical conditions, we see that a disease management approach linking patients to PC following drug treatment, regardless of the length and intensity of initial treatment, has promise for our field. This extends the reach of what is offered in drug treatment-based aftercare, and our sample size will allow us to examine the outcomes of those who drop out of drug treatment as well. The study is based on key results from several randomized studies showing that "during treatment" integrated PC and drug treatment is beneficial,[30, 31, 35] as is PC linkage after detox.[37] In addition, observational studies in private and public settings have found that ongoing PC is related to better long-term outcomes.[40, 44] We are fortunate to have as consultants the primary leader of continuing and chronic care models in the medical field, Ed Wagner, M.D., and the PI of the randomized PC linkage study which has informed our study, Jeffrey Samet, M.D. He has also been the representative from the drug abuse field presenting to physicians how to address drug abuse and interact with drug treatment programs in the NIH Contemporary Clinical Medicine-Great Teachers Grand Round webcasts.[159] Both consultants have been advisors on our study design and will be ongoing consultants. Dr. Wagner will particularly consult on the patient activation and overall chronic care model, and Dr. Samet on the linkage intervention. In addition, Dr. McKay's presence as a Consultant on the Core of the Center will greatly benefit this component.
We extend the existing research to examine an intervention that begins to activate patients in using health care and links them with PC after drug treatment.

12.2 Preliminary studies:

PRELIMINARY STUDIES

History. Our research at the Kaiser Permanente (KP) Division of Research (DOR) has been instrumental in developing the Continuing Care intervention and in informing our specific aims. These studies were conducted in both KP and public sector programs (e.g., drug treatment and public health clinics, mobile health vans) and other private programs, and in drug treatment, PC, and in the general KP membership, including adult and adolescent populations. This work is fundamental to the hypotheses we propose, and speaks to the collective experience and commitment of our team to successfully carry out this research. The budget justification explains the role and relevant background of members of the research team. Dr. Weisner has a joint appointment at UCSF and DOR, where she is the Associate Director for Health Services Research for the Northern California region, and also leads DOR's mental health and addiction research.
Directly Relevant Recent and Ongoing Work Which Inform Our Intervention. We first describe research contributions of DOR researchers on similar studies in other chronic diseases which have informed our intervention. This is followed by discussion of research our group has conducted in PC and drug treatment, as well as findings on medical conditions, medical services, drug treatment outcomes, and treatment readmission, including gender, age, and race/ethnicity factors, and cost which have contributed to our study design and hypotheses. Finally, we describe the relationships that we have developed with the health plan, and PC in particular, that will facilitate the success of the study.
Studies on Chronic Care within DOR. The DOR has a long track record of conducting rigorous chronic care studies within PC. The more recent three examples include the TRIAD Study, a CDC, NIDDK-funded study of diabetes in managed care examining relationships of process with outcomes measuring diabetes quality

and patient and system-level predictors of outcomes. This is one of the areas where patient activation has been associated with outcomes.[13] Another example is the AHRQ-funded cluster-randomized cluster trial, “Intensification, Feedback, and Outcomes (INFO) to Reduce Cardiovascular Disease,” within KP PC clinics which tested the impact of service innovations on improving chronic disease outcomes. It incorporated patient-level information on the need for treatment intensification into the population management software currently used by care teams that support PC providers in managing patients with chronic conditions. Preliminary findings suggest improved treatment intensification rates in intervention clinics compared to control clinics, and may also improve intermediate risk factor levels such as blood pressure and LDL-c. DOR has also collaborated with clinical partners to train, implement, and study a new depression care model in which PC nurses provide Telecare interventions for depression.[160] These study investigators work in close proximity to our research group and provide advice and collaboration. The studies demonstrate DOR’s large-scale commitment to innovative services research on chronic conditions and the receptive health plan environment for the proposed study. Dr. Weisner’s recent appointment as DOR Associate Director for Health Services Research also provides new opportunities for addictions research and study implementation in PC.

Studies by the Drug and Alcohol Research Team at DOR. Research on effectiveness and implementation, conducted in drug treatment and PC, and conceptual studies on blending research and practice by Sterling,[161] have guided our planning of the intervention. This work has included qualitative studies and input from relevant KP stakeholder groups necessary to the success of an intervention (i.e., administrators, PC and drug treatment leaders, and patients. The model is published in NIAAA’s Alcohol Health and Research journal.[161] Research at DOR has included randomized trials, effectiveness studies, and implementation of findings. We have studied inclusion of medical care as part of drug treatment,[30, 39] development of dual diagnosis liaison teams across the region to provide integrated drug and mental health services and promoting the “no wrong door” concept,[162-164] and the development of a screener in Adolescent Medicine based on our adolescent study.[161, 164-168] Sterling is currently completing a pilot study with KP foundation funds on screening, referral and management of adolescents with substance use conditions in Adolescent Medicine. In each of these studies, the primary study team members have the same roles as in this proposed study.

Studies of Primary Care. The proposed study demands effective collaborations with PC as well as drug treatment: We have conducted studies in PC settings, both within and outside KP, with high recruitment and response rates. Mertens led a NIDA- and RWJF-funded survey in two KP PC clinics. Rates of hazardous drinking were 7.5%, of other drug use 3%, and combined were 10% in KP PC clinics.[169] This study gave the research group “hands on” experience in PC clinics, including sampling, conducting the study with minimal disturbance to busy clinics, and understanding the roles and formal and informal relationships among physicians and other providers. These skills will be critical to implementing the linkage phone call, staff informational sessions and EMR reminders in our proposed study.

Mertens, Weisner, and Sterling also conducted a survey of patients treated by public mobile health vans.[170] Rates of hazardous alcohol and drug use problems were 30% and 27% respectively. Hinman and the DOR group are conducting a study on drug and alcohol diagnoses and related medical conditions and disparities in 8 public health clinics in an ethnically diverse county, and a screening study in a public PC clinic. We are assessing rates of drug use and problems and medical conditions and conducting a medical records review to measure prevalence and characteristics of the population. These studies have added to our expertise on needs and organization in other health systems. Our plans include continuing to work with public health administrators and clinicians, building on what we learn from their PC clinics as well as this proposed study, to adopt our continuing care intervention to public PC and drug/alcohol systems.

Other related studies include Mertens’ and Weisner’s NIDA study of screening in 14 public PC clinics in Cape Town, South Africa townships.[171-177] Based on findings, Mertens is conducting a NIDA brief intervention trial in one of the clinics, with recruitment recently ended (85% recruitment rate), and follow-ups to be completed in 3/09 (83% response rate). Thus our work within KP has been translated to other settings.

Surveys of Health Providers. Sterling and Weisner are conducting a NIAAA survey of KP pediatric and adolescent medicine physicians which examines barriers to addressing drug and alcohol problems. Knowledge gained has been useful in designing this intervention. Of note is that the web-based survey was conducted within a 6 month time period and had a 76% response rate, which is much higher than other physician surveys, and demonstrates our success in working with PC clinicians.

Medical Services in the Context of Drug Treatment. Benefits of integrated medical and drug treatment were found in the NIDA Integrated Care Study; integrated services were related to better 6-month outcomes for those with substance abuse-related medical conditions (SAMC) (for both medical and psychiatric conditions),[30] and this benefit extended to 5 years.[39] Integrated services were cost effective (incremental cost-effectiveness ratio per additional integrated SAMC patient abstinent: $1,581), and remained so at 5 years.[39] For those with SAMCs, those with 2 – 10 post-intake PC visits were more likely to be remitted at 5-

years than those with fewer visits; the finding remained when controlling for initial randomization to integrated treatment.[39] Understanding "integrated care" has important implications for the proposed study. We found a higher rate of new conditions identified in integrated care, suggesting that disorders may be overlooked in PC. Two were pain-related, possibly associated with prescription drug misuse.

Our recent study of pressures to enter treatment found that pressures from physicians was related to better outcomes.[178] These findings (in conjunction with the 5-year findings above) suggest that outcome differences may be due to the content of the physician/patient interaction (physicians were influenced by their contact with addiction counselors in the drug treatment program). This has informed our intervention; we believe that our patient activation approach and linkage phone call will facilitate the content of this interaction for PC physicians outside of the drug treatment program. In addition to measuring medical visits and prescriptions, we will also measure "type of medical visit," including preventive care procedures (e.g., cholesterol tests, flu shots, mammograms, sigmoidoscopies), and whether physicians asked about drug and alcohol use,[179] using self report and the KP EMR, for both study arms.

Informing our intervention, we also examined "missed opportunities" in addressing substance use in PC in county-wide public and private health systems. Independent of public/private sector, women and younger individuals were less likely to have it addressed.[179] Contacts with PC were related to less use at 5 years.[180] These findings highlight the potentially important role of PC in the long-term treatment of drug abuse. Drug treatment may benefit from a disease management approach similar to that for other chronic conditions: specialty care when the condition is severe followed by services in PC when the condition is stabilized, with specialty consults or return to specialty treatment when needed. We will examine differences in health problems of continuing care and usual care over time and whether differences subside as patients abstain/decrease use, or receive preventive services, as well as the contributing impact of individual characteristics including gender, age and psychiatric status.

Medical conditions. Our studies of patients with medical (and psychiatric) conditions have been unique in addressing the influence of both medical conditions and services on outcomes.[30, 39, 44, 169, 181] This has direct implications for understanding the role of PC with these patients over time, and has been influential in working with the health plan to implement screening questions in the EMR. We have measured costs and identified medical conditions in drug treatment clients,[30, 44, 167] in hazardous drinkers and drug users within PC,[169, 177] and in problem drinkers within the KP membership.[182-184] These medical comorbidities highlight the importance of PC to patients with drug use disorders. The Mertens PC screening study[169] found that many medical conditions (e.g., hypertension, COPD, pneumonia, depression, anxiety, major psychoses, and injuries) had higher rates for hazardous alcohol and drug users than others in PC. These were also among the most costly to health care.

As part of the Integrated Care Study, we compared a KP treatment sample with KP member matched controls on medical conditions.[185] Treatment patients had higher rates of injuries, poisonings, depression, anxiety, psychoses, lower back pain, hypertension, headache, asthma, acid-related disorders, arthritis, liver cirrhoses, hepatitis C, diseases of the pancreas, and chronic obstructive pulmonary disease (COPD). We compared cases and matched controls in years 3-5, and from 6-9 years after intake. In the 3rd to 5th years, cases had higher prevalence of acid-related disorders, COPD, headache, hypertension, injuries/overdoses, lower back pain, cirrhosis, diseases of the pancreas, Hepatitis C, depression, anxiety and eating disorders, and major psychoses. In the 6th - 9th years, cases had higher rates of a majority of these disorders, but there were no differences for two substance abuse related medical conditions that were found earlier and that may remit upon reducing drinking--hypertension and acid-related disorders. These findings highlight the importance of integrating PC services in the treatment of drug abuse, both in terms of providing needed medical care and in regard to cost, and have been important in interesting PC and drug treatment clinicians in this study.

Drug Treatment Entry. Our research on factors influencing treatment entry and initiation[63, 68, 115, 126, 186-194] also inform this study. As these studies have shown, "how one gets to treatment" affects one's motivation and perhaps also the PC relationship. Most common sources in the KP samples are employers, family, and doctors; on the whole, men reported pressure from families and employers. Physician referrals have been under-represented,[186] but a higher proportion of women receive them than do men.[119, 195] In regard to age, only older adults reported more pressure from doctors than other sources.[116] We developed models predicting the likelihood of returning after intake to start treatment.[195] Over time, as an individual's problems became increasingly severe, referrals from physicians become more frequent.[126, 196] This is consistent with the experience of Dr. Mertens' screening study, the public/private missed opportunities study,[179] and our qualitative work that only the more severe patients are noticed and have their drinking addressed in PC. Thus it is important to take a more systematic approach to managing patients within PC, as proposed in our intervention. In our study we measure physician referrals back to drug abuse treatment and physician monitoring of drug problems during PC appointments.

Treatment Readmission to Drug Abuse Treatment. An individual's overall treatment experience is important in thinking through a continuing care approach; we will examine the relationships between drug treatment and PC visits in treatment readmissions. We have examined both rates and characteristics associated with readmission at one year and over 5 years.[197] Overall, 38% were readmitted by 5 years. Women (44%) had higher rates than men (34%); as were those who had medical and psychiatric disorders. Higher baseline psychiatric and alcohol ASI scores were predictors. In the NIDA adolescent study, in support of a continuing care model, we found that readmissions between the 1- and 5-year interviews predicted abstinence at 5 years, for both the full sample and those not abstinent at 6 months.[7] In the proposed study, we will examine the impact of the intervention on readmission, which could influence outcomes at follow up. We will use the EMR to examine referral sources (whether the PC physician referred) for readmissions and compare between conditions, and we will ask self-report questions of patients on the content of conversations with PC physicians and on reasons for readmission.
Demographic Characteristics of gender, age, and race/ethnicity. We have powered the study to examine gender, age, and in many cases, race/ethnicity effects, because of the literature and our findings regarding these key characteristics. Our studies have found different intake profiles by gender; women had lower levels of social and financial resources, higher levels of psychiatric severity, medical conditions, and different dependence patterns.[44, 115, 139, 198] Outcome data through 9 years indicate that women consistently had higher levels of psychological severity, while gender differences in medical and employment status became insignificant. By 5 years post treatment, women were more likely to have received medical services and medications for physical problems, and services and medications for family and psychiatric problems, testing, and individual psychiatric sessions.[109, 199] Gender findings suggest a non-linear trajectory influenced by ongoing treatment and psychiatric services, which may lead to differences in response to the intervention. The intervention incorporates and measures appropriate routine health care recommendations that differ by gender (e.g., reproductive health and gender relevant preventive services).
We have also found important age differences in medical consequences of alcohol and drug use and in outcomes and use of PC by age that call for examining the outcomes of the intervention by age. Older adults had more medical conditions, and showed more improvement in abstinence and in medical consequences.[116] We found declines in medical ASI scores at 6 months, with a linear relationship to age group: older patients showed more improvement at 6 months.[116] Based on this, younger and older adults may have different responses to our intervention. If older adults have worse physical health and are not well connected to medical services, they may derive even greater benefit than younger adults from an intervention actively connecting them to medical services in a continuing care model. On the other hand, younger patients may be more attracted to using the computer-based learning environment which will be available as part of the intervention.
Findings on race/ethnicity in our studies also suggest the importance of examining it as a factor in the success of the intervention. For example, in our drug treatment samples, African American women had shorter retention rates in treatment than other women,[119] as did African American adolescents in general,[200, 201] and drug-use related outcomes have differed by race/ethnicity. In addition to these findings, the literature on patient communication suggests that the role of PC and patient activation may be particularly important for ethnic minority populations.

Psychiatric severity. Treatment of individuals with co-occurring conditions is of major policy and clinical concern. High proportions of the KP drug treatment samples have co-occurring psychiatric disorders,[163, 199] and these patients have more medical problems than others. It will be important to examine whether they equally benefit from enhanced linkages with PC. Because discussions between continuing care patients and their PCs about problems may increase, and because patients may become more activated in managing their health care, we will examine whether patients in this arm receive more mental health services.
Analyses of Costs. We have compared KP drug treatment samples to controls from the membership (matched on age, sex, geographic area and length of enrollment) on cost impacts 6 months prior- and 18-months post-treatment,[139] as well as over 3 and 5 years.[109, 139] Treatment samples used more services pre-treatment than matched controls across all departments. We also examined the relationship between patient characteristics (particularly gender, age, and medical and psychiatric conditions) and utilization and cost.[109] Inpatient (35%) and ER (39%) costs had the most significant post-treatment reductions. We found a 26% reduction in total medical costs, adjusting for age and gender. Women had higher costs than men pre-treatment and substantial reductions post-treatment. We examined whether short-term cost reductions remained over 5-years.[109] In the drug treatment sample, inpatient and ER use declined gradually. Average ER costs per member-month declined monotonically from $19.63 to $12.15. Average medical costs per member- month peaked at 6 months post-intake (primarily due to treatment costs) and then declined gradually. By 5 years, costs had declined to $234.61 per member-month, which was lower than the pre-intake level of $389.81. Hospital costs also decreased from $187.31 to $105.77. We found no trends in costs

for the matched controls for the same period.
To understand the factors that affect 5 year cost trajectories, we have used hierarchical modelling (HLM).[202, 203, 204] We examined cost trajectories (total, inpatient, ER and PC) 5 years post-intake and the effect of individual, treatment, and extra-treatment characteristics on these components.[109] PC costs were higher on average at baseline for women but they had a steeper decline over time. While there was a significant decrease in average inpatient cost, those over age 40 had smaller declines than those younger.[147-150] Higher medical and psychiatric severity was associated with higher average inpatient and ER costs. Average medical costs for the drug treatment cohort (4 times that of the matched sample in the 6 months prior to intake), was only twice as high by year 5.
We examined 9-year cost trajectories of this sample as well. As with the 5-year cost trajectories, 9 year post treatment, long-term utilization and cost trajectories were non-linear. We also found average costs increased by 9-years for matched controls, possibly due to aging and readmissions. Thus, declining (or flat) utilization/cost patterns observed through 5 years were not sustained over long periods. This suggests the importance of examining continuing care; for drug abuse, as for other chronic diseases, identifying relapsing patients earlier and readmitting them to treatment earlier can prevent critical and acute events leading to ER or inpatient use (and associated high costs) which typically triggers treatment readmission. The proposed intervention makes PC linkage the foundation of a continuing care model and should facilitate readmission earlier in the relapse process, resulting in lower costs.
Other Relevant Developmental Work. This component is the product of a long-term process that includes qualitative studies and collaborative work in the Kaiser health plan, the longitudinal studies described above, and our ongoing collaboration with the Center. The research team has a strong history of working with KP clinicians and administrators on studies and implementation of results, as well as with Center investigators. Particularly in the past two years, we have worked closely with administrative and clinical groups to develop this intervention; including conducting qualitative interviews with PC and drug treatment clinicians. We have worked with the KP Chemical Dependency Quality Improvement Committee (CDQIC) to develop the intervention, study design, and training methods in order to integrate the study effectively, and to optimize its feasibility for adoption if the intervention is successful. The CDQIC includes representatives from all regional drug treatment programs, as well as PC clinician representation. We have also attended the bimonthly PC clinician regional CD Chief meetings to understand the PC-related issues for integrating drug use with PC. Evidence of the effectiveness of our relationship is our success in incorporating screening/monitoring tools into the EMR.
We also regularly attend the Chronic Pain Steering Committee to understand how disease management works with the substance use-related condition of pain. We attend meetings of the larger Chronic Conditions Work Group and meet with chronic conditions regional leaders. Multiple grand rounds and other seminars on disease management and measurement (e.g., in diabetes, cancer, and depression) are held at DOR,[30] and we have consulted closely with those researchers to find commonalities and understand how to develop the most efficient approach for our field. The environment at DOR is very conducive to such interactions. Because DOR is located one block from KP's regional headquarters (and two blocks from KP's national headquarters), we have easy access to clinicians and administrators when they attend other meetings.
Summary The proposed study is supported by the larger literature in the field and a more promising policy and clinical environment for working with PC than has existed previously. The study draws considerably on the work of our research team, including relevant research findings and our history of successful collaborations with the health plan. This includes our success in incorporating alcohol and drug use questions in the EMR. As we have reviewed, other chronic care studies within the health plan have influenced our intervention and design, and those investigators have advised us. Our research is consistent with earlier drug treatment studies in regard to the role of PC in drug abuse outcomes, including a trial of integrated services during drug treatment, as well as observational studies of the importance of PC after treatment. Our consistent findings across studies, particularly on the role of patient demographics and medical and psychiatric comorbidities (in drug treatment and in PC) argue for the importance of this study examining these factors in regard to continuing care.

12.3 References:

REFERENCES for TRCContinuing Care Study

1. McLellan AT, Lewis DC, O'Brien CP, Kleber HD. Drug dependence, a chronic medical illness: implications for treatment, insurance, and outcomes evaluation. *JAMA.* Oct 4 2000;284(13):1689-1695.
2. Tsuang MT, Lyons MJ, Eisen SA, et al. Genetic influences on DSM-III-R drug abuse and

dependence: a study of 3,372 twin pairs. *Am J Med Genet.* Sep 20 1996;67(5):473-477.

3. Kendler KS, Prescott CA. Cannabis use, abuse, and dependence in a population-based sample of female twins. *Am J Psychiatry.* Aug 1998;155(8):1016-1022.

4. Schuckit MA, Smith TL. An 8-year follow-up of 450 sons of alcoholic and control subjects. *Arch Gen Psychiatry.* Mar 1996;53(3):202-210.

5. Dennis M, Scott CK. Managing addiction as a chronic condition. *Addict Sci Clin Pract.* Dec 2007;4(1):45-55.

6. Dennis ML, Scott CK, Funk R, Foss MA. The duration and correlates of addiction and treatment careers. *J Subst Abuse Treat.* 2005;28 Suppl 1:S51-62.

7. Weisner C, Ray GT, Mertens J, Satre DD, Moore C. Short-term alcohol and drug treatment outcomes predict long-term outcome. *Drug Alcohol Depend.* Sep 10 2003;71(3):281-294.

8. Hser YI, Anglin MD, Grella C, Longshore D, Prendergast ML. Drug treatment careers: a conceptual framework and existing research findings. *J Subst Abuse Treat.* Nov-Dec 1997;14(6):543-558.

9. Bodenheimer T, Wagner EH, Grumbach K. Improving primary care for patients with chronic illness: the chronic care model, Part 2. *JAMA.* Oct 16 2002;288(15):1909-1914.

10. Von Korff M, Gruman J, Schaefer J, Curry SJ, Wagner EH. Collaborative management of chronic illness. *Ann Intern Med.* Dec 15 1997;127(12):1097-1102.

11. Institute of Medicine. *Crossing the Quality Chasm: A New Health System for the 21st Century.* Washington, DC: National Academy Press; 2001.

12. Institute of Medicine. *Improving the Quality of Health Care for Mental and Substance-Use Conditions: Quality Chasm Series.* Washington, DC: National Academies Press; 2006.

13. Mosen DM, Schmittdiel J, Hibbard J, Sobel D, Remmers C, Bellows J. Is patient activation associated with outcomes of care for adults with chronic conditions? *J Ambul Care Manage.* Jan-Mar 2007;30(1):21-29.

14. Schmittdiel J, Mosen DM, Glasgow RE, Hibbard J, Remmers C, Bellows J. Patient Assessment of Chronic Illness Care (PACIC) and improved patient-centered outcomes for chronic conditions. *J Gen Intern Med.* Jan 2008;23(1):77-80.

15. Hibbard JH, Stockard J, Mahoney ER, Tusler M. Development of the Patient Activation Measure (PAM): conceptualizing and measuring activation in patients and consumers. *Health Serv Res.* Aug 2004;39(4 Pt 1):1005-1026.

16. Holman H, Lorig K. Patients as partners in managing chronic disease. Partnership is a prerequisite for effective and efficient health care. *Bmj.* Feb 26 2000;320(7234):526-527.

17. Wagner EH, Austin BT, Von Korff M. Organizing care for patients with chronic illness. *Milbank Q.* 1996;74(4):511-544.

18. Wagner EH, Austin BT, Von Korff M. Improving outcomes in chronic illness. *Manag Care Q.* Spring 1996;4(2):12-25.

19. McKay JR, Carise D, Dennis ML, et al. Extending the benefits of addiction treatment: Practical strategies for continuing care and recovery. *J Subst Abuse Treat.* Mar 2009;36(2):127-130.

20. McKay JR. Continuing care research: What we have learned and where we are going. *J Subst Abuse Treat.* Mar 2009;36(2):131-145.

21. McKay JR, Carise D. State of the science: Extending the benefits of addiction treatment. *J Subst Abuse Treat.* Mar 2009;36(2):172-173.

22. McKay JR. Is there a case for extended interventions for alcohol and drug use disorders? *Addiction.* Nov 2005;100(11):1594-1610.

23. McKay JR, Lynch KG, Shepard DS, Pettinati HM. The effectiveness of telephone-based continuing care for alcohol and cocaine dependence: 24-month outcomes. *Arch Gen Psychiatry.* Feb 2005;62(2):199-207.

24. Grella CE, Greenwell L. Treatment needs and completion of community-based aftercare among substance-abusing women offenders. *Womens Health Issues.* Jul-Aug 2007;17(4):244-255.

25. Godley SH, Godley MD, Wright KL, Funk RR, Petry NM. Contingent reinforcement of personal goal activities for adolescents with substance use disorders during post-residential continuing care. *Am J Addict.* Jul-Aug 2008;17(4):278-286.

26. Godley MD, Godley SH, Dennis ML, Funk R, Passetti LL. Preliminary outcomes from the assertive continuing care experiment for adolescents discharged from residential treatment. *J Subst Abuse Treat.* Jul

2002;23(1):21-32.
27. Godley MD, Godley SH, Dennis ML, Funk RR, Passetti LL. The effect of assertive continuing care on continuing care linkage, adherence and abstinence following residential treatment for adolescents with substance use disorders. *Addiction.* Jan 2007;102(1):81-93.
28. Ritsher JB, McKellar JD, Finney JW, Otilingam PG, Moos RH. Psychiatric comorbidity, continuing care and mutual help as predictors of five-year remission from substance use disorders. *JSA.* Nov 2002;63(6):709-715.
29. Dennis M, Scott CK, Funk R. An experimental evaluation of recovery management checkups (RMC) for people with chronic substance use disorders. *Evaluation and Program Planning.* Aug 2003;26(3):339-352.
30. Weisner C, Mertens J, Parthasarathy S, Moore C, Lu Y. Integrating primary medical care with addiction treatment: a randomized controlled trial. *JAMA: Journal of the American Medical Association.* Oct 10 2001;286(14):1715-1723.
31. Friedmann PD, Saitz R, Samet JH. Management of adults recovering from alcohol or other drug problems: relapse prevention in primary care. *JAMA.* 1998;279(15):1227-1231.
32. Friedmann PD, Hendrickson JC, Gerstein DR, Zhang Z, Stein MD. Do mechanisms that link addiction treatment patients to primary care influence subsequent utilization of emergency and hospital care? *Med Care.* Jan 2006;44(1):8-15.
33. Friedmann PD, Zhang Z, Hendrickson J, Stein MD, Gerstein DR. Effect of primary medical care on addiction and medical severity in substance abuse treatment programs. *J Gen Intern Med.* Jan 2003;18(1):1-8.
34. McLellan AT, Alterman AI, Metzger DS, et al. Similarity of outcome predictors across opiate, cocaine and alcohol treatments: role of treatment services. *J Consult Clin Psychol.* Dec 1994;62(6):1141-1158.
35. Umbricht-Schneiter A, Ginn DH, Pabst KM, Bigelow GE. Providing medical care to methadone clinic patients: referral vs. on-site care. *American Journal of Public Health.* Feb 1994;84(2):207-210.
36. D'Aunno T. Linking substance-abuse treatment and primary health care. In: Egertson JA, Fox DM, Leshner AI, eds. *Treating Drug Abusers Effectively.* Oxford: Blackwell; 1997:311-331.
37. Samet JH, Larson MJ, Horton NJ, Doyle K, Winter M, Saitz R. Linking alcohol- and drug-dependent adults to primary medical care: A randomized controlled trial of a multi-disciplinary health intervention in a detoxification unit. *Addiction.* Apr 2003;98(4):509-516.
38. Saitz R, Larson MJ, Horton NJ, Winter M, Samet JH. Linkage with primary medical care in a prospective cohort of adults with addictions in inpatient detoxification: room for improvement. *Health Serv Res.* Jun 2004;39(3):587-606.
39. Mertens JR, Flisher AJ, Satre DD, Weisner CM. The role of medical conditions and primary care services in 5-year substance use outcomes among chemical dependency treatment patients. *Drug Alcohol Depend.* Nov 1 2008;98(1-2):45-53.
40. Saitz R, Horton NJ, Larson MJ, Winter M, Samet JH. Primary medical care and reductions in addiction severity: A prospective cohort study. *Addiction.* Jan 2005;100(1):70-78.
41. Lorig KR, Holman H. Self-management education: history, definition, outcomes, and mechanisms. *Ann Behav Med.* Aug 2003;26(1):1-7.
42. Bodenheimer T, Wagner EH, Grumbach K. Improving primary care for patients with chronic illness. *JAMA.* Oct 9 2002;288(14):1775-1779.
43. Stein MD. Medical consequences of substance abuse. *Psychiatr Clin North Am.* Jun 1999;22(2):351-370.
44. Mertens JR, Lu YW, Parthasarathy S, Moore C, Weisner CM. Medical and psychiatric conditions of alcohol and drug treatment patients in an HMO: comparison with matched controls. *Arch Intern Med.* Nov 10 2003;163(20):2511-2517.
45. Fleming MF, Barry KL. *Addictive Disorders: A Practical Guide to Treatment.* St. Louis, MO: Mosby-Year Book primary care series; 1992.
46. Chou SP, Grant BF, Dawson DA. Medical consequences of alcohol consumption--United States, 1992. *Alcoholism: Clinical and Experimental Research.* Nov 1996;20(8):1423-1429.
47. Redelmeier DA, Tan SH, Booth GL. The treatment of unrelated disorders in patients with chronic medical diseases. *N Engl J Med.* May 21 1998;338(21):1516-1520.
48. Gonzales JJ, Insel TR. The conundrum of co-occurring mental and substance use disorders: opportunities for research. *Biol Psychiatry.* Nov 15 2004;56(10):723-725.

49. Volkow ND. The reality of comorbidity: depression and drug abuse. *Biol Psychiatry.* Nov 15 2004;56(10):714-717.

50. Montoya ID. Infectious diseases and anemia in a sample of out-of-treatment drug users. *Am J Manag Care.* 1998;4(9):1257-1264.

51. Edwards G, Peters TJ. Conclusions. In: Peters TJ, ed. *Alcohol Misuse: A European Perspective*. Amsterdam: Harwood Academic Publisher; 1996:195-197.

52. Sikkink J, Fleming MF. Adverse health effects and medical complications of alcohol, nicotine, and drug abuse. In: Fleming MF, Barry KL, eds. *Addictive Disorders: A Practical Guide to Treatment*. St. Louis: Mosby-Year Book Primary Care Series; 1992:145-168.

53. Weisner C. Chronic alcohol and drug abuse. In: Newcomer RJ, Benjamin AE, eds. *Indicators of Chronic Health Conditions: Monitoring Community-Level Delivery Systems*. Baltimore, MD: Johns Hopkins University Press; 1997:260-301.

54. National Institute on Alcohol Abuse and Alcoholism. *Seventh Special Report to the U.S. Congress on Alcohol and Health.* Washington, DC: U.S. Government Printing Office; 1990. DHHS Publication No. ADM 90-1656.

55. Moos RH, Brennan PL, Mertens JR. Diagnostic subgroups and predictors of one-year readmission among late-middle-aged and older substance abuse patients. *JSA.* Mar 1994;55(2):173-183.

56. Clark WB, Hilton ME, eds. *Alcohol in America: Drinking Practices and Problems*. Albany, NY: State University of New York Press; 1991. SUNY Series in New Social Studies on Alcohol and Drugs

57. Moos RH, Brennan PL, Mertens JR. Mortality rates and predictors of mortality among late-middle-aged and older substance abuse patients. *Alcoholism: Clinical and Experimental Research.* Feb 1994;18(1):187-195.

58. Barker SB, Kerns LL, Schnoll SH. Assessment of medical history, health status, intoxication, and withdrawal. In: Rounsaville BJ, Tims FM, Horton AM, Jr., Sowder BJ, eds. *Diagnostic Source Book on Drug Abuse Research and Treatment*. Rockville, MD: U.S. National Institute on Drug Abuse; 1993:35-48.

59. National Institute on Alcohol Abuse and Alcoholism. *Eighth Special Report to the U.S. Congress on Alcohol and Health.* Washington: U.S. Department of Health and Human Services; 1993. DHHS publication (ADM)90-1656.

60. Brunswick AF, Messeri P. Timing of first drug treatment: A longitudinal study of urban black youth. *Contemporary Drug Problems.* Fall 1985;12(3):401-418.

61. Braude MC. Drugs and drug interactions in the elderly woman. In: Ray BA, Braude MC, eds. *Women and Drugs: A New Era for Research*. Rockville, MD: U.S. National Institute on Drug Abuse; 1986:58-64.

62. Weisner C, Schmidt LA. Expanding the frame of health services research in the drug abuse field. *Health Serv Res.* Dec 1995;30(5):707-726.

63. Weisner C, Schmidt LA. Rethinking access to alcohol treatment. *Recent Dev Alcohol.* 2001;15:107-136.

64. Gerstein DR, Ewin LS. Treating drug problems. *NEJM.* 1990;323(12):844-848.

65. Institute of Medicine. *Broadening the Base of Treatment for Alcohol Problems*. Washington, DC: National Academy Press; 1990.

66. Wheeler JR, Fadel H, D'Aunno TA. Ownership and performance of outpatient substance abuse treatment centers. *Am J Public Health.* May 1992;82(5):711-718.

67. Trabin T, Freeman M. *Managed Behavioral Healthcare: History, Models, Strategic Challenges, & Future Course*. Rockville, MD: John Wiley & Sons; 1995.

68. McLellan AT, Weisner C. Achieving the public health and safety potential of substance abuse treatments: Implications for patient referral, treatment "matching," and outcome evaluation. In: Bickel WK, DeGrandpre RJ, eds. *Drug Policy and Human Nature: Psychological Perspectives on the Prevention, Management and Treatment of Illicit Drug Abuse*. New York: Plenum Press; 1996:127-154.

69. Bobo JK, Lando HA, Walker RD, McIlvain HE. Predictors of tobacco quit attempts among recovering alcoholics. *J Subst Abuse.* 1996;8(4):431-443.

70. Compton WM, Thomas YF, Stinson FS, Grant BF. Prevalence, correlates, disability, and comorbidity of DSM-IV drug abuse and dependence in the United States: results from the national epidemiologic survey on alcohol and related conditions. *Archives of General Psychiatry.* May 2007;64(5):566-576.

71. Kalman D. Smoking cessation treatment for substance misusers in early recovery: a review of the literature and recommendations for practice. *Subst Use Misuse.* Aug 1998;33(10):2021-2047.

72. Richter KP, Ahluwalia HK, Mosier MC, Nazir N, Ahluwalia JS. A population-based study of cigarette

smoking among illicit drug users in the United States. *Addiction.* Jul 2002;97(7):861-869.

73. Friedmann PD, Jiang L, Richter KP. Cigarette smoking cessation services in outpatient substance abuse treatment programs in the United States. *J Subst Abuse Treat.* Mar 2007;34(2):165-172.

74. Ziedonis DM, Guydish J, Williams J, Steinberg M, Foulds J. Barriers and solutions to addressing tobacco dependence in addiction treatment programs. *Alcohol Res. Health.* 2006;29(3):228-235.

75. Bobo JK, Davis CM. Recovering staff and smoking in chemical dependency programs in rural Nebraska. *J Subst Abuse Treat.* Mar-Apr 1993;10(2):221-227.

76. Fuller BE, Guydish J, Tsoh J, et al. Attitudes toward the integration of smoking cessation treatment into drug abuse clinics. *J Subst Abuse Treat.* Jan 2007;32(1):53-60.

77. Myers MG, MacPherson L. Smoking cessation efforts among substance abusing adolescents. *Drug Alcohol Depend.* Feb 7 2004;73(2):209-213.

78. Hurt RD, Patten CA. Treatment of tobacco dependence in alcoholics. *Recent Dev Alcohol.* 2003;16:335-359.

79. Kohn CS, Tsoh JY, Weisner CM. Changes in smoking status among substance abusers: baseline characteristics and abstinence from alcohol and drugs at 12-month follow-up. *Drug Alcohol Depend.* Jan 24 2003;69(1):61-71.

80. Lemon SC, Friedmann PD, Stein MD. The impact of smoking cessation on drug abuse treatment outcome. *Addict Behav.* Sep 2003;28(7):1323-1331.

81. Prochaska JJ, Delucchi K, Hall SM. A meta-analysis of smoking cessation interventions with individuals in substance abuse treatment or recovery. *J Consult Clin Psychol.* Dec 2004;72(6):1144-1156.

82. Satre DD, Gordon NP, Weisner C. Alcohol consumption, medical conditions, and health behavior among older adults. *American Journal of Health Behavior.* May-Jun 2007;31(3):238-248.

83. Hser YI, McCarthy WJ, Anglin MD. Tobacco use as a distal predictor of mortality among long-term narcotics addicts. *Prev Med.* Jan 1994;23(1):61-69.

84. Hurt RD, Offord KP, Croghan IT, et al. Mortality following inpatient addictions treatment. Role of tobacco use in a community-based cohort. *JAMA.* Apr 10 1996;275(14):1097-1103.

85. Booth BM, Blow FC, Cook CA, Bunn JY, Fortney JC. Relationship between inpatient alcoholism treatment and longitudinal changes in health care utilization. *JSA.* 1997;58(6):625-637.

86. Skog OJ, Duckert F. Development of alcoholics' and heavy drinkers' consumption: a longitudinal study. *JSA.* 1993;54(2):178-188.

87. Booth BM, Yates WR, Petty F, Brown K. Patient factors predicting early alcohol-related readmissions for alcoholics: role of alcoholism severity and psychiatric comorbidity. *JSA.* Jan 1991;52(1):37-43.

88. Moos RH, Mertens JR, Brennan PL. Rates and predictors of four-year readmission among late-middle-aged and older substance abuse patients. *JSA.* Sep 1994;55(5):561-570.

89. Phibbs CS, Swindle RW, Recine B. Does case mix matter for substance abuse treatment? A comparison of observed and case mix-adjusted readmission rates for inpatient substance abuse treatment in the Department of Veterans Affairs. *Health Serv Res.* 1997;31(6):755-771.

90. Brennan PL, Kagay CR, Geppert JJ, Moos RH. Elderly Medicare inpatients with substance use disorders: characteristics and predictors of hospital readmissions over a four- year interval. *JSA.* 2000;61(6):891-895.

91. Ornstein P, Cherepon JA. Demographic variables as predictors of alcoholism treatment outcome. *JSA.* Sep 1985;46(5):425-432.

92. Willems S, De Maesschalck S, Deveugele M, Derese A, De Maeseneer J. Socio-economic status of the patient and doctor-patient communication: does it make a difference? *Patient Educ Couns.* Feb 2005;56(2):139-146.

93. Street RL, Jr., Gordon HS, Ward MM, Krupat E, Kravitz RL. Patient participation in medical consultations: why some patients are more involved than others. *Med Care.* Oct 2005;43(10):960-969.

94. Kaplan SH, Gandek B, Greenfield S, Rogers W, Ware JE. Patient and visit characteristics related to physicians' participatory decision-making style. Results from the Medical Outcomes Study. *Med Care.* Dec 1995;33(12):1176-1187.

95. Nease RF, Jr., Brooks WB. Patient desire for information and decision making in health care decisions: the Autonomy Preference Index and the Health Opinion Survey. *J Gen Intern Med.* Nov 1995;10(11):593-600.

96. Cooper LA, Gonzales JJ, Gallo JJ, et al. The acceptability of treatment for depression among African-American, Hispanic, and white primary care patients. *Med Care.* 2003;41(4):Apr.

97. Cooper-Patrick L, Gallo JJ, Powe NR, Steinwachs DM, Eaton WW, Ford DE. Mental health service utilization by African Americans and Whites: the Baltimore Epidemiologic Catchment Area Follow-Up. *Med Care.* Oct 1999;37(10):1034-1045.

98. Johnson RL, Roter D, Powe NR, Cooper LA. Patient race/ethnicity and quality of patient-physician communication during medical visits. *Am J Public Health.* Dec 2004;94(12):2084-2090.

99. Street RL, Jr. Gender differences in health care provider-patient communication: are they due to style, stereotypes, or accommodation? *Patient Educ Couns.* Dec 2002;48(3):201-206.

100. Dawson DA, Grant BF. Gender effects in diagnosing alcohol abuse and dependence. *J Clin Psychol.* Mar 1993;49(2):298-307.

101. Booth BM, McLaughlin YS. Barriers to and need for alcohol services for women in rural populations. *Alcohol Clin Exp Res.* 2000;24(8):1267-1275.

102. Institute of Medicine. *Report of the Conference on "Assessing Future Research Needs: Mental and Addictive Disorders in Women".* Washington, DC: National Academy of Sciences; 1991.

103. Roman PM. *Women and Alcohol Use: A Review of the Research Literature.* Washington, DC: U.S. Dept. of Health and Human Services 1988. Pub (ADM) 88-1574.

104. Roman PM. Biological features of women's alcohol use: a review. *Public Health Rep.* 1988;103(6):628-637.

105. Hser YI, Anglin MD, McGlothlin W. Sex differences in addict careers. 1. Initiation of use. *Am J Drug Alcohol Abuse.* 1987;13(1-2):33-57.

106. Hser YI, Anglin MD, Booth MW. Sex differences in addict careers. 3. Addiction. *Am J Drug Alcohol Abuse.* 1987;13(3):231-251.

107. Anglin MD, Hser YI, Booth MW. Sex differences in addict careers. 4. Treatment. *Am J Drug Alcohol Abuse.* 1987;13(3):253-280.

108. Marsh JC, Cao D, D'Aunno T. Gender differences in the impact of comprehensive services in substance abuse treatment. *J Subst Abuse Treat.* Dec 2004;27(4):289-300.

109. Parthasarathy S, Weisner C. Five-year trajectories of health care utilization and cost in a drug and alcohol treatment sample. *Drug Alcohol Depend.* Nov 1 2005;80(2):231-240.

110. Clayton RR. Multiple drug use. Epidemiology, correlates, and consequences. *Recent Dev Alcohol.* 1986;4:7-38.

111. Kania J, Kofoed L. Drug use by alcoholics in outpatient treatment. *Am J Drug Alcohol Abuse.* 1984;10(4):529-534.

112. Hubbard RL. Treating combined alcohol and drug abuse in community-based programs. *Recent Dev Alcohol.* 1990;8:273-283.

113. Weisner C. The merging of alcohol and drug treatment: a policy review. *J Public Health Policy.* Spring 1992;13(1):66-80.

114. Moos RH, Mertens JR, Brennan PL. Patterns of diagnosis and treatment among late- middle-aged and older substance abuse patients. *JSA.* Jul 1993;54(4):479-487.

115. Tam TW, Weisner C, Mertens J. Demographic characteristics, life context, and patterns of substance use among alcohol dependent treatment clients in a health maintenance organization. *Alcohol Clin Exp Res.* Dec 2000;24(12):1803-1810.

116. Satre DD, Mertens J, Arean PA, Weisner C. Contrasting outcomes of older versus middle-aged and younger adult chemical dependency patients in a managed care program. *JSA.* Jul 2003;64(4):520-530.

117. Liberto JG, Oslin DW, Ruskin PE. Alcoholism in older persons: A review of the literature. *Hosp Community Psychiatry.* 1992;43:975-984.

118. Oslin DW. Alcohol use in late life: disability and comorbidity. *J Geriatr Psychiatry Neurol.* Fall 2000;13(3):134-140.

119. Mertens JR, Weisner CM. Predictors of substance abuse treatment retention among women and men in an HMO. *Alcoholism: Clinical and Experimental Research.* Oct 2000;24(10):1525-1533.

120. Lemke S, Moos RH. Prognosis of older patients in mixed-age alcoholism treatment programs. *J Subst Abuse Treat.* 2002;22(1):33-43.

121. Oslin DW, Pettinati H, Volpicelli JR. Alcoholism treatment adherence: older age predicts better adherence and drinking outcomes. *Am J Geriatr Psychiatry.* Nov-Dec 2002;10(6):740-747.

122. Satre DD, Blow FC, Chi FW, Weisner C. Gender differences in seven-year alcohol and drug treatment outcomes among older adults *American Journal on Addictions.* May-Jun 2007;16(3):216-221.

123. Kashner TM, Rodell DE, Ogden SR, Guggenheim FG, Karson CN. Outcomes and costs of two VA inpatient treatment programs for older alcoholic patients. *Hosp Community Psychiatry.* Oct 1992;43(10):985-989.

124. Rice C, Longabaugh R, Beattie M, Noel NE. Age group differences in response to treatment for problematic alcohol involvement. *Addiction.* Oct 1993;88(10):1369-1375.

125. Gfroerer J, Penne M, Pemberton M, Folsom R. Substance abuse treatment need among older adults in 2020: the impact of the aging baby-boom cohort. *Drug Alcohol Depend.* Mar 1 2003;69(2):127-135.

126. Weisner C, Matzger H, Tam T, Schmidt L. Who goes to alcohol and drug treatment? Understanding utilization within the context of insurance. *JSA.* Nov 2002;63(6):673-682.

127. Zeber JE, Copeland LA, Grazier KL. Serious mental illness, aging, and utilization patterns among veterans. *Mil Med.* Jul 2006;171(7):619-626.

128. Satre DD, Knight BG, David S. Cognitive behavioral interventions with older adults: Integrating clinical and gerontological research. *Professional Psychology: Research and Practice.* Oct 2006;37(5):489-498.

129. Hassan I, McCabe R, Priebe S. Professional-patient communication in the treatment of mental illness: a review. *Commun Med.* 2007;4(2):141-152.

130. Blow FC, (Consensus Panel Chair). *Substance Abuse Among Older Adults. Treatment Improvement Series Protocol (TIP) Series 26*. Rockville, MD: Center for Substance Abuse Treatment, Substance Abuse and Mental Health Services Administration. DHHS Publication No. (SMA) 98-3179; 1998.

131. Holder HD, Blose JO. The reduction of health care costs associated with alcoholism treatment: a 14-year longitudinal study. *JSA.* Jul 1992;53(4):293-302.

132. Holder HD, Lennox RD, Blose JO. The economic benefits of alcoholism treatment: A summary of twenty years of research. *J Employ Assist Res* 1992;1(1):63-82.

133. Stein MD, O'Sullivan PS, Ellis P, Perrin H, Wartenberg A. Utilization of medical services by drug abusers in detoxification. *J Subst Abuse.* 1993;5(2):187-193.

134. Holder HD, Hallan JB. Impact of alcoholism treatment on total health care costs: a six-year study. *Adv Alcohol Subst Abuse.* Fall 1986;6(1):1-15.

135. Jones K, Vischi T. Impact of alcohol, drug abuse, and mental health treatment in medical care utilization: a review of the research literature. *Med Care.* 1979;17 (Supplement)(12):1-82.

136. Saxe L, Dougherty D, Esty K, Fine M. *The effectiveness and costs of alcoholism treatment (Health Technology Case Study 22)*. Washington, DC: Office of Technology Assessment; 1983.

137. Smart RG, Mann RE, Adrian M. Health and productivity savings from increased alcoholism treatment in Ontario. *Canadian Journal of Public Health.* 1993;84(1):62-63.

138. Rice DP, Kelman S, Miller LS. Estimates of economic costs of alcohol and drug abuse and mental illness, 1985 and 1988. *Public Health Reports.* 1991;106(3):280-291.

139. Parthasarathy S, Weisner C, Hu TW, Moore C. Association of outpatient alcohol and drug treatment with health care utilization and cost: revisiting the offset hypothesis. *JSA.* Jan 2001;62(1):89-97.

140. Rice DP, Kelman S. Measuring comorbidity and overlap in the hospitalization cost for alcohol and drug abuse and mental illness. *Inquiry.* 1989;26(2):249-260.

141. Weintraub E, Dixon L, Delahanty J, et al. Reason for medical hospitalization among adult alcohol and drug abusers. *Am J Addict.* 2001;10(2):167-177.

142. Jerrell JM, Wieduwilt KM, Macey DV. Use and costs of public-sector behavioral health services for African-American and White women. *Psychiatr Serv.* 2002;53(2):195-200.

143. Fleming MF, Mundt MP, French MT, Manwell LB, Stauffacher EA, Barry KL. Benefit-cost analysis of brief physician advice with problem drinkers in primary care settings. *Med Care.* 2000;38(1):7-18.

144. Fleming MF, Graham AW. Screening and brief interventions for alcohol use disorders in managed care settings. *Recent Dev Alcohol.* 2001;15:393-416.

145. Fleming MF, Mundt MP, French MT, Manwell LB, Stauffacher EA, Barry KL. Brief physician advice for problem drinkers: Long-term efficacy and benefit-cost analysis. *Alcoholism: Clinical and Experimental Research.* 2002;26(1):36-43.

146. Sterling S, Chi F, Weisner C, Campbell CI. Three-year substance use and mental health outcomes among adolescents entering chemical dependency treatment: the role of continuing care. *Addiction Health Services Research Conference*. Athens, GA; 2007.

147. Satre DD, Mertens JR, Arean PA, Weisner C. Five-year alcohol and drug treatment outcomes of older adults versus middle-aged and younger adults in a managed care program. *Addiction.* Oct

2004;99(10):1286-1297.
148. Mendelson DN, Schwartz WB. The effects of aging and population growth on health care costs. *Health Aff (Millwood).* Spring 1993;12(1):119-125.
149. Polder JJ, Bonneux L, Meerding WJ, van der Maas PJ. Age-specific increases in health care costs. *Eur J Public Health.* Mar 2002;12(1):57-62.
150. Seshamani M, Gray A. Health care expenditures and ageing: an international comparison. *Appl Health Econ Health Policy.* 2003;2(1):9-16.
151. Samet JH, Friedmann P, Saitz R. Benefits of linking primary medical care and substance abuse services: patient, provider, and societal perspectives. *Arch Intern Med.* Jan 8 2001;161(1):85-91.
152. Gordon AJ, Fiellin DA, Friedmann PD, et al. Update in addiction medicine for the primary care clinician. *J Gen Intern Med.* Oct 2 2008.
153. Gordon AJ, Sullivan LE, Alford DP, et al. Update in addiction medicine for the generalist. *J Gen Intern Med.* Aug 2007;22(8):1190-1194.
154. Curley B. Positive prognosis for addiction treatment in healthcare reform *Join Together*; 2009.
155. Sellers EM, Ciraulo DA, DuPont RL, et al. Alprazolam and benzodiazepine dependence. *J Clin Psychiatry.* Oct 1993;54 Suppl:64-75; discussion 76-67.
156. Drug Enforcement Administration, Office of Diversion Control. State Prescription Drug Monitoring Programs. http://www.deadiversion.usdoj.gov/faq/rx_monitor.htm.
157. Mechanic RE. Disease management: a promising approach for health care purchasers. *Issue Brief*; 2002.
158. McCarty D, McConnell KJ, Schmidt LA. Priorities for policy research on treatments for alcohol and drug use disorders. *Annual Grantee Meeting of the Robert Wood Johnson Substance Abuse Policy Research Program*. Tucson, AZ; 2008.
159. Samet JH. An internist encounters addiction: heroin, alcohol, and HIV Infection in a 42-year-old man with abdominal pain. *Contemporary Clinical Medicine-Great Teachers Grand Rounds, NIH Clinical Center*. Bethesda, MD; 2005.
160. Meresman JF, Hunkeler EM, Hargreaves WA, et al. A case report: implementing a nurse telecare program for treating depression in primary care. *Psychiatr Q.* Spring 2003;74(1):61-73.
161. Sterling S, Weisner C. Translating research findings into practice: example of treatment services for adolescents in managed care. *Alcohol Research & Health.* 2006;29(1):11-18.
162. Chi FW, Weisner CM. Nine-year psychiatric trajectories and substance use outcomes: an application of the group-based modeling approach. *Eval Rev.* Feb 2008;32(1):39-58.
163. Chi FW, Satre DD, Weisner C. Chemical dependency patients with cooccurring psychiatric diagnoses: service patterns and 1-year outcomes. *Alcoholism: Clinical and Experimental Research.* May 2006;30(5):851-859.
164. Sterling S, Weisner C. Chemical dependency and psychiatric services for adolescents in private managed care: Implications for outcomes. *Alcoholism: Clinical and Experimental Research.* May 2005;25(5):801-809.
165. Sterling S, Kohn CS, Lu Y, Weisner C. Pathways to chemical dependency treatment for adolescents in an HMO. *J Psychoactive Drugs.* Dec 2004;36(4):439-453.
166. Sterling S, Chi FW, Campbell CI, Weisner C. Three-year chemical dependency and mental health treatment outcomes among adolescents: the role of continuing care. *Alcoholism: Clinical and Experimental Research.* Aug 2009;33(8):1417-1429.
167. Mertens JR, Flisher AJ, Fleming MF, Weisner CM. Medical conditions of adolescents in alcohol and drug treatment: comparison with matched controls. *J Adolesc Health.* Feb 2007;40(2):173-179.
168. Campbell CI, Chi FW, Sterling S, Kohn CS, Weisner C. Self-initiated tobacco cessation and substance use outcomes among adolescents entering substance use treatment in a managed care organization. *Addict Behav.* Feb 2009;34(2):171-179.
169. Mertens JR, Weisner C, Ray GT, Fireman B, Walsh K. Hazardous drinkers and drug users in HMO primary care: Prevalence, medical conditions, and costs. *Alcohol Clin Exp Res.* Jun 2005;29(6):989-998.
170. Mertens J, Weisner C, Sterling S. Disparities across treatment settings for the medically indigent: implications for substance abuse screening and interventions. *FrontLines.* 2001;June:6;8.
171. Ward CL, Mertens JR, Flisher AJ, et al. Substance abuse and HIV risk behaviours in Cape Town primary health care service users. *South African Psychiatry Review.* Nov 2005;8:160-165.
172. Ward CL, Mertens JR, Flisher AJ, et al. Prevalence and correlates of substance use among South

african primary care clinic patients. *Subst Use Misuse.* 2008;43(10):1395-1410.

173. Avalos LA, Mertens J, Ward CL, Flisher AJ, Bresick GF, Weisner CM. Stressors, substance use and sexual risk behaviors among primary care patients in Cape Town, South Africa. *AIDS and Behavior.* 2009;14(2):359-370.

174. Mertens JR, Ward CL, Flisher AJ, Bresick GF, Sterling S, Weisner C. Development of a brief alcohol and drug screener in public sector Cape Town primary health care clinics. *10th annual meeting of the International Society of Addiction Medicine*. Cape Town, South Africa; 2008.

175. Mertens JR. *Medical conditions in individuals with alcohol and drug problems: prevalence and influence on long-term treatment outcome.* [dissertation]. Cape Town, South Africa: Department of Psychiatry and Mental Health, University of Cape Town; 2006.

176. Mertens J. Substance use and HIV risk in public sector Cape Town primary care clinics: Results and next steps. *Center for Health Care Evaluation*; 2007.

177. Mertens JR, Flisher AJ, Ward CL, Bresick GF, Sterling S, Weisner C. Medical conditions of hazardous drinkers and drug users in primary care clinics in Cape Town, South Africa. *Journal of Drug Issues.* Fall 2009;39(4):889-1014.

178. Weisner C, Hinman A, Lu YW, Mertens JR, Moore C, Monahan J. Alcohol and drug treatment in the context of health reform in the U.S.: the thorny example of mandated treatment. *J Subst Abuse Treat.* under review.

179. Weisner C, Matzger H. Missed opportunities in screening for alcohol problems in medical and mental health services. *Alcoholism: Clinical and Experimental Research.* Jul 2003;27(7):1132-1141.

180. Weisner C, Delucchi K, Matzger H, Schmidt L. The role of community services and informal support on five-year drinking trajectories of alcohol dependent and problem drinkers. *JSA.* Nov 2003;64(6):862-873.

181. Parthasarathy S, Mertens J, Moore C, Weisner C. Utilization and cost impact of integrating substance abuse treatment and primary care. *Med Care.* Mar 2003;41(3):357-367.

182. Rice DP, Conell C, Weisner C, Hunkeler EM, Fireman B, Hu TW. Alcohol drinking patterns and medical care use in an HMO setting. *J Behav Health Serv Res.* Feb 2000;27(1):3-16.

183. Hunkeler EM, Hung YY, Rice DP, Weisner C, Hu T-W. Alcohol consumption patterns and health care costs in an HMO. *Drug Alcohol Depend.* Oct 1 2001;64(2):181-190.

184. Hunkeler EM, Spector WD, Fireman B, Rice DP, Weisner C. Psychiatric symptoms, impaired function, and medical care costs in an HMO setting. *Gen Hosp Psychiatry.* May-Jun 2003;25(3):178-184.

185. Mertens J, Weisner C, Moore C, Parthasarathy S. Identification of substance abuse disorders in an HMO: Do medical or psychiatric comorbidities make a difference? Chicago, IL: 127th annual meeting of the American Public Health Association; 1999.

186. Weisner C, Lu Y, Hinman A, et al. Substance use, symptom, and employment outcomes of persons with a workplace mandate for chemical dependency treatment. *Psychiatr Serv.* May 2009;60(5):646-654.

187. Schmidt L, Weisner C. Public health perspectives on access and need for substance abuse treatment. In: Tucker JA, Donovan DM, Marlatt GA, eds. *Changing Addictive Behavior: Bridging Clinical and Public Health Strategies.* New York: Guildford Press; 1999:67- 96.

188. Weisner C. Alcohol health services research: an evolving agenda. *Alcohol Health and Research World.* 1995;19(1):71-76.

189. Polcin DL, Weisner C. Factors associated with coercion in entering treatment for alcohol problems. *Drug Alcohol Depend.* Mar 1 1999;54(1):63-68.

190. Weisner C. Coercion in alcohol treatment. In: Institute of Medicine, ed. *Broadening the Base of Treatment for Alcohol Problems*. Washington, DC: National Academy Press; 1990:579-609.

191. Weisner C, Greenfield TK, Room R. Trends in the treatment of alcohol problems in the U.S. general population, 1979 through 1990. *American Journal of Public Health.* Jan 1995;85(1):55-60.

192. Kaskutas LA, Weisner C, Caetano R. Predictors of help seeking among a longitudinal sample of the general population, 1984-1992. *JSA.* Mar 1997;58(2):155-161.

193. Weisner C. The epidemiology of combined alcohol and drug use within treatment agencies: a comparison by gender. *JSA.* May 1993;54(3):268-274.

194. Weisner C. The role of alcohol-related problematic events in treatment entry. *Drug Alcohol Depend.* Oct 1990;26(2):93-102.

195. Weisner C, Mertens J, Tam T, Moore C. Factors affecting the initiation of substance abuse treatment in managed care. *Addiction.* May 2001;96(5):705-716.

196. Weisner C, Matzger H. A prospective study of the factors influencing entry to alcohol and drug

treatment. *J Behav Health Serv Res.* May 2002;29(2):126-137.
197. Mertens JR, Weisner C, Ray GT. Readmission among chemical dependency patients in private, outpatient treatment: Patterns, correlates and role in long-term outcome. *JSA.* Nov 2005;66(6):842-847.
198. Weisner C, Mertens J, Parthasarathy S, et al. The outcome and cost of alcohol and drug treatment in an HMO: day hospital versus traditional outpatient regimens. *Health Serv Res.* Oct 2000;35(4):791-812.
199. Ray GT, Weisner CM, Mertens JR. Relationship between use of psychiatric services and five-year alcohol and drug treatment outcomes. *Psychiatr Serv.* Feb 2005;56(2):164-171.
200. Campbell CI, Weisner C, Sterling S. Adolescents entering chemical dependency treatment in private managed care: ethnic differences in treatment initiation and retention. *Journal of Adolescent Health.* Apr 2006;38(4):343-350.
201. Sterling S, Campbell CI, Weisner C, Lu Y, Chi FW, Duhe J. African American adolescents in chemical dependency treatment in managed care: risk factors, services utilization and outcomes. *Joint Meeting on Adolescent Treatment Effectiveness (JMATE).* Washington, DC; 2008.
202. Bryk AS, Raudenbush SW. *Hierarchical Linear Models: Applications and Data Analysis Methods.* Newbury Park: Sage; 1992.
203. Hser YI, Shen H, Chou CP, Messer SC, Anglin MD. Analytic approaches for assessing long-term treatment effects. Examples of empirical applications and findings. *Eval Rev.* 2001;25(2):233-262.
204. Beacon HJ, Thompson SG. Multi-level models for repeated measurement data: application to quality of life data in clinical trials. *Stat Med.* 1996;15(24):2717-2732.

If you have a separate bibliography, attach it to the submission with your other study documents.

13.0 Sample Size and Eligibility

13.1 Number of subjects that will be enrolled at UCSF and affiliated institutions:

700

13.2 Total number of subjects that will be enrolled at all sites (Help Text updated 9/13):

700

13.3 Estimated number of people that you will need to consent and screen here (but not necessarily enroll) to get the needed subjects:

824

13.4 Explain how and why the number of subjects was chosen (Help Text updated 9/13):

Power Analyses for Primary Hypotheses. Sample size estimates were based on two sources. The first was the abstinence rates observed in our NIDA Integrated Care Study (which randomized KP drug treatment patients to integrated PC and drug treatment within the drug program vs. PC as usual during drug abuse treatment[30] (abstinence rates of 69% vs. 55% for the groups of interest at 6 months). Other of our studies have found similar results.[198] Because the intervention is expected to promote more ongoing PC visits which are expected to relate to abstinence, we expect that the difference in abstinence rates will not diminish over time. Based on this and other studies within KP CDRP facilities, we conservatively expect an 85% recruitment rate. The intervention is not one which patients might find threatening or inconvenient. On the contrary, as we found in the Integrated Care Study, patients responded positively to having assistance in establishing PC contacts and selecting a PC physician. Based on our observational continuing care analyses, we expect that the Continuing Care arm would have a mean change at 12 months of 2 PC (SE=3.2) visits vs. .5 (SE=.9) for Usual Care; at 24 months: 2 PC (SE=3.1) visits vs. .5 (.8) for Usual Care. In regard to CDRP readmissions, we found in our earlier studies that of those readmitted to drug treatment over 5 years (N=443), 72% were readmitted in the first year after index treatment. A total of 27% of the original sample were readmitted between index treatment and one year after intake; 7.3% in the second year after

intake. We examined KP administrative data on program intake and retention rates for the San Francisco CDRP for one year: 702 patients completed 1 week of treatment. Thus, recruiting for 20 months will provide a sufficient number of intakes, as well as individuals who we expect will complete the entire intervention (two intervention sessions and linkage conference call) (80%). We will be able to attempt to recruit 824 patients to have an estimated sample size of 700 consented participants. Individuals will be included in the ongoing study regardless of whether they continue in, or complete, drug treatment or not, and we will examine whether it is effective for those who do not complete treatment. (The model is designed to integrate patients to PC early to treatment as a facilitator for CDRP readmission if needed in the case of treatment drop-out or failure.)

13.5 * Eligible age range(s):

☐ 0-6 years
☐ 7-12 years
☐ 13-17 years
☑ 18+ years

13.6 Inclusion criteria:

Research participants will be adult patients at the San Francisco CDRP, enrolled in either DayHospitalor Outpatient who have completed 1 week of treatment.

13.7 Exclusion criteria:

Patients with a diagnosis of dementia, mental retardation, or who are actively psychotic or suicidal will be excluded by their clinician.

13.8 There are inclusion or exclusion criteria based on gender, race or ethnicity:

○ Yes ◉ No

If
yes
, please explain the nature and rationale for the restrictions:

14.0 Other Approvals and Registrations

14.1 * Do any study activities take place on patient care units:

○ Yes ◉ No

If **Yes**, attach a letter of support for the study from the involved patient care manager(s).

14.2 * Does your protocol involve any radiation exposure to patients/subjects? The UCSF Radiation Safety Committee requires review of your protocol if it includes administration of radiation as part of standard of care OR research exposures:

○ Yes ◉ No

14.3 * This study may generate genetic data that may be broadly shared (e.g. submitted to NIH for Genome-Wide Association Studies (GWAS) in dbGaP, TCGA, etc):

○ Yes ◉ No

14.4 * This study involves administration of vaccines produced using recombinant DNA technologies to human subjects:

○ Yes ◉ No

14.5 * This study involves human gene transfer (NOTE: Requires NIH Recombinant DNA Advisory Committee (RAC) review prior to CHR approval):

○ Yes ◉ No

14.6 This study involves other regulated materials and requires approval and/or authorization from the following regulatory committees:

☐ Institutional Biological Safety Committee (IBC)

Specify BUA #:

☐ Institutional Animal Care and Use Committee (IACUC)

Specify IACUC #:

☐ Radiation Safety Committee

Specify RUA #:

☐ Radioactive Drug Research Committee (RDRC)

Specify RDRC #:

☐ Controlled Substances

15.0 Procedures

15.1 * Procedures/Methods (Help Text updated 9/13) For clinical research list all study procedures, test and treatments required for this study, including when and how often they will be performed. If there are no clinical procedures, describe the Methods:

Usual Care: The Usual Care medical education that is part of standard CDRP treatment consists of 2 sessions (1.5 hours each) which include education regarding health and drug use (i.e., which medical conditions are exacerbated by alcohol and drug use; adverse alcohol, drug and medication interactions). Usual Care also includes all of the individual and group counseling components of the CDRP program. *The Usual Care group will also receive information and instruction on how to register and use "kp.org" (see below).*

Continuing Care: The intervention's goal is to help patients understand the importance of their overall health care and to activate them to engage in healthy practices and take a proactive role with their PC physician in their health care. Its goal is also to provide a PC linkage which will enlist their physician as an ongoing ally in their long term recovery. During the 2 weeks after randomization, in addition to Usual Care, Continuing Care patients will receive:

1) two group sessions on health and physician communication interaction and
2) a linkage phone call with the PC physician and therapist.

Please note: Study participants will be consented separately for this activity, following their participation in the patient activation groups. The consent process for this activity will include an explanation that their Primary Care Provider is likely to document this conversation in their medical record, and as a result, their participation in Chemical Dependency Treatment will no longer be protected by the stricter confidentiality standards provided by the information "firewall" as required by 42 CFR. It will be explained that there will thus be an increased risk of loss of confidentiality related to the Primary Care Linkage Call of allowing CD treatment to be included in their medical record, as a result of the conversation with their Primary Care Provider, and that, if documented in their regular medical record by their Primary Care Provider, CD treatment would also no longer be protected by law from disclosure by subpoena. (please see Addendum Consent Form)

The intervention is based on activating patients but also orienting PC physicians. Thus, the design includes a 1-hour informational session for PC physicians during a lunch staff meeting to inform them of the linkage, information on where to find guidelines in the EMR, and an EMR alert reminder to the PC Physician before the patient's visit. The informational session will not instruct providers to increase drug and alcohol screening or to alter their usual treatment of patients. Please note:
Primary care providers are not research subjects in the study. No analysis will be done evaluating their

performance, or at the physician level at all. Similar to other quality improvement innovations made in primary care, they will be informed (during a regular lunch hour staff meeting) that a consultation appointment will be made in their schedules to have a phone call with the chemical dependency therapist and the patient (who is one of their patients). In Kaiser, physicians are commonly informed of system changes to increase clinical quality improvement in this manner, and telephone consultation appointments with other Kaiser clinicians are also common. The primary care providers are not subjects in the study. The intervention/training and study participation is at the level of patients – they are the ones being trained in how to use primary care.
The aims of the patient activation groups are:
a. Continuing Care Intervention Session 1 - to help understand the role of health care and to activate patients in becoming aware of and addressing their overall health care context, and viewing their substance use problem as a chronic medical condition that can benefit from ongoing PC monitoring; and
b. Continuing Care Intervention Session 2 - to teach how to identify and access services and communicate with their PC physician.
Intervention Study participants will complete a computer-based interview in a private place at the CDRP at baseline, using the same user-friendly technology we have previously used for NIDA studies in the programs. Follow-up interviews will be conducted at 6, 12, and 24 months, and at 5 years by telephone by research staff from the Division of Research. A random sub-sample is flagged for the interviewer to recruit for an in-person visit for urinalysis. We will follow the same protocol used in prior NIDA KP studies. At baseline and follow-ups, participants will be asked for information to facilitate tracking, (address, home and alternate phone numbers, and the names, addresses and phone numbers of 3 or 4 others who would always know of their whereabouts). A toll-free number is provided for calling the study if contact information changes. (In addition, KP members' telephone numbers and addresses are updated at each medical visit, advice call, and when appointments are made). Our toll-free call-in line is staffed with trained interviewers during designated hours to allow participants to call at their convenience to complete follow-up interviews. This number is disseminated to respondents and contacts and is the number left on answering machines. Scripts for messages left for respondents or contacts are prepared that do not compromise confidentiality. When our phone calls are not responded to by the subject, we will pursue all collateral contacts. When these contacts fail to provide a successful connection to the respondent, address confirmation is attempted through voter registration, Social Security and criminal justice public domain information, credit bureaus, directory services, Internet searches, and the messaging service of the Department of Motor Vehicles.
We will conduct follow-up interviews whether or not patients remain KP members. Upon being contacted for a follow-up, participants will be reminded of the study and permission to conduct the follow-up interview will be obtained verbally. Because about 20% of the sample is expected to have left KP by the 2-year follow up, we will include self report of utilization questions, as we have in our prior studies. In addition to medical visits, we will also examine patient activation scores and ask those who have left KP about health behaviors to assess whether patient activation has been transferred to other settings. Follow-up data will be collected by telephone by interviewers using a highly efficient computerized system also used in our earlier studies. Interviewers will be supervised by the Project Coordinator, Sterling, with consultation from Weisner.
Urinalysis at Follow-up
In order to conduct urinalysis at follow-up of a random sub-sample, the group of interest will be identified and flagged for the interviewer to recruit. Following the phone interview, these individuals will be requested to present at the CDRP within the following 5 days to take a urine test and a brief interview. Patients are given an appointment time at their convenience (including evenings). When they arrive at the CDRP, the research staff meets them at a separate registration counter. They do not need to register at the CDRP. Patients sign informed consent, complete a brief questionnaire which includes substance use questions comparable with those on the telephone interview, and provide a urine test. The results of these tests are not included in their medical record. This procedure has been used in our other NIDA studies and has good compliance.

If you have a procedure table, attach it to the submission with your other study documents.

15.2 Interviews, questionnaires, and/or surveys will be administered or focus groups will be conducted:

(●) Yes ( ) No

List any standard instruments used for this study:

Attach any non-standard instruments at the end of the application.

15.3 Conduct of study procedures or tests off-site by non-UCSF personnel:

(•) Yes ( ) No

If yes, explain:

This study is being conducted in conjunction with Kaiser Permanente Northern CA. KP staff will conduct study procedures at the Kaiser Permanente San Francisco CDRP. Please see section 18.1 for detailed procedural information.

15.4 Sharing of experimental research test results with subjects or their care providers:

( ) Yes (•) No

If yes, explain:

15.5 * Specimen collection for future research and/or specimen repository/bank administration:

( ) Yes (•) No

15.6 Time commitment (per visit and in total):

Usual Care

- The Usual Care medical education that is part of standard CDRP treatment consists of 2 education sessions (1.5 hours each) regarding health and drug use (i.e., which medical conditions are exacerbated by alcohol and drug use; adverse alcohol, drug and medication interactions). Usual Care also includes all of the individual and group counseling components of the CDRP program. The Usual Care group will also receive information and instruction on how to register and use “kp.org”
- Follow-up interviews at 6, 12, and 24 months and at 5 years (1/2 hour each)

Continuing Care During the 2 weeks after randomization, in addition to Usual Care, Continuing Care patients will receive:

- Two group sessions on health and physician communication interaction (1.5 hours each)
- Follow-up interviews at 6,12 and 24 months, and at 5 years (1/2 hour each)
- A linkage phone call with the PC physician and therapist (10 minutes)

Physicians

One “linkage” phone call = 10 minutes. One informational session = one hour

15.7 Locations:

Kaiser Permanente Medical Center CDRP, San Francisco, CA
Kaiser Permanente Division of Research, Oakland, CA
Kaiser Permanente Medical Center, South San Francisco, CA

15.8 Describe the resources in place to conduct this study in a way that assures protection of the rights and welfare of participants:

SF KP Chemical Dependency Recovery Program (CDRP) usual care service from which participants will be recruited includes both outpatient and day hospital treatment regimens, which run concurrently. Services in both are the same, but day hospital has greater intensity of each type of service. For both, services include supportive group therapy, education, relapse prevention, family therapy, and 12-step groups. Also for both, patients are assigned a primary therapist, and individual counseling is available as needed. Drug screening tests are scheduled randomly once per week during the first 2 weeks and monthly during the remainder of treatment. In both, the rehabilitation part of treatment is 2 weeks, followed by another 6 weeks of less intensive treatment (similar to the outpatient program as described below). Aftercare is available for 10

months. The outpatient program meets 3 days/week for 1-1/2 hours/day for 8 weeks.
Staff include 3 (2.5 FTE) physicians (1 internist and 2 psychiatrists), 3 (2.5 FTE) nurse practitioners, 1 licensed vocational nurse, 20 licensed licensed PhD/masters-level clinicians (social workers and psychologists), and 3 California licensed drug and alcohol counselors (CADACs). KP data systems (see below) provide information on the provider and type of service within the CDRP.
Patients admitted to the CDRP day treatment program will be invited to participate in the study. Patients who are under age 18, who have a diagnosis of mental retardation, dementia, or psychosis, as assessed by intake clinicians and who did not have a permanent residence in the San Francisco Bay Area will not be recruited. The study will be described to those eligible. They will be informed that participation is voluntary and that nonparticipation will not affect receiving services from the program. The person interviewing the patient at admission will be an independent Division of Research staff member and not someone who would be involved in his or her treatment or have decision making over this person. Patients agreeing to participate will be asked to sign a statement of informed consent. Informed consent will also cover the examination of medical records data. If the subject cannot read, the interviewer will read the form to him/her. Subjects may refuse to answer any of the specific questions in the interviews, can terminate interviews at any time, or refuse participation in any part of the project with which they feel uncomfortable. They will be given a name and telephone number of the Project Director and Principal Investigator and told they can contact them if they have any further questions.
Importantly, all patients who agree to participate in the study also will receive standard substance abuse treatment, and no usual component of care will be withheld. Standard at the CDRP is divided into two stages: the Rehabilitative stage (8 weeks) and the Aftercare stage (10 months). Upon completion of the initial 8 weeks of treatment, study patients of both treatment programs will be referred to the aftercare program.

16.0 Alternatives

16.1 Study drug or treatment is available off-study:

○ Yes
○ No
◉ Not applicable

16.2 * Is there a standard of care (SOC) or usual care that would be offered to prospective subjects at UCSF (or the study site) if they did not participate:

○ Yes ◉ No

If yes, describe the SOC or usual care that patients would receive if they choose not to participate:

SF KP Chemical Dependency Recovery Program (CDRP) usual care includes both outpatient and day hospital treatment regimens, which run concurrently. Services in both are the same, but day hospital has greater intensity of each type of service. For both, services include supportive group therapy, education, relapse prevention, family therapy, and 12-step groups. Also for both, patients are assigned a primary therapist, and individual counseling is available as needed. Drug screening tests are scheduled randomly once per week during the first 2 weeks and monthly during the remainder of treatment. In both, the rehabilitation part of treatment is 2 weeks, followed by another 6 weeks of less intensive treatment (similar to the outpatient program as described below). Aftercare is available for 10 months. The outpatient program meets 3 days/week for 1.5 hours/day for 8 weeks.

16.3 Describe other alternatives to study participation that are available to prospective subjects:

Participation in the study is completely voluntary. Patients may choose not to participate in the study. Participation or non-participation will have no impact on the patients' medical care.

17.0 Risks and Benefits

17.1 * Risks and discomforts:

Because materials for the proposed study entail disclosure about sensitive information, there are risks to confidentiality in the proposed study. Participants may feel uncomfortable disclosing information about drug and alcohol use behavior, medical or psychiatric symptoms. Although both treatment arms are low risk, it is possible that those assigned to Usual Care may experience less treatment improvement than those assigned the experimental intervention. However, both study groups will receive usual care, and the experimental treatment in this study (Continuing Care) is considered a low-risk treatment method.

17.2 Steps taken to minimize risks to subjects:

The following steps will be followed to minimize the risk to participant confidentiality: All data will be identified only with participant numbers, and never with names or initials. All data will be stored in locked cabinets in offices at the Kaiser Division of Research (DOR) in Oakland, CA. The list associating names and participant numbers will be stored in a separate locked cabinet, and only the PI will have access to the key. Research staff will not release any information to anyone other than the participant without the participant's written consent.
To minimize the risk of participant discomfort in the disclosure of sensitive information, confidentiality of all study materials will be emphasized during participant interviews. Participants will be informed that they have the right to refuse to answer questions or to terminate their participation in the study at any time.
The experimental treatment protocol (Continuing Care) is a low-risk supplemental intervention that will be conducted at the same treatment location where patients receive usual CDRP care. These participants will continue to receive all the components of standard outpatient CDRP services, in addition to the experimental intervention. The PI will carefully monitor delivery of the intervention and will take all steps to ensure participant safety. Safety will be monitored through the oversight of the Center's Data Safety and Monitoring Plan). Finally, participants receiving usual care will receive the same care they would have received if they were not part of the study.

17.3 Benefits to subjects:

(•) Yes ( ) No

If yes, describe:

It is not known if participants will receive a direct benefit from the study. The potential benefits for the study participants include decreased drug and alcohol use, improved functioning and quality of life.

17.4 Benefits to society:

Study of the effectiveness of the intervention will also have benefits for society by providing information about how best to treat patients with drug or alcohol abuse, including better understanding of chronic care treatment models and coordination of drug and alcohol abuse services with health care. The proposed research will help meet current and future treatment demand. Drug and alcohol abuse affects a large percentage of individuals. This study takes as its premise that drug treatment, including aftercare, ends at some point and could be augmented by a continuing care model through linkage with primary care. Learning from chronic care treatment for other medical conditions, we see that a disease management approach linking patients to primary care following drug treatment, regardless of the length and intensity of initial treatment, has promise for our field. This extends the reach of what is offered in drug treatment-based aftercare, and may be of benefit to patients and health care systems. The study has important policy implications. If the intervention is successful, the CD Quality Improvement Committee will work with the CDRP and PC Chiefs in the NCKP region to implement it in other facilities.

17.5 Explain why the risks to subjects are reasonable:

Both study groups will receive usual care, and the experimental treatment in this study (Continuing Care) is considered a low-risk treatment method.

18.0 Data and Safety Monitoring Plan

18.1 Describe the plan for monitoring data and safety (Help Text updated 9/13):

Data and safety monitoring plan

The section below describes data and safety procedures it is anticipated will be followed in the proposed study. The Center's data safety and monitoring board will oversee the data and safety procedures which have been submitted to the National Institute on Drug Abuse in accordance with federal guidelines.

Treatment and compensation for injury. All participants in the study are patients in treatment for drug abuse in the CDRP in San Francisco, CA. Although not expected, any injury or condition experienced by a study participant will be treated in accordance with the participant's usual KP health coverage. Any injury or condition experienced by a KP employee as a result of being in this study will be treated in accordance with their usual health plan coverage and the worker's compensation coverage they have as part of their employment.

Protection of subject identity. To minimize risk to confidentiality throughout the study, all data will be identified only with participant numbers, and never with names or initials. All original patient data will be stored in locked cabinets in offices at the KP Division of Research (DOR). The list associating names and participant numbers will be stored in a separate locked cabinet, and only the Investigator will have access to the key. Research staff will not release any information to anyone other than the participant without the participant's written consent. No patient data will be removed from DOR. De-identified participant data will be analyzed at both DOR and UCSF, which have secure servers to store patient data. The data and safety monitoring mechanisms of the study are compliant with Health Insurance Portability and Accountability Act (HIPAA) regulations, and will be reviewed and approved by the IRB at KPNC.

Data acquisition and transmission and data monitoring plan. Study data sources include paper-and-pencil and computerized measures completed during in-person interviews and telephone interviews, and electronic medical record data. During the intervention study, the research technician will collect pre-treatment data in person using computerized instruments. Study interviewers based at DOR will collect post-treatment data over the telephone using computerized measures.

All participant data, including interview forms and instruments, pre- and post-treatment measures, treatment attendance records and tracking forms will be only be identified by subjects' study ID numbers, and will not contain subjects' names, medical record numbers, or other personal identifiers. Automated health record data will be generated by DOR staff programmers using only study ID numbers, and will not include personal identifiers. The forms linking the name of the subject with their study ID number will be kept in a locked file cabinet and in a password-protected computerized file. All original patient data will be kept in secure locked cabinets and password-protected computerized files at DOR.

Study interviewers will bring any questions concerning data collection and data quality to the immediate attention of Ms. Sterling (Project Manager) and Dr. Weisner (the component's principal investigator). In addition, data quality will be monitored by random and periodic inspection of the completed measures by the investigator, and reports made to Dr. Guydish, the Center PI. Any problems detected will be addressed and discussed with the interviewer. If necessary, re-training of the interviewer will be conducted. Data monitoring will be conducted by Stacy Sterling, the project coordinator, and Dr. Constance Weisner, the leader of the Drug and Alcohol Research Team at DOR, through quarterly data monitoring meetings throughout the study. During these meetings, Ms. Sterling and Dr. Weisner will review all data collected during participant interviews. The results of these data monitoring sessions will be reported to Dr. Guydish (PI of the Center), the KP and UCSF IRB and to the study's project officer at the National Institute on Drug Abuse (NIDA) as part of regular progress reports.

Safety monitoring plan and reporting mechanisms. We do not anticipate any injuries to incur in the course of the study. For any potential injury, patients are eligible for services based on their usual health plan coverage. The investigator is covered by liability insurance covering clinical research duties through the UCSF Department of Psychiatry. The study involves minimal risk to participants. There are no physical procedures to participants. The San Francisco CDRP has reviewed all study protocols, and we have procedures in place for clinical consultation in case of adverse events and in the case of individuals who may express suicidal ideation or severe depression. All adverse events occurring during the course of the study will be collected, documented and reported to the investigator by the research technician. The study interviewers will be trained by the investigator, a licensed clinical social worker with a doctorate in public health, to identify adverse reactions. A study participant may be withdrawn from the study if the investigator determines that it is the best decision in order to protect the safety of the study participant. The investigator will report any adverse reactions to the KP and University of California, San Francisco IRBs as well as to the NIDA project officer within 48 hours.

The investigator will be responsible for monitoring the safety and efficacy of the study, executing the data and safety monitoring plan, and complying with the reporting requirements. The investigator will provide a

summary of the data and safety monitoring report to NIDA on an annual basis as part of the progress report. The data and safety monitoring report will include the total number of subjects recruited and enrolled, retention rates, any quality assurance or regulatory issues that have taken place in the past year. The report will also include a summary of all serious and non-serious adverse events, and all actions or changes taken by the IRB with respect to the protocol. The data safety and management report to NIDA will also include, if applicable, the results of any efficacy data analysis conducted.
Study-specific potential adverse events. The study has targeted for treatment patients in outpatient chemical dependency treatment. There are no physical procedures to patients and risk of adverse events is considered low. The Kaiser Permanente's Northern California Institutional Review Board and UCSF's Committee on Human Research, as well as the Chemical Dependency Quality Improvement Committee and the Adult Medicine Chiefs Group will review all study protocols, and we have procedures in place for clinical consultation in case of adverse events. The San Francisco CDRP has clinical and emergency services to facilitate follow-up both during and outside of regular clinic hours, in the unlikely event that a study participant could pose a threat to self or others. The research interviewers and study clinicians will also contact the investigator immediately and follow established procedures in case of an adverse event.

Effective as soon as we receive approval (we have some flexibility to keep in place the old plan until the ISP starts).

Due to the low risk nature of this behavioral intervention we are formally changing the DSMB to an Independent Safety Monitor (ISM). We have secured Andrew Avins, MD, Internist (The Permanente Medical Group) as the ISM. Dr. Andrew Avins is a Research Scientist III, Division of Research, Kaiser Permanente Northern California, a Clinical Professor, Department of Medicine and Adjunct Professor, Department of Epidemiology and Biostatistics, University of California, San Francisco, a Physician, Urgent Care, Kaiser Permanente Medical Center, Oakland, CA and a Staff Physician, General Internal Medicine Section, Medical Service, Veterans Affairs Medical Center, San Francisco, CA . All study participant interviewing will be completed by the end of 2015.

18.2 This study requires a Data and Safety Monitoring Board:

- ○ Yes
- ◉ No or not sure

If **yes**, press **SAVE and CONTINUE** to move to the next section of the application.

18.3 If No, provide rationale:

- ◉ Social/Behavioral research
- ○ Phase I trial
- ○ Treatment IND/Compassionate Use Trial
- ○ Other (explain below)

If **Other,** explain:

19.0 Confidentiality and Privacy

19.1 Plans for maintaining privacy in the research setting:

All data will be identified only with participant numbers, never with names or initials. All data will be stored in locked cabinets in offices or encrypted computer files, with access only to Principal and Co-Investigators and

study staff at the Kaiser Division of Research (DOR) in Oakland, CA. Research staff will not release any information to anyone other than the participant without the participant's written consent.
This study will involve no disclosure of PHI. We will extract and analyze patient utilization and diagnostic data. Every safeguard will be taken to insure that this data is protected and that there is little risk of any breach of confidentiality.

19.2 Possible consequences to subjects resulting from a loss of privacy:

Because of the extensive safeguards in place, we do not anticipate any breaches of privacy. A potential consequence from breach of privacy could be embarrassment on the part of the participant.

19.3 Study data are:

- [ ] Derived from the Integrated Data Repository (IDR) or The Health Record Data Service (THREDS) at SFGH
- [x] Derived from a medical record (e.g. APeX, OnCore, etc. Identify source below)
- [ ] Added to the hospital or clinical medical record
- [ ] Created or collected as part of health care
- [ ] Used to make health care decisions
- [x] Obtained from the subject, including interviews, questionnaires
- [ ] Obtained from a foreign country or countries only
- [ ] Obtained from records open to the public
- [ ] Obtained from existing research records
- [ ] None of the above

If
derived from a medical record
, identify source:

KP has an entirely computerized inpatient and outpatient EMR.

19.4 Identifiers may be included in research records:

(•) Yes ( ) No

If **yes**, check all the identifiers that may be included:

- [x] Names
- [x] Dates
- [ ] Postal addresses
- [ ] Phone numbers
- [ ] Fax numbers
- [ ] Email addresses
- [ ] Social Security Numbers*
- [x] Medical record numbers
- [ ] Health plan numbers
- [ ] Account numbers
- [ ] License or certificate numbers
- [ ] Vehicle ID numbers
- [ ] Device identifiers or serial numbers
- [ ] Web URLs
- [ ] IP address numbers
- [ ] Biometric identifiers
- [ ] Facial photos or other identifiable images
- [ ] Any other unique identifier

* Required for studies conducted at the VAMC

19.5 Identifiable information might be disclosed as part of study activities:

(•) Yes ( ) No

If **yes**, indicate to whom identifiable information may be disclosed:

- [x] The subject's medical record
- [ ] The study sponsor
- [ ] Collaborators
- [ ] The US Food & Drug Administration (FDA)
- [ ] Others (specify below)
- [ ] A Foreign Country or Countries (specify below)

If **Others**, specify:

19.6 Indicate how data are kept secure and protected from improper use and disclosure (check all that apply): NOTE: Whenever possible, do not store subject identifiers on laptops, PDAs, or other portable devices. If you collect subject identifiers on portable devices, you MUST encrypt the devices.

- [ ] Data are stored securely in My Research
- [x] Data are coded; data key is destroyed at end of study
- [x] Data are coded; data key is kept separately and securely
- [x] Data are kept in a locked file cabinet
- [x] Data are kept in a locked office or suite
- [x] Electronic data are protected with a password
- [x] Data are stored on a secure network
- [ ] Data are collected/stored using REDCap or REDCap Survey
- [ ] Data are securely stored in OnCore

19.7 Additional measures to assure confidentiality and protect identifiers from improper use and disclosure, if any:

Only study staff will have access to the data collected as part of the study, and all employees who come in contact with these records sign an agreement to maintain confidentiality. All names are removed from research records; no identifying information will be used in any report or publication that is produced from this study. Data will only be presented in the aggregate. Data are kept under password protection on the secure, Division of Research (DOR) network.

19.8 This study may collect information that State or Federal law requires to be reported to other officials or ethically requires action:

(•) Yes ( ) No

Explain:

If a participant tells study personnel about incidents of child or elder abuse, expresses suicidal ideation, or threatens to harm someone, we will report it to the appropriate authorities.

19.9 This study will be issued a Certificate of Confidentiality:

(•) Yes ( ) No

20.0 Subjects

20.1 Check all types of subjects that may be enrolled:

- [ ] Inpatients
- [x] Outpatients
- [ ] Healthy volunteers
- [ ] Staff of UCSF or affiliated institutions

20.2 Additional vulnerable populations:

- [ ] Children
- [ ] Subjects unable to consent for themselves
- [ ] Subjects unable to consent for themselves (emergency setting)
- [ ] Subjects with diminished capacity to consent
- [ ] Subjects unable to read, speak or understand English
- [x] Pregnant women
- [ ] Fetuses
- [ ] Neonates
- [ ] Prisoners
- [ ] Economically or educationally disadvantaged persons
- [ ] Investigators' staff
- [ ] Students

Explain why it is appropriate to include the types of subjects checked above in this particular study:

Pregnant women are not excluded from treatment at the KP CDRP. Our sample of approximately 700 CDRP patients will be representative of the gender distribution of the patient population of the Kaiser Permanente Northern California San Francisco CDRP. Based on current enrollment, we estimate that the sample will be 42% female. We are not specifically recruiting pregnant women; however, there may be a few participants in the study who are pregnant. This is a low-risk behavioral study that does not include the use of any pharmaceuticals.

Describe the additional safeguards that have been included in the study to protect the rights and welfare of these subjects and minimize coercion or undue influence:

Participation in the study is completely voluntary. Eligible subjects will be offered participation in a study in which they will be assigned to Continuing Care or Usual Care (depending on whether the off or on condition is active). Both will be explained to them. It will be made clear to patients that their care in the CDRP is not contingent upon participation in the study, that all patients not interested in the study will receive usual services and that their care will not be affected. Research assistants will be placed in the clinic and will recruit, obtain informed consent, and facilitate the patient completing the computer-based baseline interview.

21.0 Inclusion of Pregnant Women, Fetuses, and/or Neonates

21.1 Review the regulatory categories by clicking on the Help bubble and identify all sections of 45 CFR 46 Subpart B under which you believe the research falls and provide study-specific information showing why the research falls within those sections:

- **[56 FR 28012, 28022, June 18, 1991; 56 FR 29756, June 28, 1991, as amended at 70 FR 36328, June 23, 2005]**
- **(b) The exemptions at §46.101(b)(1) through (6) are applicable to this subpart.**
- **§46.102 Definitions.**
- **(i) *Minimal risk* means that the probability and magnitude of harm or discomfort anticipated in the research are not in and of themselves than those ordinarily encountered in daily life or during the performance of routine physical or psychological examinations or tests.**

Because this study involves minimal risk to all participants, including pregnant women and their fetuses, and holds out the prospect of direct benefit to the woman and her fetus by potentially improving her overall health

and well-being, it meets the federal criteria for the inclusion of pregnant women.

22.0 Recruitment

22.1 * Methods (check all that apply):

☑ Study investigators (and/or affiliated nurses or staff) recruit their own patients directly in person or by phone.

☐ Study investigators recruit their own patients by letter. Attach the letter for review.

☐ Study investigators send a “Dear Doctor” letter to colleagues asking for referrals of eligible patients. If interested, the patient will contact the PI or the PI may directly recruit the patients (with documented permission from the patient). Investigators may give the referring physicians a study information sheet for the patients.

☐ Study investigators provide their colleagues with a “Dear Patient” letter describing the study. This letter can be signed by the treating physicians and would inform the patients how to contact the study investigators. The study investigators may not have access to patient names and addresses for mailing

☐ Advertisements, notices, and/or media used to recruit subjects. Interested subjects initiate contact with study investigators. Attach ads, notices, or media text for review. In section below, please explain where ads will be posted.

☐ Study investigators identify prospective subjects through chart review. (Study investigators request a Waiver of Authorization for recruitment purposes.)

☐ Large-scale epidemiological studies and/or population-based studies: Prospective subjects are identified through a registry or medical records and contacted by someone other than their personal physician. (Study investigators request a Waiver of Authorization for recruitment purposes.)

☐ Direct contact of potential subjects who have previously given consent to be contacted for participation in research. Clinic or program develops a CHR-approved recruitment protocol that asks patients if they agree to be contacted for research (a recruitment database) or consent for future contact was documented using the consent form for another CHR-approved study.

☐ Study investigators list the study on the School of Medicine list of UCSF Clinical Trials website or a similarly managed site. Interested subjects initiate contact with investigators.

☐ Study investigators recruit potential subjects who are unknown to them through methods such as snowball sampling, direct approach, use of social networks, and random digit dialing.

☐ Other

If **Other**, explain:

22.2 * How, when, and by whom eligibility will be determined:

The project coordinator will regularly obtain from the EMR a list for each primary therapist of patients completing their first week of CDRP treatment. This list will be shown to the primary therapist, who will identify anyone who should not be recruited to the study. The Program Coordinator will attend the weekly CDRP staff meeting and remind staff of study exclusion criteria. Research assistants will use the EMR to identify appointment dates of those eligible and meet patients at their CDRP treatment appointment. In prior studies, we have been able to conduct the interview that same day in most cases, and have been successful in making arrangements for the interview at their next CDRP appointment, with recruitment rates of 94%.[30, 165]

22.3 * How, when, where and by whom potential subjects will be approached:

Eligible subjects will be offered participation in a study in which they will be assigned to Continuing Care or Usual Care (depending on whether the off or on condition is active). Both will be fully explained to them. It will be made clear to patients that their participation is completely voluntary and that they can terminate participation in the study at any time. Their care in the CDRP is not contingent upon participation in the study, and all patients not interested in the study will also receive usual services. Research assistants will be placed in the clinic and will recruit, obtain informed consent, and facilitate the patient completing the computer-based baseline interview.

22.4 * Protected health information (PHI) will be accessed prior to obtaining consent:

(•) Yes ( ) No

23.0 Waiver of Consent/Authorization for Recruitment Purposes This section is required when study investigators (and/or affiliated nurses or staff) recruit their own patients directly.

23.1 * Study personnel need to access protected health information (PHI) during the recruitment process and it is not practicable to obtain informed consent until potential subjects have been identified:

(•) Yes

If **no**, a waiver of consent/authorization is NOT needed.

23.2 * A waiver for screening of health records to identify potential subjects poses no more than minimal risk to privacy for participants:

(•) Yes

If **no**, a waiver of authorization can NOT be granted.

23.3 * Screening health records prior to obtaining consent will not adversely affect subjects' rights and welfare:

(•) Yes

If **no**, a waiver of authorization can NOT be granted.

23.4 * Check all the identifiers that will be collected prior to obtaining informed consent:

- [x] Names
- [x] Dates
- [ ] Postal addresses
- [ ] Phone numbers
- [ ] Fax numbers
- [ ] Email addresses
- [ ] Social Security Numbers*
- [x] Medical record numbers
- [ ] Health plan numbers
- [ ] Account numbers
- [ ] License or certificate numbers
- [ ] Vehicle ID numbers
- [ ] Device identifiers or serial numbers
- [ ] Web URLs
- [ ] IP address numbers
- [ ] Biometric identifiers
- [ ] Facial photos or other identifiable images
- [ ] Any other unique identifier
- [ ] None

Note: HIPAA rules require that you collect the minimum necessary.

23.5 * Describe any health information that will be collected prior to obtaining informed consent:

The data collected will be names, medical record numbers, and appointment dates/times.
The project coordinator will regularly obtain from the EMR a list for each primary therapist of patients completing their first week of CDRP treatment. Research associates will use the EMR to identify appointment dates of those eligible and meet patients at their CDRP treatment appointment.

Note: HIPAA requires that you collect the minimum necessary.

23.6 * Describe your plan to destroy the identifiers at the earliest opportunity consistent with the research or provide a health or research justification for retaining the identifiers, or indicate and explain that retention is required by law:

Any data collected for subjects whose consent is not obtained will be destroyed (electronic files will be deleted; hard copies will be shredded).

24.0 Informed Consent

24.1 * Methods (check all that apply):

- [x] Signed consent will be obtained from subjects and/or parents (if subjects are minors)
- [ ] Verbal consent will be obtained from subjects using an information sheet or script
- [ ] Electronic consent will be obtained from subjects via the web or email
- [ ] Implied consent will be obtained via mail, the web or email
- [ ] Signed consent will be obtained from surrogates
- [ ] Emergency waiver of consent is being requested for subjects unable to provide consent
- [ ] Informed consent will not be obtained

24.2 * Process for obtaining informed consent:

Patients admitted to the CDRP day treatment program will be invited to participate in the study. Eligible subjects will be offered participation in a study in which they will be assigned to Continuing Care or Usual Care (depending on whether the "off" or "on" condition is active). Both will be explained to them. It will be made clear to patients that their care in the CDRP is not contingent upon participation in the study, that all patients not interested in the study will receive usual services and that their care will not be affected. The study's Research Associates will use the EMR to identify appointment dates of those eligible and meet patients at their CDRP treatment appointment. The Research Associates will be placed in the clinic and will recruit, obtain informed consent, and facilitate the patient completing the computer-based baseline interview. These Research Associates will be Division of Research staff members and not involved in the patient’s treatment or have decision making over this person. They will approach patients in the CDRP waiting area following their first CDRP group visit of the day, and ask if they can speak with them, and if they agree, will invite them to a private area to talk. The CDRP has agreed to provide a private office for this purpose. Patients who are under age 18, who have a diagnosis of mental retardation, dementia, or psychosis, as assessed by intake clinicians and who did not have a permanent residence in the San Francisco Bay Area will not be recruited. The study will be described to those eligible. They will be informed that participation is voluntary and that nonparticipation will not affect receiving services from the program. Patients agreeing to participate will be asked to sign a statement of informed consent. Informed consent will also cover the examination of medical records data. If the subject cannot read, the interviewer will read the form to him/her. Subjects may refuse to answer any of the specific questions in the interviews, can terminate interviews at any time, or refuse participation in any part of the project with which they feel uncomfortable. They will be given a name and telephone number of the Project Director and Principal Investigator and told they can contact them if they have any further questions.
Please note: We are proposing a two-tiered consent process. The first consent form will describe the confidentiality protections discussed above, as well as the additional protections provided by the Certificate

of Confidentiality. (please see “Basic” Consent Form)*
The second consent process, to conduct the Primary Care Linkage Call, will occur following participants' participation in the patient activation groups. The consent process for this activity will include an explanation that their Primary Care Provider is likely to document this conversation in their medical record, and as a result, their participation in Chemical Dependency Treatment will no longer be protected by the stricter confidentiality standards provided by the informational “firewall” as required by 42 CFR. It will be explained that there will thus be an increased risk of loss of confidentiality related to the Primary Care Linkage Call of allowing CD treatment to be included in their medical record, as a result of the conversation with their Primary Care Provider, and that, if documented in their regular medical record by their Primary Care Provider, CD treatment would also no longer be protected by law from disclosure by subpoena. (please see “Addendum” Consent Form)*

Primary care providers are not research subjects in the study. No analysis will be done evaluating their performance, or at the physician level at all. Similar to other quality improvement innovations made in primary care, they will be informed (during a regular lunch hour staff meeting) that a consultation appointment will be made in their schedules to have a phone call with the chemical dependency therapist and the patient (who is one of their patients). In Kaiser, physicians are commonly informed of system changes to increase clinical quality improvement in this manner, and telephone consultation appointments with other Kaiser clinicians are also common. The primary care providers are not subjects in the study. The intervention/training and study participation is at the level of patients – they are the ones being trained in how to use primary care.

24.3 * How investigators will make sure subjects understand the information provided to them:

Throughout the recruitment and consent process, the Research Associate will query the participant about their comprehension of all aspects of study participation, including risks and benefits. Patients agreeing to participate will be asked to sign a statement of informed consent. Informed consent will also cover the examination of medical records data. If the subject cannot read, the interviewer will read the form to him/her. Subjects may refuse to answer any of the specific questions in the interviews, can terminate interviews at any time, or refuse participation in any part of the project with which they feel uncomfortable. They will be given a name and telephone number of the Project Director and Principal Investigator and told they can contact them if they have any further questions. Only English-speaking patients will be recruited.

25.0 Financial Considerations

25.1 Subjects payment or compensation method (check all that apply):

Payments will be (check all that apply):

- ☐ Subjects will not be paid
- ☐ Cash
- ☐ Check
- ☐ Debit card
- ☑ Gift card
- ☐ Reimbursement for parking and other expenses
- ☐ Other:

Specify
Other
:

25.2 Describe the schedule and amounts of payments, including the total subjects can receive for completing the study. If deviating from recommendations in Subject Payment Guidelines, include specific justification below.

Participant reimbursement will be paid upon completion of the baseline, 6 month, 12 month, 24 month and

at 5 year interviews, at a rate of $50, $60, $60, $75 and $50 per interview, respectively. Participants providing urine samples will be reimbursed an additional $50. Participant reimbursements will be in the form of Target store gift cards.

25.3 Costs to Subjects: Will subjects or their insurance be charged for any study procedures?

○Yes ◉No

If
yes,
describe those costs below, and compare subjects' costs to the costs associated with alternative care off-study. Finally, explain why it is appropriate to charge those costs to the subjects.

26.0 CTSI Screening Questions

26.1 * This study will be carried out at one of the UCSF Clinical Research Services (CRS) centers or will utilize CRS services. CRS centers are at the following sites: SFGH Clinical Research Center Moffitt Adult Clinical Research Center Moffitt Hospital Pediatrics & NCRC Mount Zion Hospital Clinical Research Center Tenderloin Center CHORI Children's Hospital Pediatrics & Adult Clinical Research Center Kaiser Oakland Research Unit SF VA Medical Center Clinical Research Unit   Please note: Effective 3/1/14, the CRS form will no longer be completed and submitted in iRIS. The CRS budget request form can be found at: https://accelerate.ucsf.edu/files/crs/BudgetRequest2015.docx. Follow the instructions on the form to submit.   Even if you click 'Yes' to this question, the form will no longer proceed to the Clinical Research Services (CRS) Application Form section.

○Yes ◉No

26.2 This project involves community-based research:

○Yes ◉No

26.3 This project involves practice-based research:

○Yes ◉No

27.0 End of Study Application

27.1 End of Study Application Form   To continue working on the Study Application: Click on the section you need to edit in the left-hand menu. Remember to save through the entire Study Application after making changes.   If you are done working on the Study Application: Click Save and Continue. If this is a new study, you will automatically enter the Initial Review Submission Packet form, where you can attach consent forms or other study documents. Review the Initial Review Submission Checklist for a list of required attachments.   Answer all questions and attach all required documents to speed up your approval.

Review Response Submission Form - (Version 1.0)

1.0 Review Response Submission Form January 2017

1.1 Principal Investigator:

Constance M Weisner DrPH

1.2 Study Title:

Continuing Care Following Drug Abuse Treatment: Linkage with Primary Care

1.3 Study Number:

10-01606

1.4 Study Alias:

Continuing Care Linkage

1.5 Submission Components:

Instructions (new January 2017):

1. Review the stipulations below for change requests to submission components (submission forms, study application, consent forms, and/or study documents)

2. If changes are needed to individual submission components, follow the instructions below:

Making Changes to Forms - 'Study Application' and Submission Forms ('Initial Review Submission Form,' 'Continuing Review Form,' 'Modification Form,' etc.)

1. **Click the icon in the 'Revise/Attach' column to the left of the listed attachments** in the 'Submission Components' table be to open an editable version of the form (a revision)
2. **Make your changes, save through the form and then click 'Close Form'** to return to the 'Review Response Form'

Making Changes to Consent Forms and Study Documents

1. **Click the icon in the 'Revise/Attach' column to the left of the listed attachments** in the 'Submission Components' table be 'Ok' in the dialog box to confirm revising the component, then click 'Ok' on the message confirming a new version has been created
2. **Click the gray 'Check-out Document' button**
3. Follow the instructions on screen to **download the document, make your edits, and save the file** on your computer
4. Back in iRIS, **click the 'Complete Checkout' button** (when you return to the document details page, you will see red writing notin document is currently checked out by you)
5. **Click 'Check-in Document' and browse to the revised document** on your local machine
6. **Click the 'Unapproved Consent' or 'Unapproved Document' icon to verify** that you have uploaded the correct version, the **'Save Consent' (or 'Save Document') and then click the 'Back' button** to return to the 'Review Response Form'

Responding to Stipulations

1. **Summarize the changes made to forms and documents and/or answer any questions asked** in the text box below eac stipulation
2. Click '**Save Form'**
3. Click '**Signoff and Submit'** when all stipulations are addressed

NOTE: Please make sure that any tracked changes have been accepted for both consent forms and study documents. If tracked changes are submitted, they will show in the stamped PDF and you will have to submit a modification to get clean documents stamped. Consent forms should be uploaded as Word documents.

All Submission Components
Submission Form(s)
Review Response Submission Form - Version 1.0
Continuing Review Submission Form - Version 7.1
Continuing Review Submission Form - Version 7.0
Application
Study Application - Version 1.10
Attachment Form(s)

Outside Site Information - Version 1.9
Consent Form(s)
LINKAGE 5-year verbal consent script (English) - Version 1.0
Document(s)
Category : Other
thank you letter.gc - Version 1.0
linkage 5-year verbal consent script - Version 1.0
Category : Questionnaire
linkage 5 year quex - Version 1.0

2.0 Stipulations and Comments

2.1 In addition to making changes to the submission components, please briefly explain what changes were made:

Stipulations that must be addressed:

Stipulation 1 out of 3:

Description:
Please correct section 2.2 of the continuing review form to reflect the desire to continuing to contact patients and interview them by phone.

Stipulation Type:Stipulation must be addressed

Do you accept this stipulation?	N/A ○ Yes ◉ No ○
Provide an explanation on how you addressed this stipulation:	We have made the change to section 2.2 to reflect our desire to continue to contact participants by phone for a 5 year follow up interview.

Stipulation 2 out of 3:

Description:
Please attach the phone consent script in the informed consent section of iRIS.

Stipulation Type:Stipulation must be addressed

Do you accept this stipulation?	N/A ○ Yes ◉ No ○
Provide an explanation on how you addressed this stipulation:	I have attached the phone consent script under the informed consent section of the form. After talking with one of your analyst at the help desk, I checked the box for some subjects have been enrolled but we are not actively recruiting. We had previously checked subjects have been enrolled and study is now closed to accural. However, I was told by making that choice, the section to upload the consent form would not generate.

Stipulation 3 out of 3:

Description:
The phone consent script should include mention of UCSF, contact information for the study team, and the contact information for the UCSF IRB. It should also mention that questions about illegal activity will be asked and any risks involved.

Stipulation Type:Stipulation must be addressed

Do you accept this stipulation?	N/A ○ Yes ○ No ○
Provide an explanation on how you addressed this stipulation:	

Comments That Must Be Addressed With Follow-up Deadlines:

Comments:

3.0 Additional Modifications and/or Comments
3.1 Briefly describe any additional changes that were made and/or provide any other information you want the CHR have about the study or this submission:

4.0 Unresolved Stipulations/Comments
4.1
No Stipulation is outstanding.
