# Supplementary material for: Patterns of Health Care Use 5 Years After an Intervention Linking Patients in Addiction Treatment With a Primary Care Practitioner
Source: JAMA Netw Open. 2022 Nov 10;5(11):e2241338. doi: 10.1001/jamanetworkopen.2022.41338 (PMC9650610; doi:10.1001/jamanetworkopen.2022.41338)
Supplement: Supplement 2. — eFigure 1. Most Common Participant Emergency Department Diagnoses Coded as Emergent, Nonemergent, and Substance Use-Related eFigure 2. Estimated Percentage of Participants by Group With Health Care System Engagement per Year of Study Follow-up eTable 1. Health System Engagement Associated With Study Group Using Multiply Imputed Data eTable 2. Health System Engagement Associated With Study Group, Complete Cases, No Deaths eTable 3. Five-Year Health Care Use by Study Groups, Multiply Imputed Data eTable 4. Five-Year Health Care Use by Study Group, Complete Cases, No Deaths [file jamanetwopen-e2241338-s002.pdf]

Supplemental Online Content

Iturralde E, Weisner CM, Adams SR, et al. Patterns of health care use 5 years after an intervention linking patients in addiction treatment with a primary care practitioner. *JAMA Netw Open.* 2022;5(11):e2241338. doi:10.1001/jamanetworkopen.2022.41338

eFigure 1. Most Common Participant Emergency Department Diagnoses Coded as Emergent, Nonemergent, and Substance Use-Related

eFigure 2. Estimated Percentage of Participants by Group With Health Care System Engagement per Year of Study Follow-up

eTable 1. Health System Engagement Associated With Study Group Using Multiply Imputed Data

eTable 2. Health System Engagement Associated With Study Group, Complete Cases, No Deaths

eTable 3. Five-Year Health Care Use by Study Groups, Multiply Imputed Data

eTable 4. Five-Year Health Care Use by Study Group, Complete Cases, No Deaths

This supplemental material has been provided by the authors to give readers additional information about their work.

© 2022 Iturralde E et al. *JAMA Network Open.*

eFigure 1. Most Common Participant Emergency Department Diagnoses Coded as Emergent, Nonemergent, and Substance Use-Related

Classification	Primary Emergency Department Diagnosis	
Emergent	Chest pain, unspecified<br>Abdominal pain, unspecified site<br>Other chest pain<br>Syncope and collapse<br>Unspecified septicemia<br>Abdominal pain, other specified site<br>Headache<br>Issue of repeat prescriptions<br>Other malaise and fatigue<br>Lumbago<br>Nausea with vomiting<br>Painful respiration<br>Urinary tract infection, site not specified	Dizziness and giddiness<br>Diarrhea<br>Other convulsions<br>Unspecified abdominal pain<br>Unspecified gastritis and gastroduodenitis, without mention of hemorrhage<br>Diabetes mellitus without mention of complication, type II or unspecified type, not stated as uncontrolled<br>Epigastric pain
Non-emergent	Headache<br>Issue of repeat prescriptions<br>Other malaise and fatigue<br>Lumbago<br>Nausea with vomiting<br>Urinary tract infection, site not specified<br>Dizziness and giddiness<br>Diarrhea<br>Diabetes mellitus without mention of complication, type II or unspecified type, not stated as uncontrolled<br>Calculus of gallbladder without mention of cholecystitis, without mention of obstruction<br>Pneumonia, organism unspecified<br>Unspecified viral infection	Chronic or unspecified duodenal ulcer with hemorrhage, without mention of obstruction<br>Esophageal reflux<br>Low back pain<br>Migraine, unspecified, without mention of intractable migraine without mention of status migrainosus<br>Rash and other nonspecific skin eruption<br>Other and unspecified noninfectious gastroenteritis and colitis<br>Pain in joint, shoulder region<br>Pyelonephritis, unspecified
Substance Use-related	Alcohol withdrawal<br>Alcohol abuse, unspecified<br>Acute alcoholic intoxication in alcoholism, unspecified<br>Other and unspecified alcohol dependence, unspecified<br>Acute alcoholic intoxication in alcoholism, continuous<br>Alcohol dependence with withdrawal, unspecified<br>Alcohol abuse, continuous<br>Alcohol withdrawal delirium<br>Drug withdrawal<br>Other, mixed, or unspecified drug abuse, unspecified<br>Alcohol dependence, uncomplicated<br>Alcohol abuse with intoxication, unspecified	Alcohol abuse, uncomplicated<br>Alcohol dependence with intoxication, unspecified<br>Alcoholic gastritis, without mention of hemorrhage<br>Amphetamine and other psychostimulant dependence, unspecified<br>Alcohol dependence with withdrawal, uncomplicated<br>Other and unspecified alcohol dependence, episodic<br>Alcohol abuse with intoxication, uncomplicated<br>Alcohol abuse, episodic

© 2022 Iturralde E et al. *JAMA Network Open.*

eFigure 2. Estimated Percentage of Participants by Group With Health Care System Engagement per Year of Study Follow-up

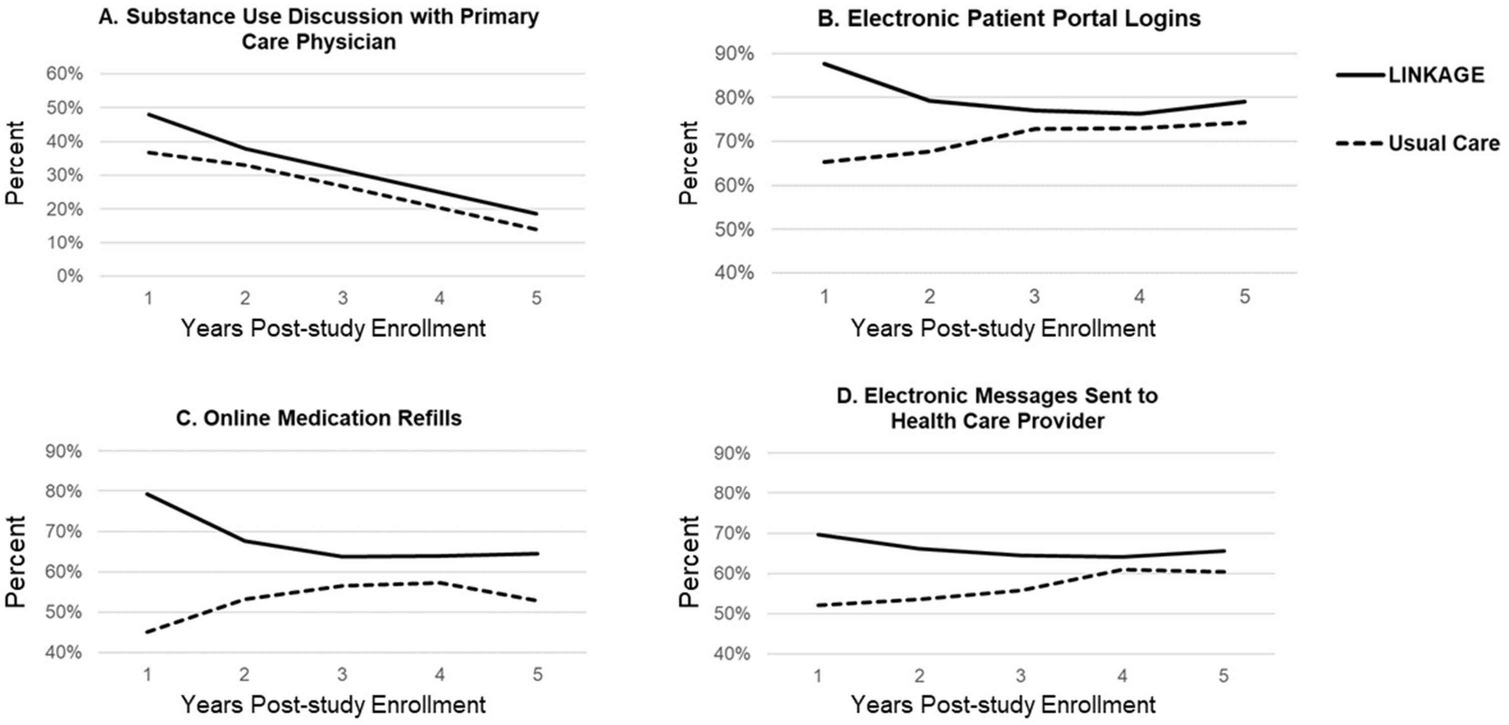

Marginal percent estimates by group were plotted from a modified Poisson generalized estimating equations model per outcome including time as a categorical variable, a time x group interaction term, and age, Charlson comorbidity index, and baseline emergent-type emergency department use.

© 2022 Iturralde E et al. *JAMA Network Open.*

eTable 1. Health System Engagement Associated With Study Group Using Multiply Imputed Data

Time	Group differences[b]											
	Substance use discussion with primary care provider[a]			Electronic patient portal logins			Online medication refills			Secure electronic messages sent to health care provider		
	RR	(95% CI)	*P* value	RR	(95% CI)	*P* value	RR	(95% CI)	*P* value	RR	(95% CI)	*P* value
Yr. 1	1.26	(0.99-1.60)	.06	1.32	(1.18-1.47)	< .001	1.70	(1.45-1.99)	< .001	1.32	(1.13-1.54)	< .001
Yr. 2	1.09	(0.83-1.44)	.54	1.16	(1.02-1.31)	.02	1.23	(1.05-1.46)	.01	1.21	(1.04-1.42)	.02
Yr. 3	—		—	1.06	(0.94-1.20)	.33	1.11	(0.93-1.32)	.24	1.12	(0.95-1.33)	.19
Yr. 4	—		—	1.07	(0.95-1.20)	.27	1.08	(0.91-1.27)	.38	1.05	(0.89-1.24)	.55
Yr. 5	1.26	(0.76-2.07)	.37	1.05	(0.94-1.17)	.39	1.13	(0.96-1.33)	.13	1.05	(0.90-1.23)	.54
All years (1-5)[c]	1.20	(0.99-1.45)	.07	1.12	(1.03-1.21)	.01	1.23	(1.09-1.38)	.001	1.12	(1.00-1.25)	.06

Time: year since study enrollment; RR: risk ratio; CI: confidence interval.

[a] Telephone interviews were conducted at 1, 2, and 5 years after study enrollment.

[b] Group differences were estimated from a modified Poisson regression model per follow-up time point, adjusting for baseline age, Charlson comorbidity index, and emergent-type ED use.

[c] Group differences were estimated from a modified Poisson generalized estimating equations model, adjusting for (linear) time and baseline age, Charlson comorbidity index, and emergent-type ED use.

© 2022 Iturralde E et al. *JAMA Network Open.*

eTable 2. Health System Engagement Associated With Study Group, Complete Cases, No Deaths

Time	Group differences[b]											
	Substance use discussion with primary care provider[a]			Electronic patient portal logins			Online medication refills			Secure messages sent to health care provider		
	RR	(95% CI)	*P* value	RR	(95% CI)	*P* value	RR	(95% CI)	*P* value	RR	(95% CI)	*P* value
Yr. 1	1.50	(1.08-2.07)	.02	1.35	(1.17-1.56)	< .001	2.05	(1.61-2.61)	< .001	1.47	(1.18-1.84)	.001
Yr. 2	1.24	(0.85-1.79)	.26	1.18	(1.00-1.38)	.048	1.28	(1.04-1.59)	.02	1.36	(1.09-1.69)	.01
Yr. 3	—		—	1.07	(0.93-1.23)	.35	1.12	(0.91-1.38)	.28	1.16	(0.94-1.44)	.17
Yr. 4	—		—	1.10	(0.95-1.27)	.21	1.20	(0.99-1.45)	.07	1.11	(0.91-1.34)	.30
Yr. 5	1.19	(0.59-2.41)	.62	1.06	(0.93-1.21)	.40	1.18	(0.95-1.46)	.13	1.09	(0.90-1.33)	.36
All years (1-5)[c]	1.32	(1.02-1.69)	.03	1.16	(1.03-1.30)	.01	1.42	(1.21-1.68)	< .001	1.22	(1.04-1.43)	.02

Time: year since study enrollment; RR: risk ratio; CI: confidence interval.
[a] Telephone interviews were conducted at 1, 2, and 5 years after study enrollment.
[b] Group differences were estimated from a modified Poisson regression model per follow-up time point, adjusting for baseline age, Charlson comorbidity index, and emergent-type ED use.
[c] Group differences were estimated from a modified Poisson generalized estimating equations model, adjusting for (linear) time and baseline age, Charlson comorbidity index, and emergent-type ED use.

© 2022 Iturralde E et al. *JAMA Network Open.*

eTable 3. Five-Year Health Care Use by Study Groups, Multiply Imputed Data

Health care service use	Group differences in annual change over the 5 years since study enrollment[a]			Time-adjusted group differences across 5 years post-study enrollment[b]		
	Risk Ratio	(95% CI)	*P* value	Risk Ratio	(95% CI)	*P* value
Primary care	1.03	(1.00-1.07)	.03	1.00	(0.94-1.06)	.97
Emergency department	0.97	(0.90-1.06)	.52	0.92	(0.76-1.10)	.34
Emergent	0.95	(0.78-1.16)	.63	1.03	(0.70-1.53)	.87
Non-emergent	1.00	(0.84-1.18)	.97	0.77	(0.58-1.01)	.06
Substance-related	0.86	(0.70-1.06)	.16	0.84	(0.51-0.99)	.48

CI: confidence interval.
[a] Group × linear time effect; modified Poisson generalized estimating equations models included (linear) time, a time x group interaction term, and baseline age, Charlson comorbidity index, and emergent-type emergency department use.
[b] Time-adjusted group effect; GEE models included (linear) time, no interaction term, and baseline age, Charlson comorbidity index, and emergent-type emergency department use.

© 2022 Iturralde E et al. *JAMA Network Open.*

eTable 4. Five-Year Health Care Use by Study Group, Complete Cases, No Deaths

Health care service use	Group differences in annual change over the 5 years since study enrollment[a]			Time-adjusted group differences across 5 years post-study enrollment[b]		
	Risk Ratio	(95% CI)	*P* value	Risk Ratio	(95% CI)	*P* value
Primary care	1.05	(1.01-1.09)	.01	0.98	(0.91-1.05)	.60
Emergency department	0.98	(0.87-1.10)	.69	0.89	(0.68-1.16)	.37
Emergent	0.81	(0.62-1.06)	.12	1.34	(0.79-2.28)	.28
Non-emergent	1.12	(0.89-1.39)	.34	0.60	(0.39-0.93)	.02
Substance-related	0.71	(0.56-0.92)	.01	0.73	(0.35-1.52)	.40

CI: confidence interval.

[a] Group × linear time effect; modified Poisson generalized estimating equations models included (linear) time, a time x group interaction term, and baseline age, Charlson comorbidity index, and emergent-type emergency department use.

[b] Time-adjusted group effect; modified Poisson generalized estimating equations models included (linear) time, no interaction term, and baseline age, Charlson comorbidity index, and emergent-type emergency department use.

© 2022 Iturralde E et al. *JAMA Network Open.*
