# Supplementary material for: Patterns of Health Care Use 5 Years After an Intervention Linking Patients in Addiction Treatment With a Primary Care Practitioner
Source: JAMA Netw Open. 2022 Nov 10;5(11):e2241338. doi: 10.1001/jamanetworkopen.2022.41338 (PMC9650610; doi:10.1001/jamanetworkopen.2022.41338)
Supplement: Supplement 3. — Data Sharing Statement [file jamanetwopen-e2241338-s003.pdf]

Data Sharing Statement

Iturralde. Patterns of Health Care Use 5 Years After an Intervention Linking Patients in Addiction Treatment With a Primary Care Practitioner. *JAMA Netw Open*. Published November 10, 2022. doi:10.1001/jamanetworkopen.2022.41338

Data
Data available: No
